# Supplementary material for: Targeted protein degradation in Escherichia coli using CLIPPERs
Source: EMBO Rep. 2025 Jun 25;26(16):3994–4016. doi: 10.1038/s44319-025-00510-9 (PMC12373786; doi:10.1038/s44319-025-00510-9)
Supplement: Supplementary file 1 — Appendix [file 44319_2025_510_MOESM1_ESM.pdf]

Targeted Protein Degradation in *Escherichia coli* using CLIPPERs.

Matylda Anna Izert-Nowakowska[1], Maria Magdalena Klimecka[1], Anna Antosiewicz[1], Karol Wróblewski[2], Jakub Józef Kowalski[1], Katarzyna Justyna Bandyra[1], Tomasz Góral[3], Sebastian Kmiecik[2], Remigiusz Adam Serwa[4], Maria Wiktoria Górna[1]*

[1] Structural Biology Group, Biological and Chemical Research Centre, Faculty of Chemistry, University of Warsaw, Warsaw, Poland

[2] Biological and Chemical Research Centre, Faculty of Chemistry, University of Warsaw, Warsaw, Poland

[3] Cryomicroscopy and Electron Diffraction Core Facility, Centre of New Technologies, University of Warsaw, Warsaw, Poland

[4] IMol Polish Academy of Sciences, Warsaw, Poland

*E-mail: mw.gorna@uw.edu.pl

Appendix

Appendix Figure S1. Growth curves of bacteria grown at 30 °C in presence of expression-inducing arabinose 1

Appendix Figure S2. Changes of protein levels upon expression of control peptides 2

Appendix Figure S3. Results of peptide modelling into the cryo-EM map. 2

Appendix Table S1. Binding parameters between peptides and their binding partners ClpX and GroEL. 3

Appendix Table S2. List of DNA constructs used in the study 3

Appendix Table S3. List of oligonucleotides used for obtaining DNA constructs 5

Appendix Table S4. List of synthetic peptides used in this study for BLI and cryo-EM experiments 6

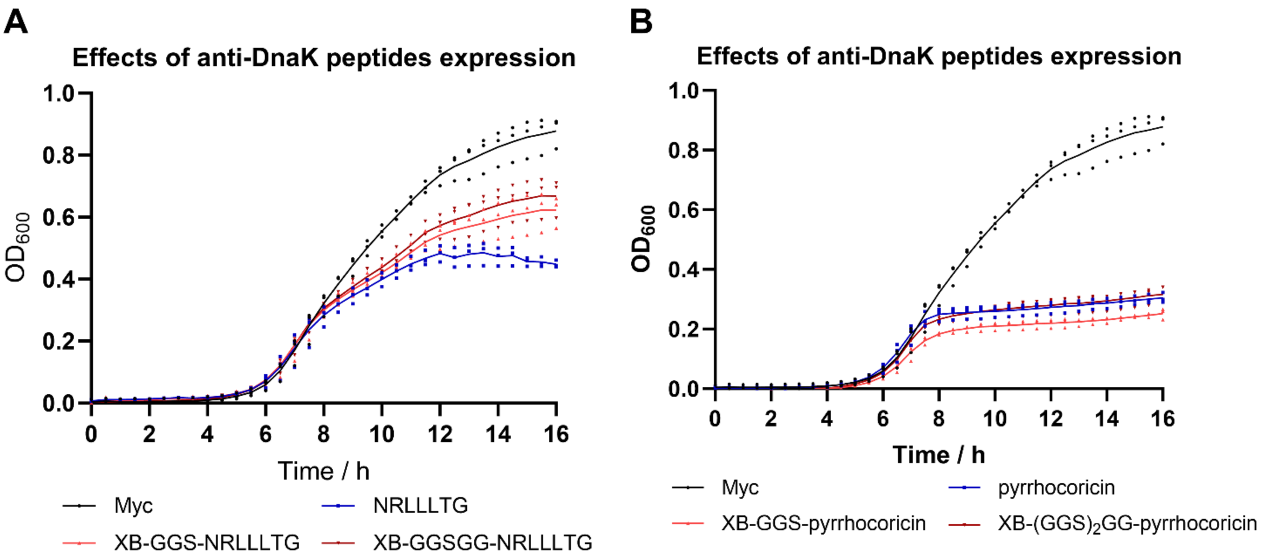

Appendix Figure S1. Growth curves of bacteria grown at 30 °C in presence of expression-inducing arabinose. The assay was performed for bacteria transformed with pBAD-Myc plasmids encoding DnaK-targeting CLIPPERs with (A) NRLLLTG peptide as bait, or (B) pyrrhocoricin peptide as bait. Technical triplicates were used, the lines connect their mean values.

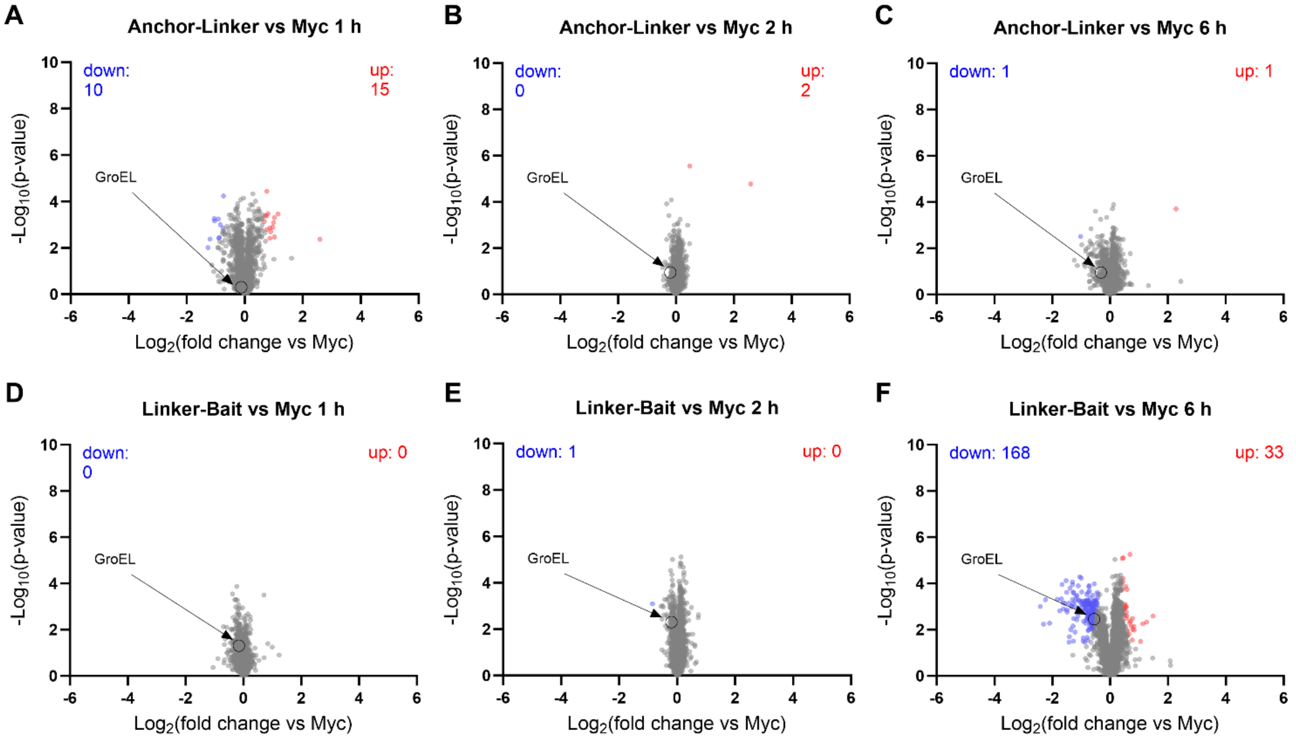

Appendix Figure S2. Changes of protein levels upon expression of control peptides. Quantitative mass spectrometry results (TMT-MS) for two GroTAC controls (complementing the GroTAC experiments shown in Figure 3). (A) – (C) Volcano plots representing the effects of Anchor-Linker peptide at different time points. (D) – (F) Volcano plots representing the effects of Linker-Bait peptide at different time points. GroEL is indicated with an arrow and a circle. All Volcano plots display the log2 fold change (log2FC, x axis) against the t-test-derived −log10 statistical p-value (y axis) for protein groups detected in total lysates of *E.coli* expressing Anchor-Linker or Linker-Bait compared to *E.coli* expressing control peptide, Myc, by LC-MS/MS analysis (n = 3). Student's t-test (two-sided, unpaired) was performed for the statistical analysis. Proteins levels found upregulated, downregulated (q-val < 0.05), and unchanged are indicated as red, blue, and gray dots, respectively.

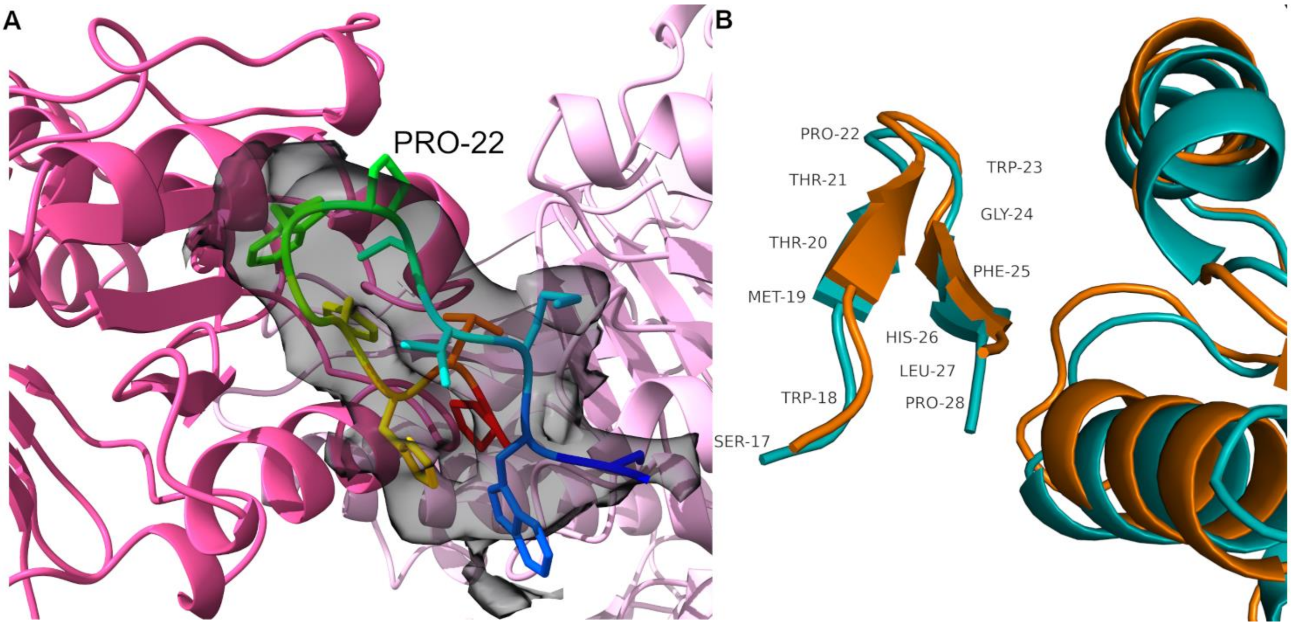

Appendix Figure S3. Results of peptide modelling into the cryo-EM map. (A) Fitting of the GroTAC3 peptide (rainbow) into the obtained electrostatic potential density; the two neighbouring GroEL monomers comprising the peptide binding pocket are colored in different shades of pink. (B) Comparison of the backbone of GroEL-binding part of GroTAC peptide obtained from Cryo-EM (cyan; PDB ID 8S32) with the crystallographic structure of SBP in complex with GroEL (orange, PDB ID 1MNF).

Appendix Table S1. Binding parameters between peptides and their binding partners ClpX and GroEL. The results were calculated from BLI experiments presented in Figure 4 and Figure EV5. The parameters were calculated assuming 1:1 binding stoichiometry to avoid overfitting of the data.

**Analyte**	**Ligand**	**K_D (M)**	**k_a (1/Ms)**	**k_{dis} (1/s)**	**Full X^2**	**Full R^2**
ClpX	His-SUMO-XB-GGS ("Anchor-Linker")	6.48E-08 ± 2.69E-10	7.65E+04 ± 2.83E+02	4.95E-03 ± 9.39E-06	12.878	0.996
ClpX	His-SUMO-XB-GGS-SBP ("GroTAC1")	7.04E-08 ± 5.32E-10	1.09E+05 ± 7.72E+02	7.69E-03 ± 2.06E-05	13.202	0.987
GroEL	His-SUMO-GGS-SBP ("Linker-Bait")	9.36E-08 ± 1.37E-09	9.82E+04 ± 1.36E+03	9.19E-03 ± 4.11E-05	47.844	0.931
GroEL	His-SUMO-XB-GGS-SBP ("GroTAC1")	1.54E-07 ± 1.45E-09	2.66E+04 ± 2.21E+02	4.09E-03 ± 1.84E-05	8.5745	0.959
GroTAC1 (synthetic)	His-ZBD	3.54E-08 ± 1.67E-10	2.26E+04 ± 6.70E+01	7.98E-04 ± 2.92E-06	19.622	0.989
GroTAC2 (synthetic)	His-ZBD	5.26E-08 ± 2.84E-10	3.14E+04 ± 1.40E+02	1.65E-03 ± 5.02E-06	27.907	0.980
GroTAC3 (synthetic)	His-ZBD	8.24E-08 ± 4.59E-10	1.11E+04 ± 4.30E+01	9.15E-04 ± 3.65E-06	14.097	0.989
GroEL-GroTAC1 complex (using increasing concentrations of the synthetic GroTAC1)	His-ZBD	1.65E-07 ± 1.71E-09	1.09E+04 ± 9.32+01	1.80E-03 ± 1.05E-05	295.552	0.973
GroEL-GroTAC2 complex (using increasing concentrations of the synthetic GroTAC2)	His-ZBD	3.08E-07 ± 3.79E-09	9.32E+03 ± 1.05E+02	2.87E-03 ± 1.45E-05	238.550	0.963

Appendix Table S2. List of DNA constructs used in the study

**Construct name**	**Purpose**	**Backbone**	**Insert**	**Source**
pBAD-6xHis-SUMO-ClpX	expression and purification of ClpX	pBAD	His tag - SUMO tag - ClpX	[33]
pET28a-6xHis-SUMO-ClpP	expression and purification of ClpP	pET28a	His tag - SUMO tag - ClpP	[33]
pET28a-6xHis-SUMO-SspB	expression and purification of SspB	pET28a	His tag - SUMO tag - SspB	[33]
pET28a-His-ZBD	expression and purification of ZBD	pET28a	His tag - ZBD	this study
pET28a-GroEL	expression and purification of GroEL	pET28a	GroEL	this study
pET28a-6xHis-SUMO-XB-GGS	expression and purification of anchor-linker	pET28a	His tag - SUMO tag - CYRGGRPALRVVK - GGS	this study
pET28a-6xHis-SUMO-GGS-SBP	expression and purification of linker-bait against GroEL	pET28a	His tag - SUMO tag - GGS - SWMTTPWGFHLP	this study

pET28a-6xHis-SUMO-XB-GGS-SBP	expression and purification of GroTAC1	pET28a	His tag - SUMO tag - CYRGGRPALRVVK - GGS - SWMTTPWGFHLP	this study
pBAD-His-TEV-cAbGFP	expression and purification of cAbGFP	pBAD	His tag - TEV cleavage site - cAbGFP	this study
pBAD-6xHIS-TEV	eGFP-anchor stability assay	pBAD	His tag - T7 gene leader - TEV cleavage site	[33]
pBAD-6xHis-TEV-eGFP	eGFP-anchor stability assay	pBAD	His tag - T7 gene leader - TEV cleavage site - eGFP	Addgene #54762
pBAD-6xHis-TEV-eGFP-ssrA	eGFP-anchor stability assay	pBAD	His tag - T7 gene leader - TEV cleavage site - eGFP - AANDENYALAA	[33]
pBAD-6xHis-TEV-eGFP-AANDENY	eGFP-anchor stability assay	pBAD	His tag - T7 gene leader - TEV cleavage site - eGFP - AANDENY	[33]
pBAD-6xHis-TEV-eGFP-2xAANDENY	eGFP-anchor stability assay	pBAD	His tag - T7 gene leader - TEV cleavage site - eGFP - AANDENYAANDENY	[33]
pBAD-6xHis-TEV-eGFP-XB	eGFP-anchor stability assay	pBAD	His tag - T7 gene leader - TEV cleavage site - eGFP - CYRGGRPALRVVK	this study
pBAD-6xHis-TEV-eGFP-sXB	eGFP-anchor stability assay	pBAD	His tag - T7 gene leader - TEV cleavage site - eGFP - ALRVVK	this study
pBAD-6xHis-TEV-eGFP-IGF	eGFP-anchor stability assay	pBAD	His tag - T7 gene leader - TEV cleavage site - eGFP - GIGFGATVK	this study
pBAD-6xHis-TEV-eGFP-IGL	eGFP-anchor stability assay	pBAD	His tag - T7 gene leader - TEV cleavage site - eGFP - KSIGLIHQD	this study
pBAD-myc	testing CLIPPERs in bacteria (control)	pBAD	Myc tag	this study
pBAD-myc-XB-GGS	testing CLIPPERs in bacteria (anchor control)	pBAD	Myc tag - CYRGGRPALRVVK - GGS	this study
pBAD-myc-GGS-SBP	testing CLIPPERs in bacteria (GroEL bait control)	pBAD	Myc tag - GGS - SWMTTPWGFHLP	this study
pBAD-myc-XB-GGS-SBP	testing CLIPPERs in bacteria (GroTAC1)	pBAD	Myc tag - CYRGGRPALRVVK - GGS - SWMTTPWGFHLP	this study
pBAD-myc-XB-GGSGGSGG-SBP	testing CLIPPERs in bacteria (GroTAC2)	pBAD	Myc tag - CYRGGRPALRVVK - GGSGGSGG - SWMTTPWGFHLP	this study
pBAD-myc-NRLLLTG	testing CLIPPERs in bacteria (DnaK bait control)	pBAD	Myc tag - NRLLLTG	this study
pBAD-myc-XB-GGS-NRLLLTG	testing CLIPPERs in bacteria (DnaK degrader 1)	pBAD	Myc tag - CYRGGRPALRVVK - GGS - NRLLLTG	this study
pBAD-myc-XB-GGSGG-NRLLLTG	testing CLIPPERs in bacteria (DnaK degrader 2)	pBAD	Myc tag - CYRGGRPALRVVK - GGSGG - NRLLLTG	this study

pBAD-myc-pyrrhocoricin	testing CLIPPERs in bacteria (pyrrhocoricin control)	pBAD	Myc tag - VDKGSYLPRPTPPRPIYNRN	this study
pBAD-myc-XB-GGS-pyrrhocoricin	testing CLIPPERs in bacteria (pyrrhocoricin degrader 1)	pBAD	Myc tag - CYRGGRPALRVVK - GGS - VDKGSYLPRPTPPRPIYNRN	this study
pBAD-myc-XB-GGSGGSGG-pyrrhocoricin	testing CLIPPERs in bacteria (pyrrhocoricin degrader 2)	pBAD	Myc tag - CYRGGRPALRVVK - GGSGGSGG - VDKGSYLPRPTPPRPIYNRN	this study

Appendix Table S3. List of oligonucleotides used for obtaining DNA constructs

Plasmid name	Template	Forward primer	Reverse primer
pBAD-eGFP-XB	pBAD-eGFP	GCATTACGCGTTGTGAAGTAAGAATTCGAAGCTTGGCTG	CGGTCGACCACCGCGGTAGCACTTGTACAGCTCGTCCATG
pBAD-eGFP-sXB	pBAD-eGFP	TGTGAAGTAAGAATTCGAAGCTTGGCTG	ACGCGTAAGGCCTTGTACAGCTCGTCCATG
pBAD-eGFP-IGF	pBAD-eGFP	CGCGACGGTAAAATAAGAATTCGAAGCTTGGC	CCAAAACCAATGCCCTTGTACAGCTCGTCCATG
pBAD-eGFP-IGL	pBAD-eGFP	TATCCACCAGGATTAAGAATTCGAAGCTTGGC	AGACCAATGGATTTCTTGTACAGCTCGTCCATG
pBAD-Myc-eGFP	pBAD-eGFP	AGCGAAGAAGATCTGGGCTCGAGCATGGTGAGC	AATCAGTTTCTGTTCCATATGTATATCTCCTTCTTAAAGTTAAACAAAATTATTTCTAG
pBAD-Myc-eGFP-XB	pBAD-eGFP-XB	AGCGAAGAAGATCTGGGCTCGAGCATGGTGAGC	AATCAGTTTCTGTTCCATATGTATATCTCCTTCTTAAAGTTAAACAAAATTATTTCTAG
pBAD-Myc	pBAD-Myc-eGFP	TAAGAATTCGAAGCTTGGC	GCTCGAGCCCAGATCTTC
pBAD-Myc-XB	pBAD-Myc-eGFP-XB	TGCTACCGCGGTGGTCGA	GCTCGAGCCCAGATCCTCTTC
pBAD-Myc-XB-GGS-SBP	pBAD-Myc-XB-GGS-SSB	TGGGGTTTTCACCTGCCCTAAGAATTCGAAGCTTGGC	AGGCGTAGTCATCCACGAGCTACCGCCCTTCACAAC
pBAD-Myc-XB-GGS	pBAD-Myc-XB-GGS-SSB	TAAGAATTCGAAGCTTGGC	GCTACCGCCCTTCACAAC
pBAD-Myc-GGS-SBP	pBAD-Myc-XB-GGS-SBP	GGCGGTAGCTCGTGGATG	GCTCGAGCCCAGATCCTCTTC
pBAD-Myc-XB-GGSGGSGG-SBP	pBAD-Myc-XB-GGSGGSGG-SSB	TGGGGTTTTCACCTGCCCTAAGAATTCGAAGCTTGGC	AGGCGTAGTCATCCACGAACCGCCGCTACCGCCGCT
pBAD-Myc-NRLLLTG	pBAD-Myc	GCTGACTGGTTAAGAATTCGAAGCTTGGCTG	AGCAGACGGTTGCTCGAGCCCAGATCTTC
pBAD-Myc-XB-GGS-NRLLLTG	pBAD-Myc-XB-GGS-SSB	GCTGACTGGTTAAGAATTCGAAGCTTGGCTG	AGCAGACGGTTGCTACCGCCCTTCACAAC
pBAD-Myc-XB-GGSGG-NRLLLTG	pBAD-Myc-XB-GGSGG-SSB	GCTGACTGGTTAAGAATTCGAAGCTTGGCTG	AGCAGACGGTTACCGCCGCTACCGCCGCT

pBAD-Myc-pyrrhocoricin	pBAD-Myc	ACGCCACCACGCCCCATCTACAACCGTAATTAAGAATTCGAAGCTTGGCTG	CGGGCGCGGTAAGTAACTGCCCTTATCCACGCTCGAGCCCAGATCTTC
pBAD-Myc-XB-GGS-pyrrhocoricin	pBAD-Myc-XB-GGS-SSB	ACGCCACCACGCCCCATCTACAACCGTAATTAAGAATTCGAAGCTTGGCTG	CGGGCGCGGTAAGTAACTGCCCTTATCCACGCTACCGCCCTTCACAAC
pBAD-Myc-XB-GGSGGSGG-pyrrhocoricin	pBAD-Myc-XB-GGSGGSGG-SSB	ACGCCACCACGCCCCATCTACAACCGTAATTAAGAATTCGAAGCTTGGCTG	CGGGCGCGGTAAGTAACTGCCCTTATCCACACCGCCGCTACCGCCGCT
pET28a-HisSUMO-GroEL	Genomic DNA from *E. coli* Top10	TGATTGAAGTCTACCAGGAACAAACCGGTGGATCCATGGCAGCTAAAGACGTAAAATTCG	CGGATCTCAGTGGTGGTGGTGGTGGTGCTCGAGTTACATCATGCCGCCCATGCC
pET28a-GroEL	pET28a-His SUMO-GroEL	ATGGCAGCTAAAGACGTA	GGTATATCTCCTTCTTAAAGTTAAAC
pET28a-HisSUMO-ZBD	pET28a-SUMO-ClpX	TGATTGAAGTCTACCAGGAACAAACCGGTGGATCCACAGATAAACGCAAAGATGGCT	CGGATCTCAGTGGTGGTGGTGGTGGTGCTCGAGTTAAAATCTCTTCGCGAATGATGTCG
pET28a-His-ZBD	pET28a-HisSUMO-ZBD	GGTGGATCCACAGATAAACG	GTGATGATGATGATGATGG
pET28a-HisSUMO-XB-GGS	- (annealed oligos used as insert)	CCGGTGGATCCTGCTACCGCGGTGGTCGACCGGCATTACGCGTTGTGAAGGGCGGTAGCTAACTCGAGCACC	GGTGCTCGAGTTAGCTACCGCCCTTCACAACGCGTAATGCCGGTCGACCACCGCGGTAGCAGGATCCACCGG
pET28a-HisSUMO-GGS-SBP	- (annealed oligos used as insert)	CCGGTGGATCCGGCGGTAGCTCGTGGATGACTACGCCTTGGGGTTTTCACCTGCCCTAACTCGAGCACC	GGTGCTCGAGTTAGGGCAGGTGAAAACCCCAAGGCGTAGTCATCCACGAGCTACCGCCGGATCCACCGG
pET28a-HisSUMO-XB-GGS-SBP	- (annealed oligos used as insert)	CCGGTGGATCCTGCTACCGCGGTGGTCGACCGGCATTACGCGTTGTGAAGGGCGGTAGCTCGTG	CGTAATGCCGGTCGACCACCGCGGTAGCAGGATCCACCGG
		GATGACTACGCCTTGGGGTTTTCACCTGCCCTAACTCGAGCACC	GGTGCTCGAGTTAGGGCAGGTGAAAACCCCAAGGCGTAGTCATCCACGAGCTACCGCCCTTCACAACG

Appendix Table S4. List of synthetic peptides used in this study for BLI and cryo-EM experiments

**Peptide name**	**Peptide sequence**	**Experiments in this study**
GroTAC1	CYRGGRPALRVVKGGSSWMTTPWGFHLP	Biolayer Interferometry
GroTAC2	CYRGGRPALRVVKGGSGGSGGSWMTTPWGFHLP	Biolayer Interferometry
Anchor-Linker	CYRGGRPALRVVKGGSGGSGG	Biolayer Interferometry
Linker-Bait	GGSGGSGGSWMTTPWGFHLP	Biolayer Interferometry
GroTAC3	CYRGGRPALRVVK-(PEG)$_3$-SWMTTPWGFHLP	Biolayer Interferometry, Cryo-EM
