# Supplementary material for: Targeted protein degradation in Escherichia coli using CLIPPERs
Source: EMBO Rep. 2025 Jun 25;26(16):3994–4016. doi: 10.1038/s44319-025-00510-9 (PMC12373786; doi:10.1038/s44319-025-00510-9)
Supplement: Supplementary file 5 — Source data Fig. 2 [file 44319_2025_510_MOESM5_ESM.zip › Fig2/Fig2B/Fig2B.pdf]

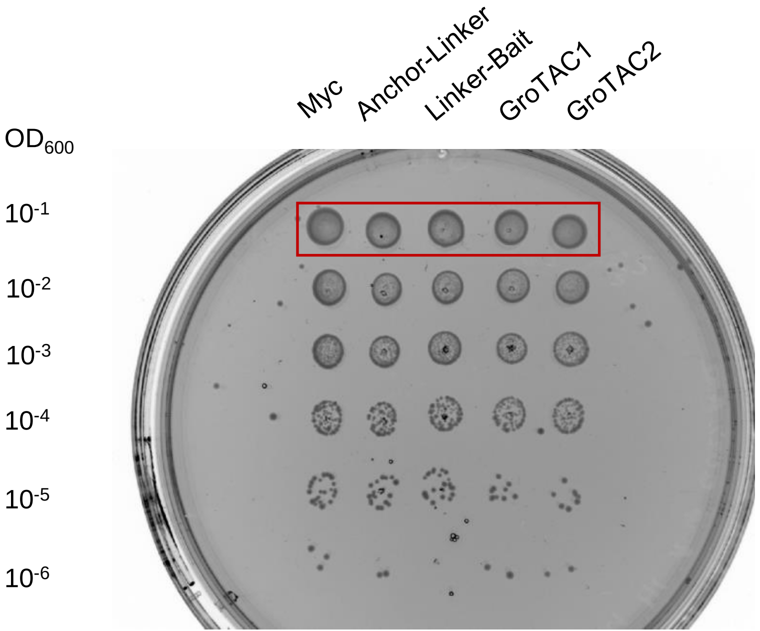

Myc
Anchor-Linker
Linker-Bait
GroTAC1
GroTAC2
OD_{600}
10^{-1}
10^{-2}
10^{-3}
10^{-4}
10^{-5}
10^{-6}

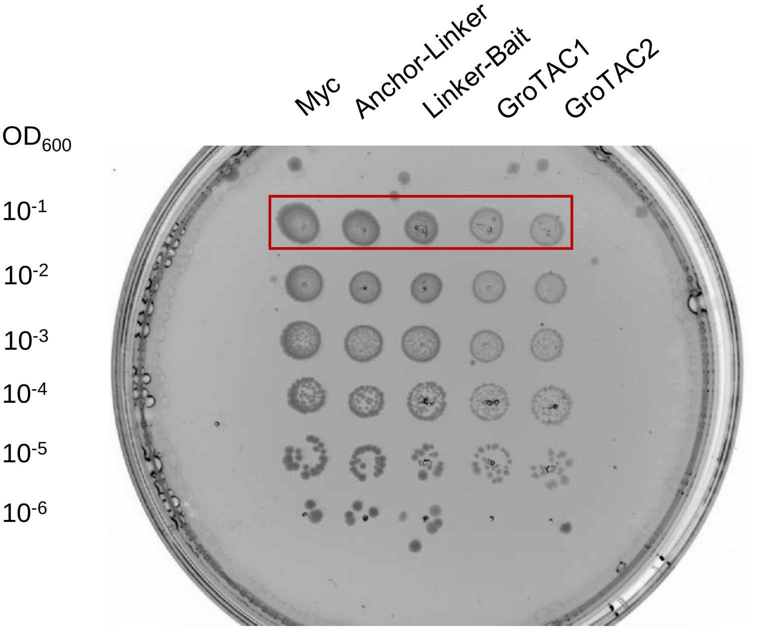

Myc
Anchor-Linker
Linker-Bait
GroTAC1
GroTAC2
OD600
10-1
10-2
10-3
10-4
10-5
10-6

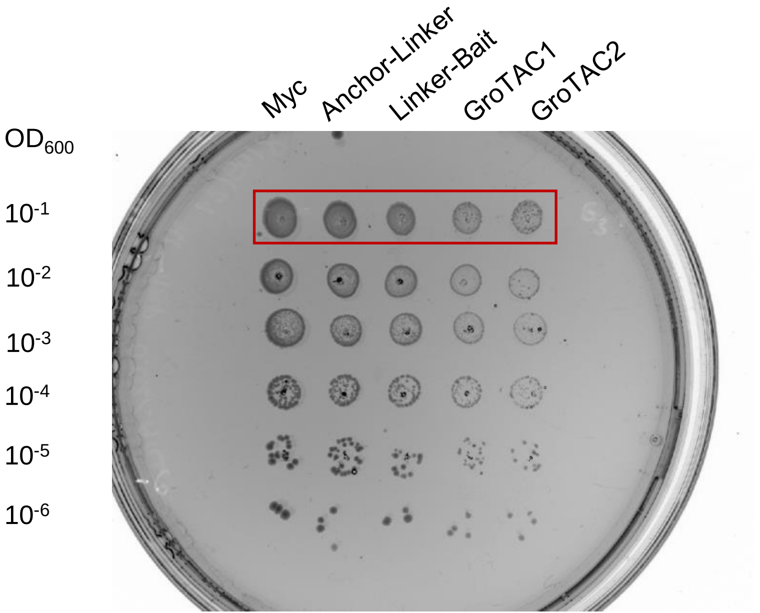

Myc
Anchor-Linker
Linker-Bait
GroTAC1
GroTAC2
OD600
10-1
10-2
10-3
10-4
10-5
10-6

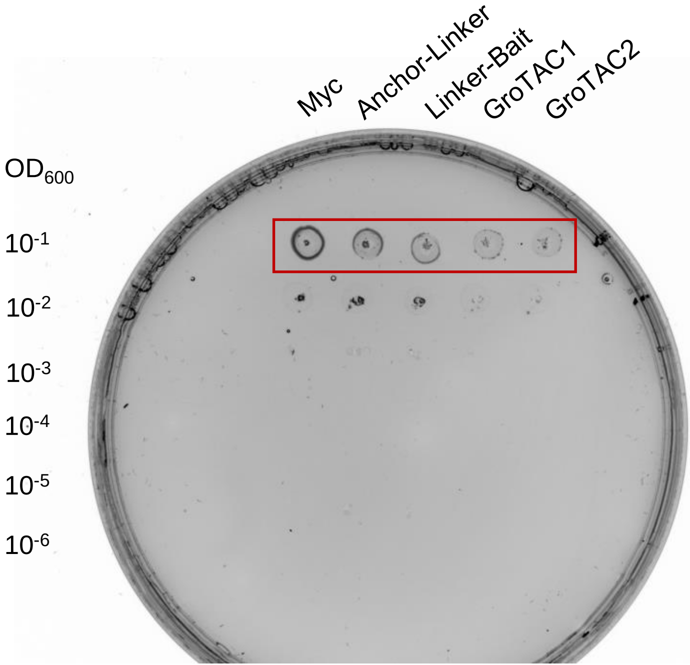

Myc
Anchor-Linker
Linker-Bait
GroTAC1
GroTAC2
OD600
10-1
10-2
10-3
10-4
10-5
10-6
