# Supplementary figures and images for: Targeted protein degradation in Escherichia coli using CLIPPERs
Source: EMBO Rep. 2025 Jun 25;26(16):3994–4016. doi: 10.1038/s44319-025-00510-9 (PMC12373786; doi:10.1038/s44319-025-00510-9)

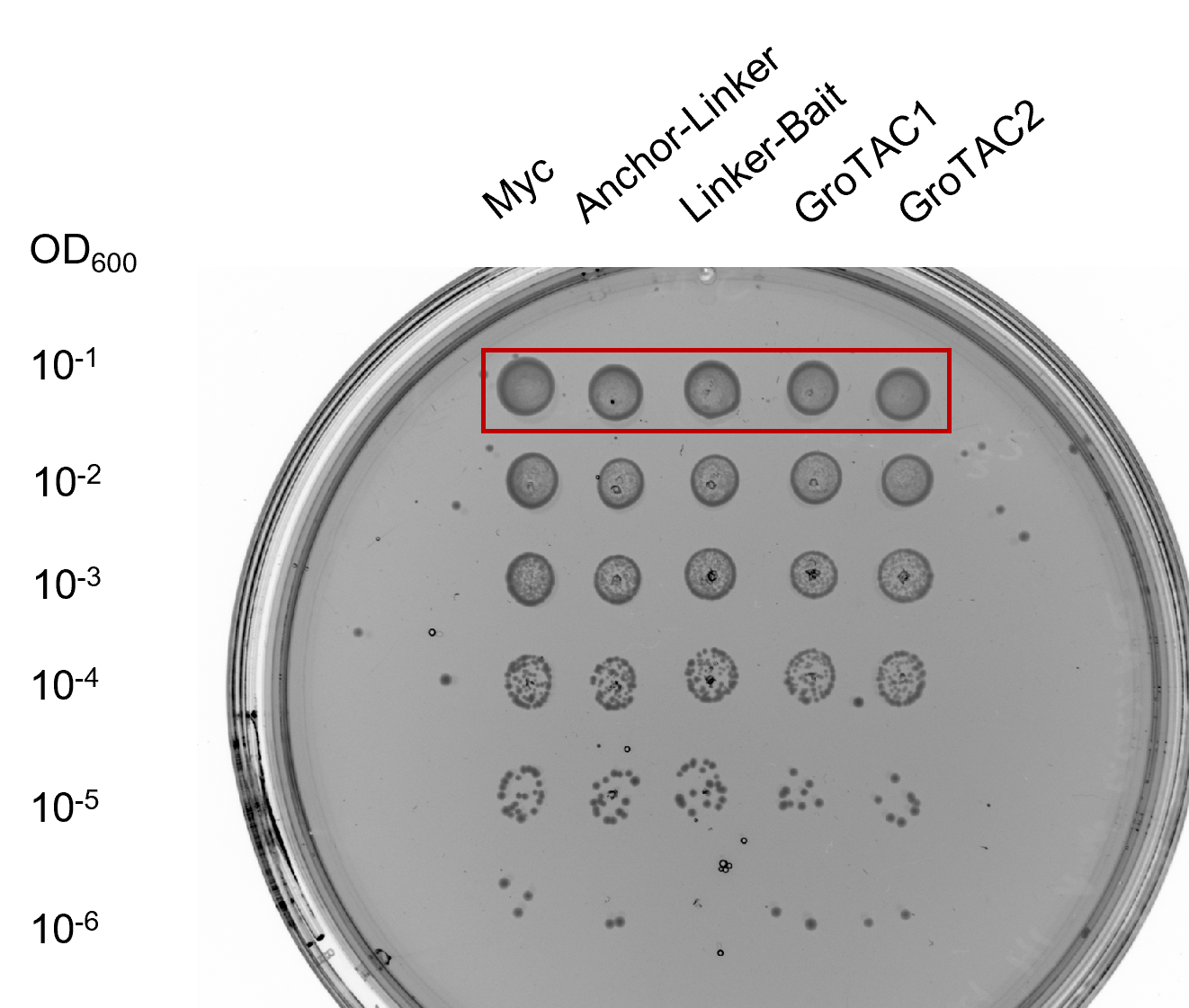

Supplement: Supplementary file 5 — Source data Fig. 2 [file 44319_2025_510_MOESM5_ESM.zip › Fig2/Fig2B/Plate_drop_test_20C.tif]

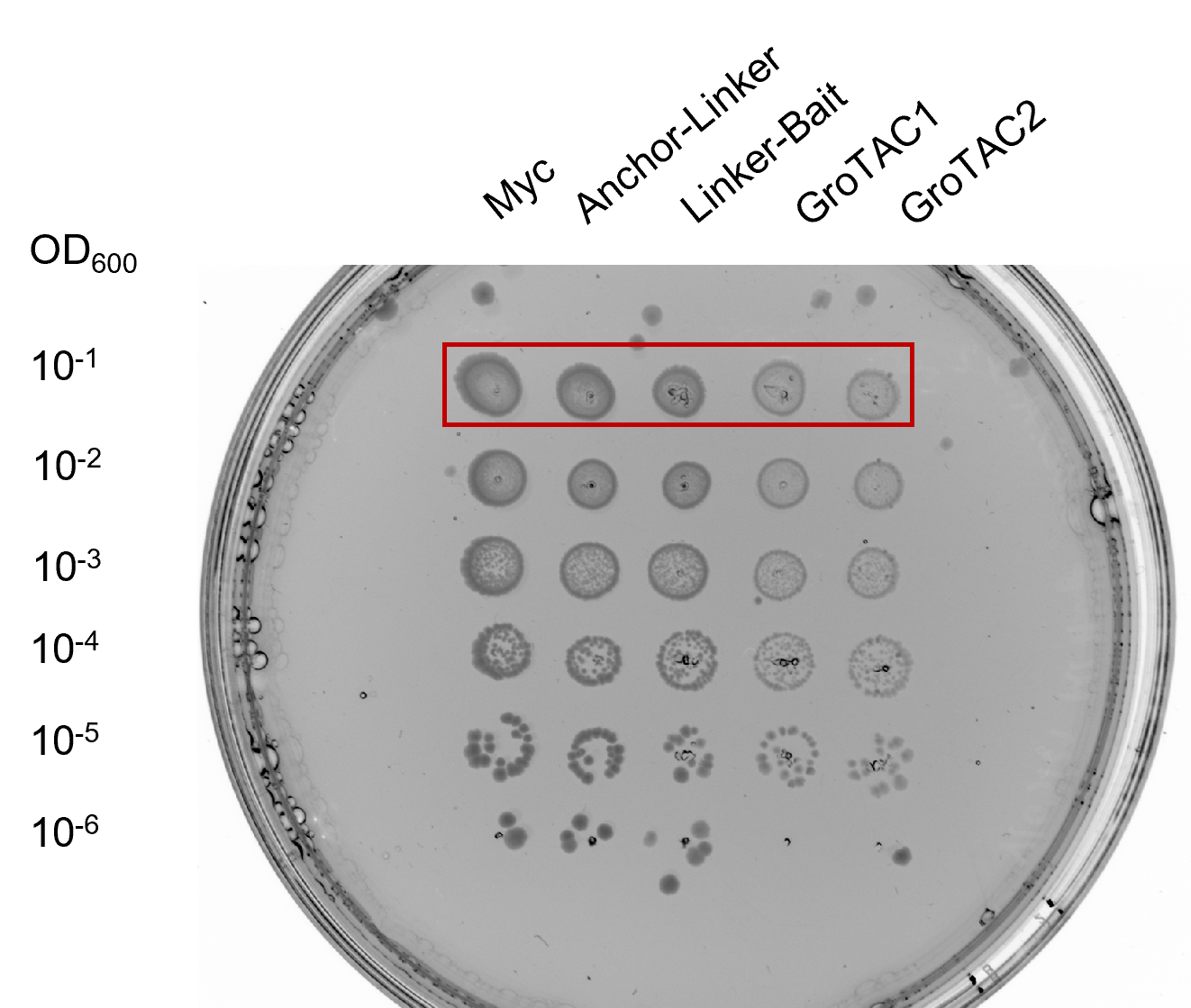

Supplement: Supplementary file 5 — Source data Fig. 2 [file 44319_2025_510_MOESM5_ESM.zip › Fig2/Fig2B/Plate_drop_test_37C.tif]

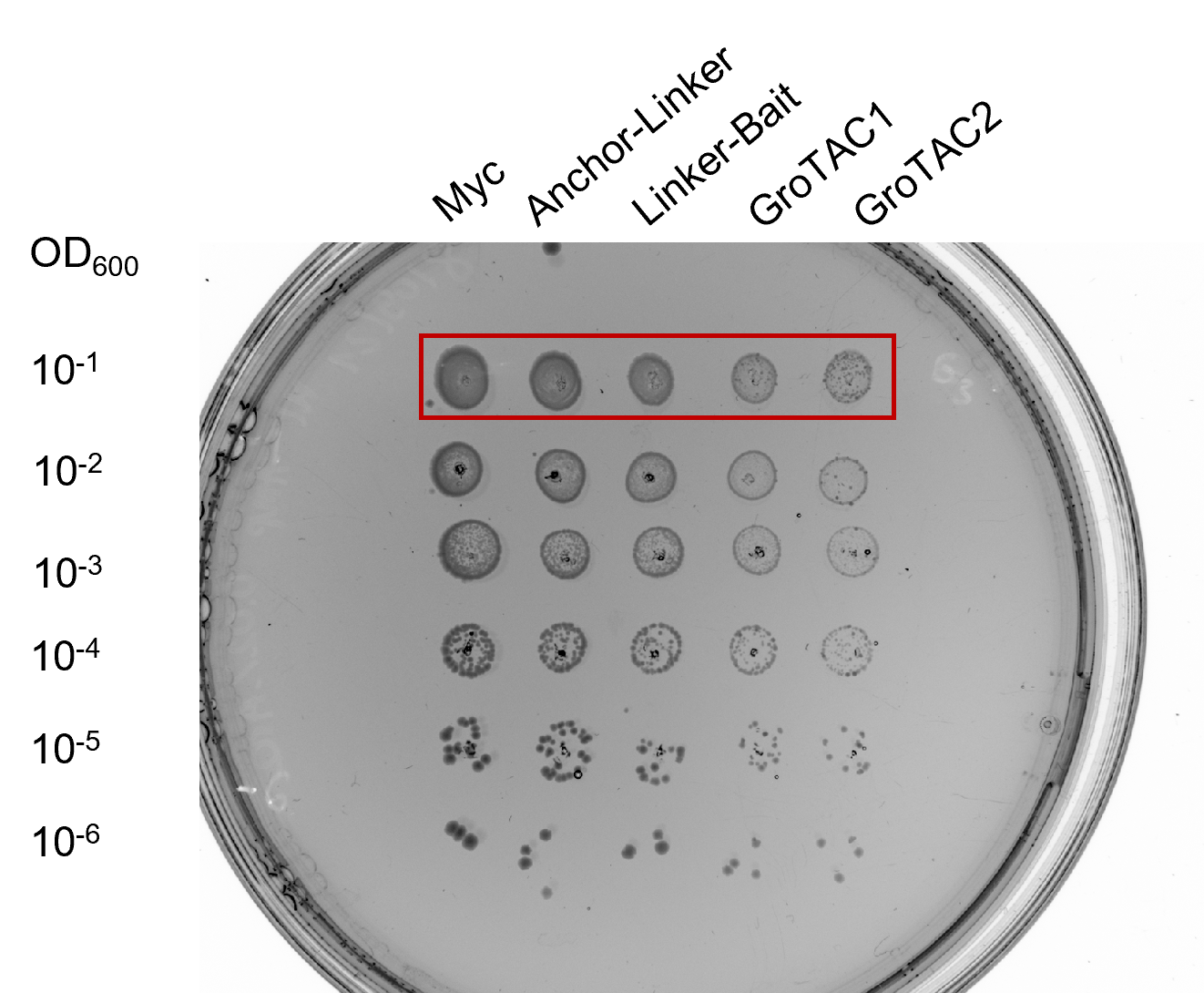

Supplement: Supplementary file 5 — Source data Fig. 2 [file 44319_2025_510_MOESM5_ESM.zip › Fig2/Fig2B/Plate_drop_test_42C.tif]

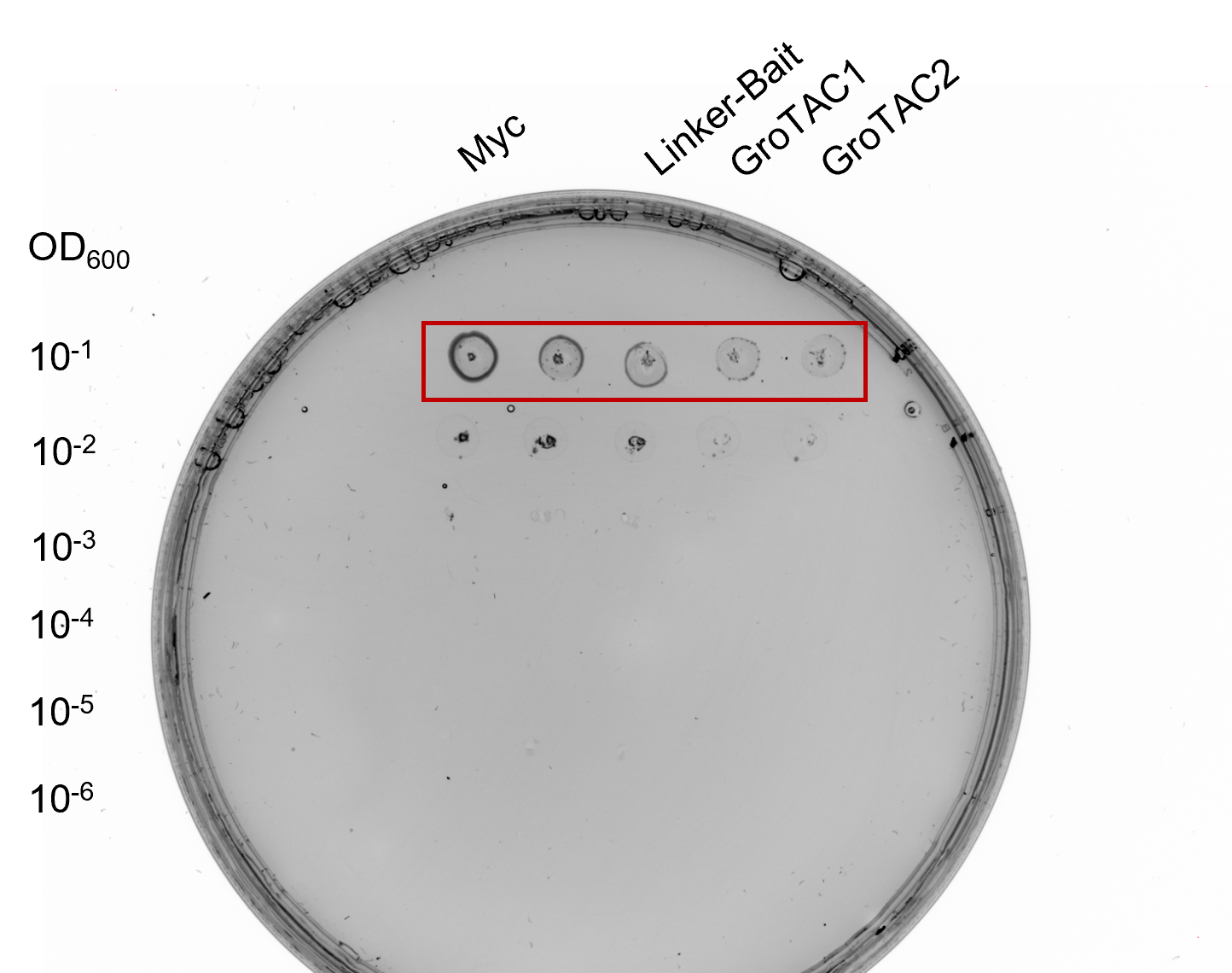

Supplement: Supplementary file 5 — Source data Fig. 2 [file 44319_2025_510_MOESM5_ESM.zip › Fig2/Fig2B/Plate_drop_test_45C.tif]

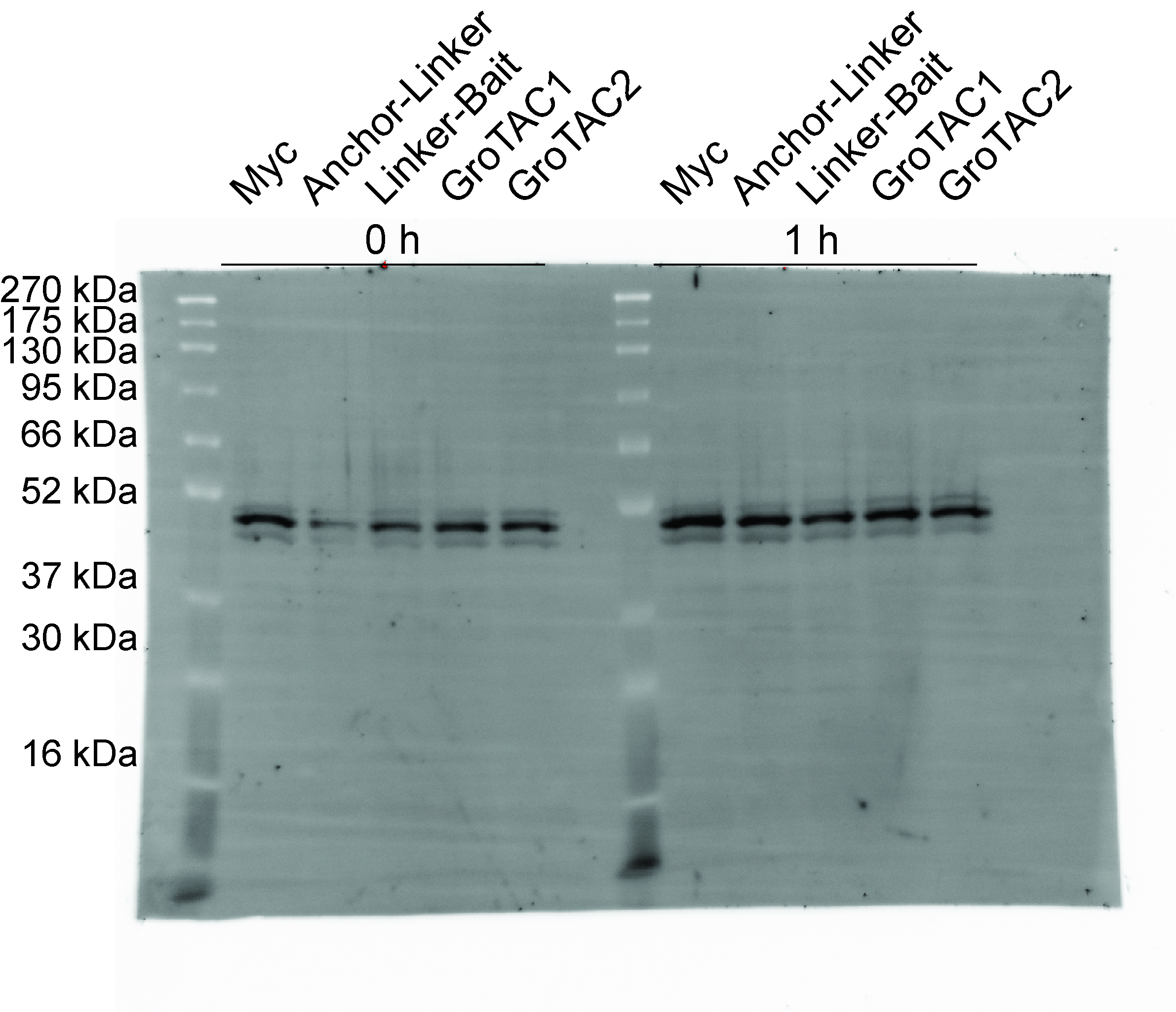

Supplement: Supplementary file 5 — Source data Fig. 2 [file 44319_2025_510_MOESM5_ESM.zip › Fig2/Fig2G/western blot/n1/western_blot_enolase_0h_1h_n1.tif]

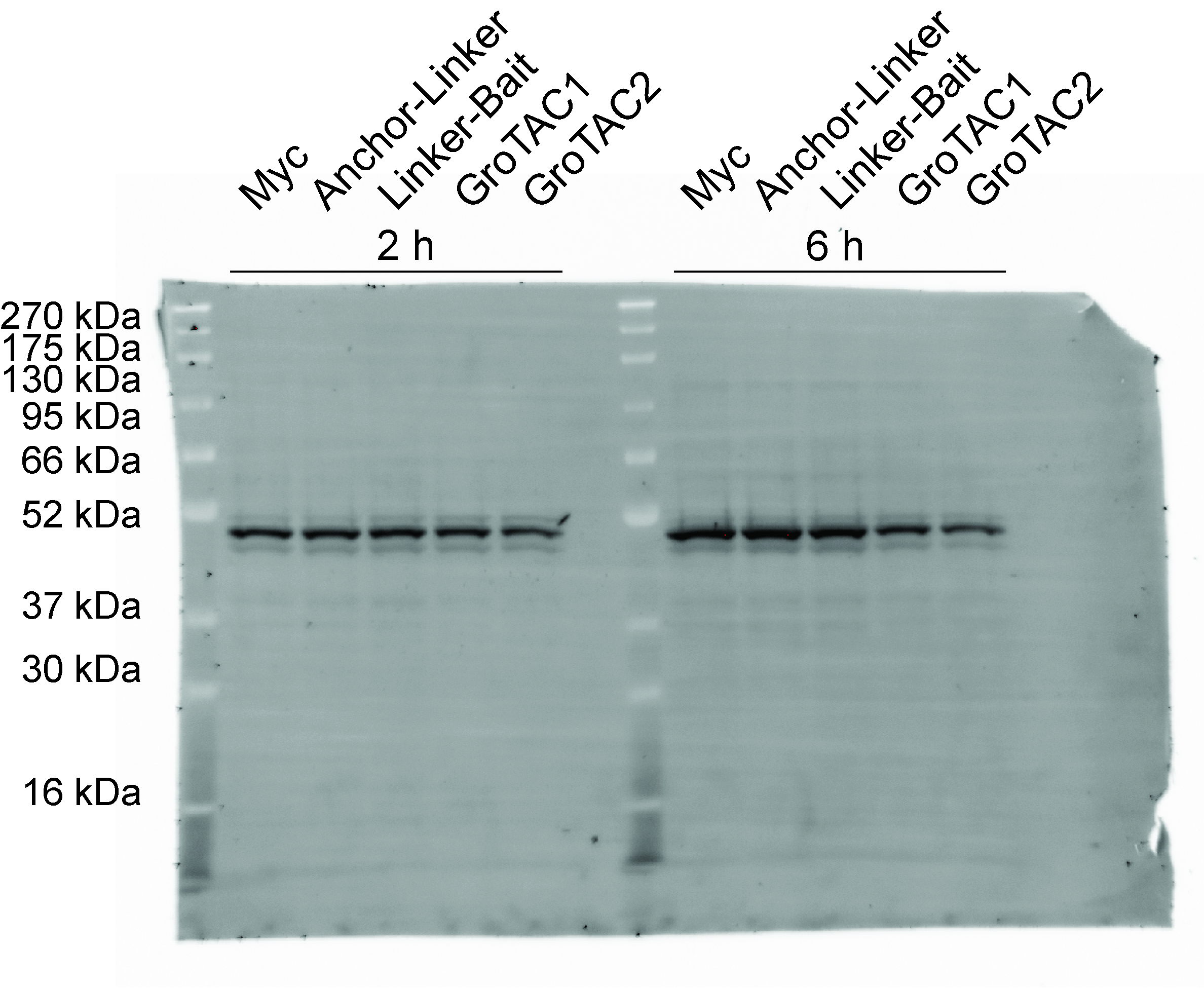

Supplement: Supplementary file 5 — Source data Fig. 2 [file 44319_2025_510_MOESM5_ESM.zip › Fig2/Fig2G/western blot/n1/western_blot_enolase_2h_6h_n1.tif]

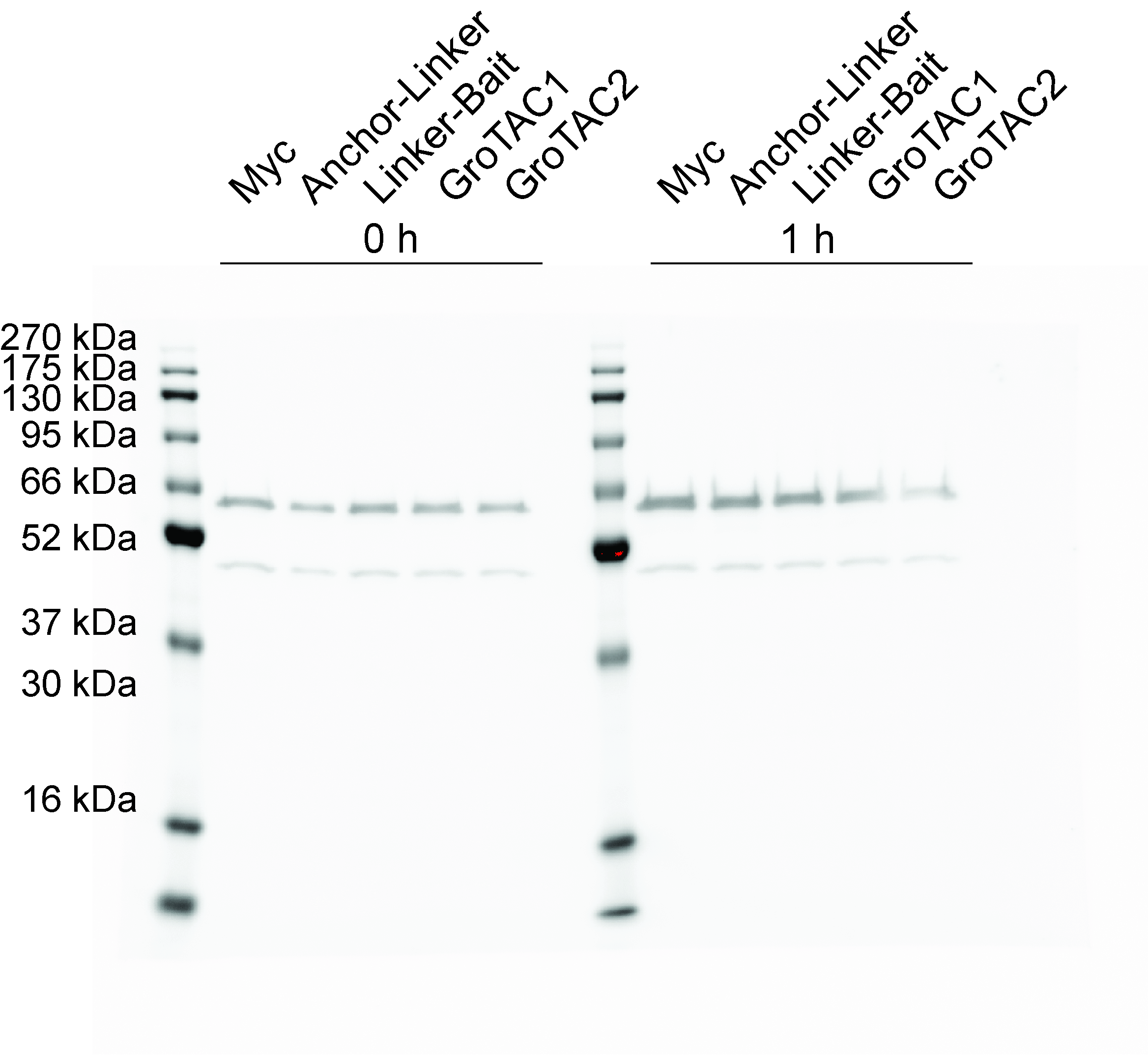

Supplement: Supplementary file 5 — Source data Fig. 2 [file 44319_2025_510_MOESM5_ESM.zip › Fig2/Fig2G/western blot/n1/western_blot_GroEL_0h_1h_n1.tif]

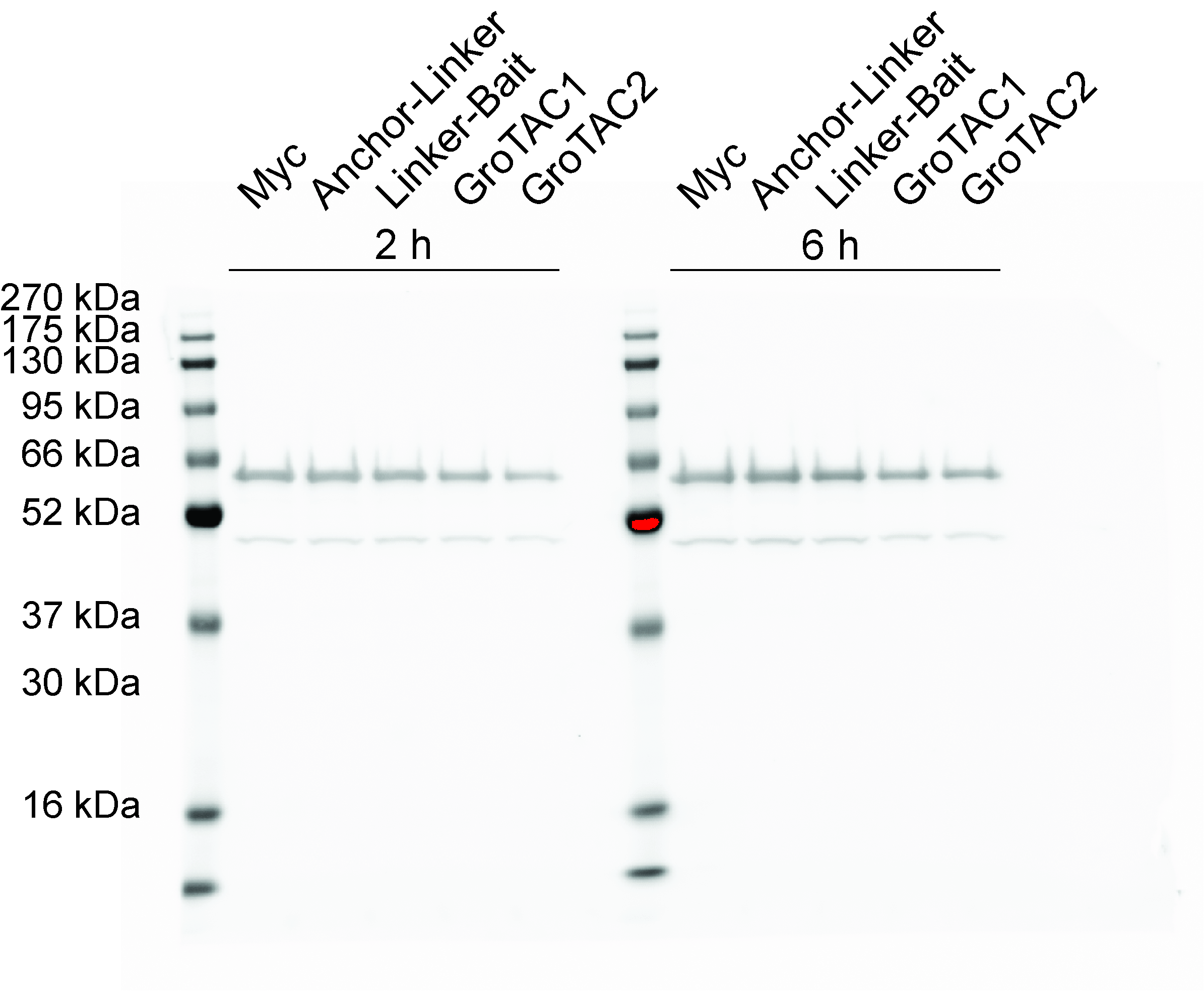

Supplement: Supplementary file 5 — Source data Fig. 2 [file 44319_2025_510_MOESM5_ESM.zip › Fig2/Fig2G/western blot/n1/western_blot_GroEL_2h_6h_n1.tif]

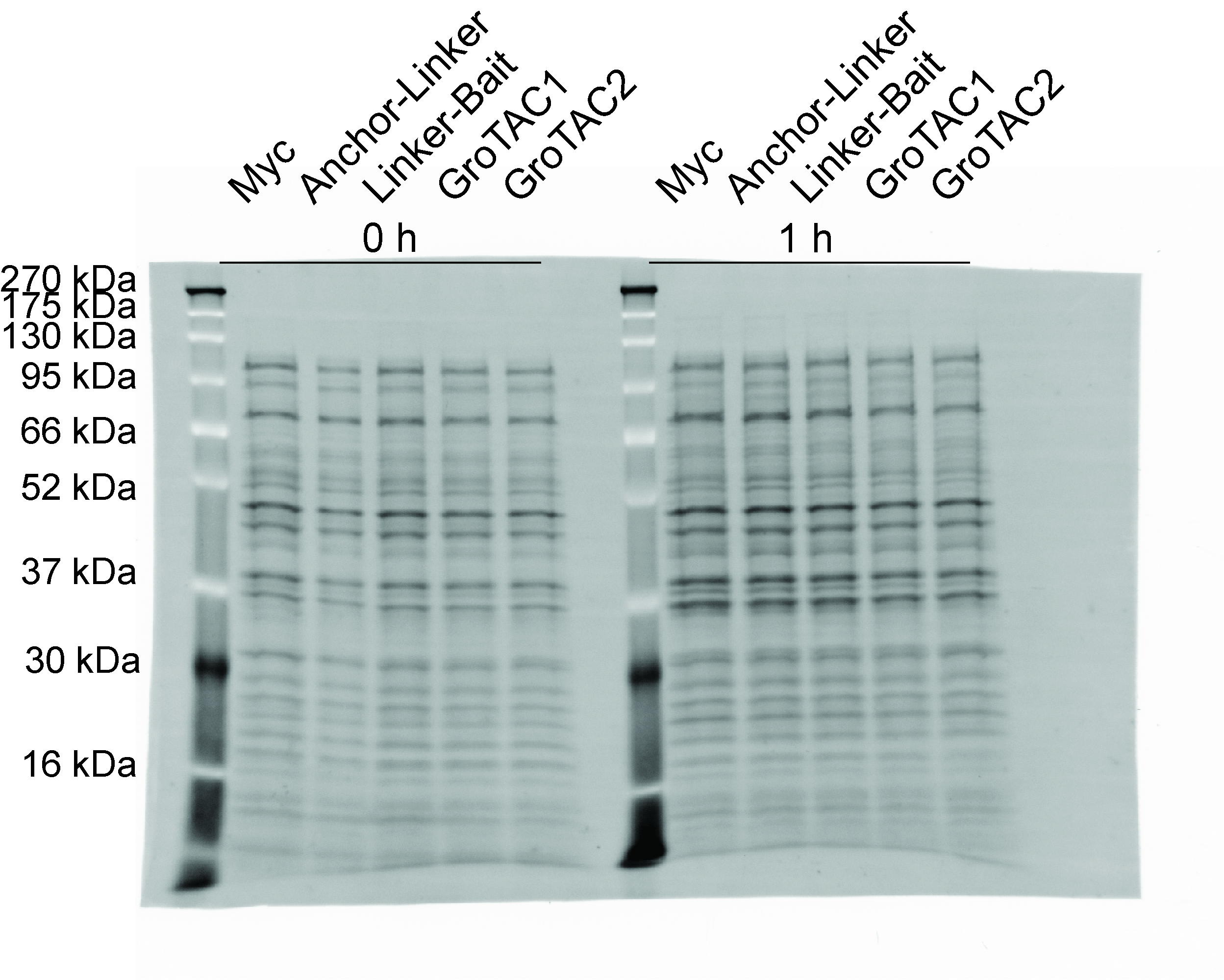

Supplement: Supplementary file 5 — Source data Fig. 2 [file 44319_2025_510_MOESM5_ESM.zip › Fig2/Fig2G/western blot/n1/western_blot_stainfree_0h_1h_n1.tif]

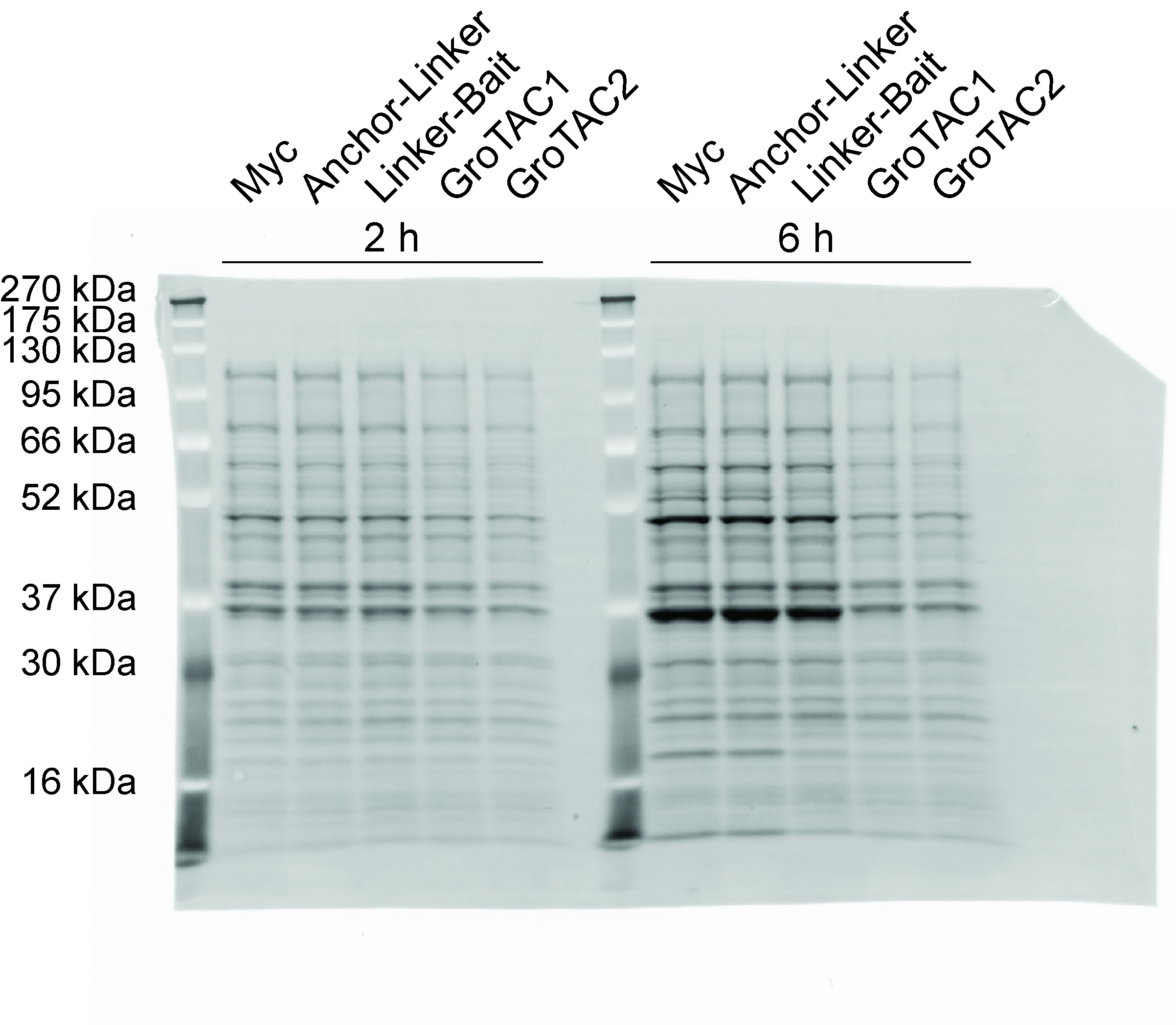

Supplement: Supplementary file 5 — Source data Fig. 2 [file 44319_2025_510_MOESM5_ESM.zip › Fig2/Fig2G/western blot/n1/western_blot_stainfree_2h_6h_n1.tif]

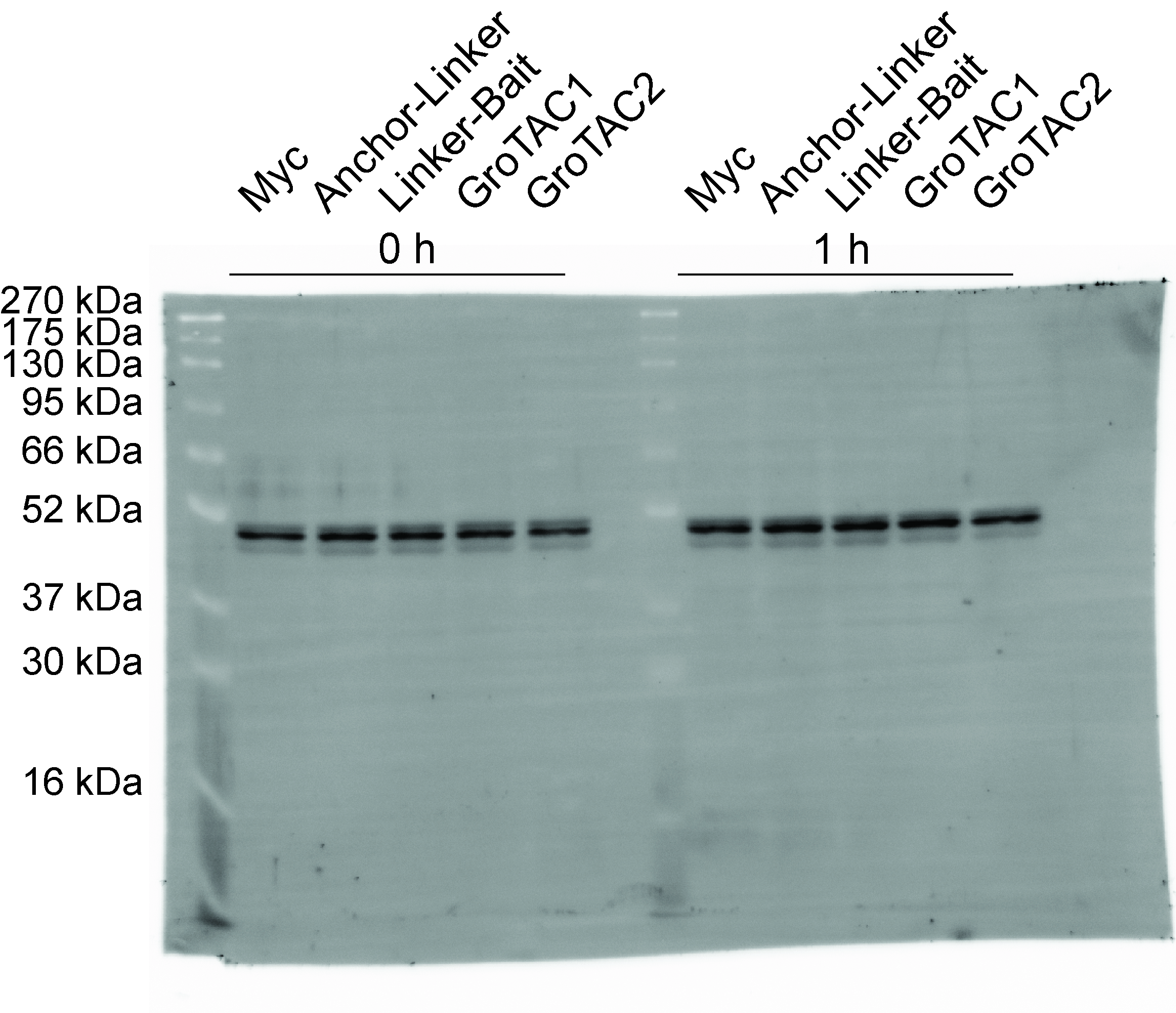

Supplement: Supplementary file 5 — Source data Fig. 2 [file 44319_2025_510_MOESM5_ESM.zip › Fig2/Fig2G/western blot/n2/western_blot_enolase_0h_1h_n2.tif]

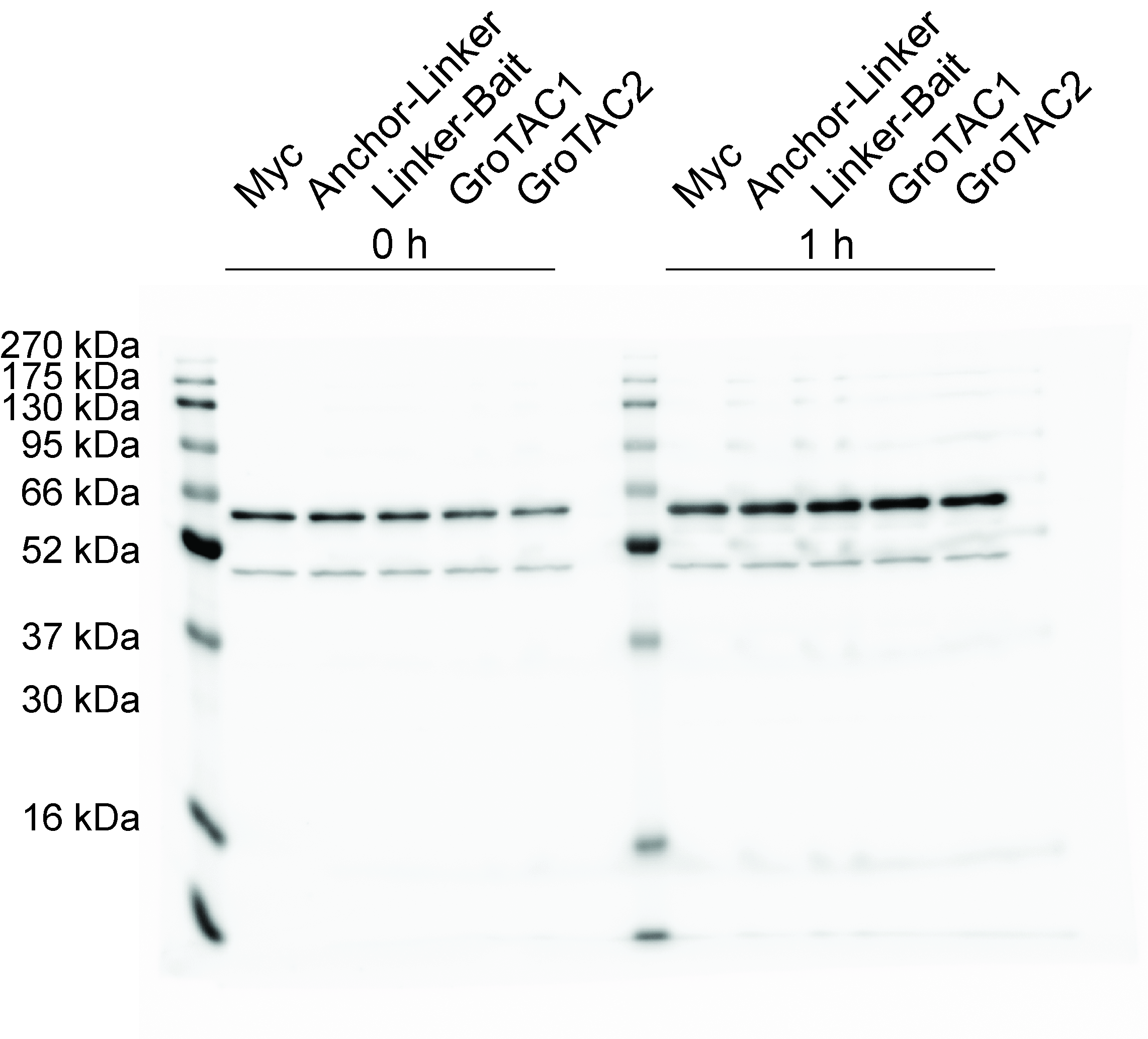

Supplement: Supplementary file 5 — Source data Fig. 2 [file 44319_2025_510_MOESM5_ESM.zip › Fig2/Fig2G/western blot/n2/western_blot_GroEL_0h_1h_n2.tif]

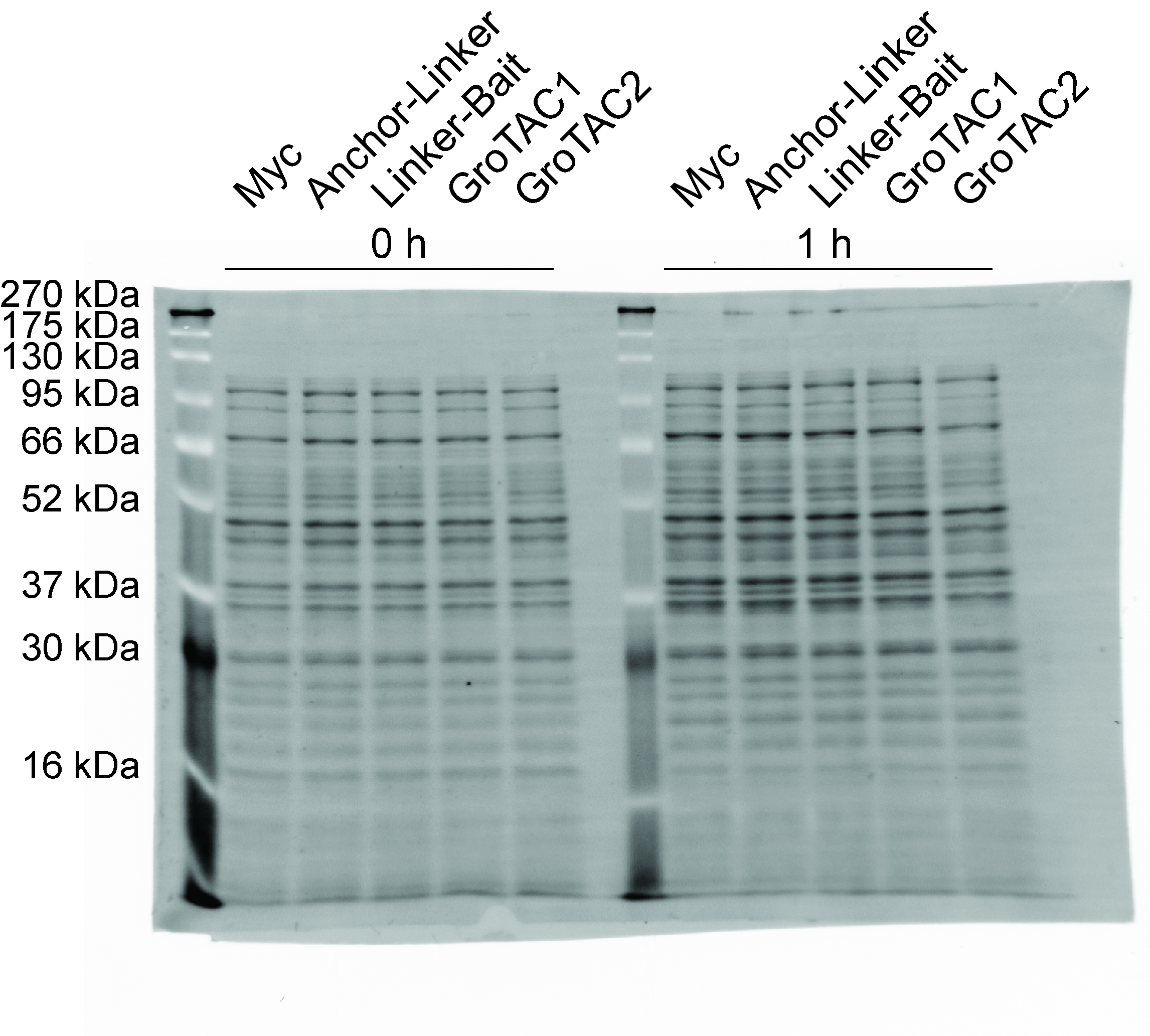

Supplement: Supplementary file 5 — Source data Fig. 2 [file 44319_2025_510_MOESM5_ESM.zip › Fig2/Fig2G/western blot/n2/western_blot_stainfree_0h_1h_n2.tif]

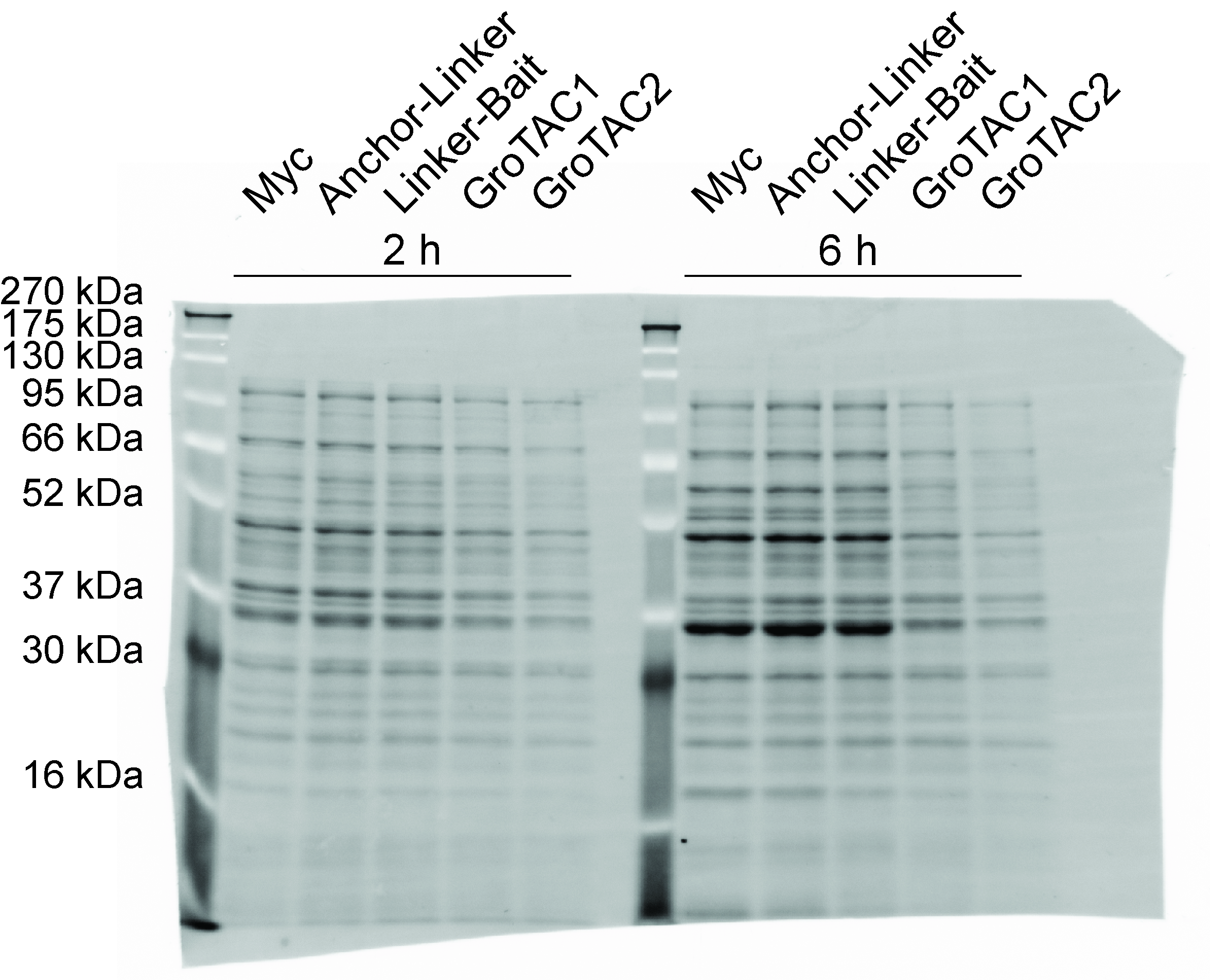

Supplement: Supplementary file 5 — Source data Fig. 2 [file 44319_2025_510_MOESM5_ESM.zip › Fig2/Fig2G/western blot/n2/western_blot_stainfree_2h_6h_n2.tif]

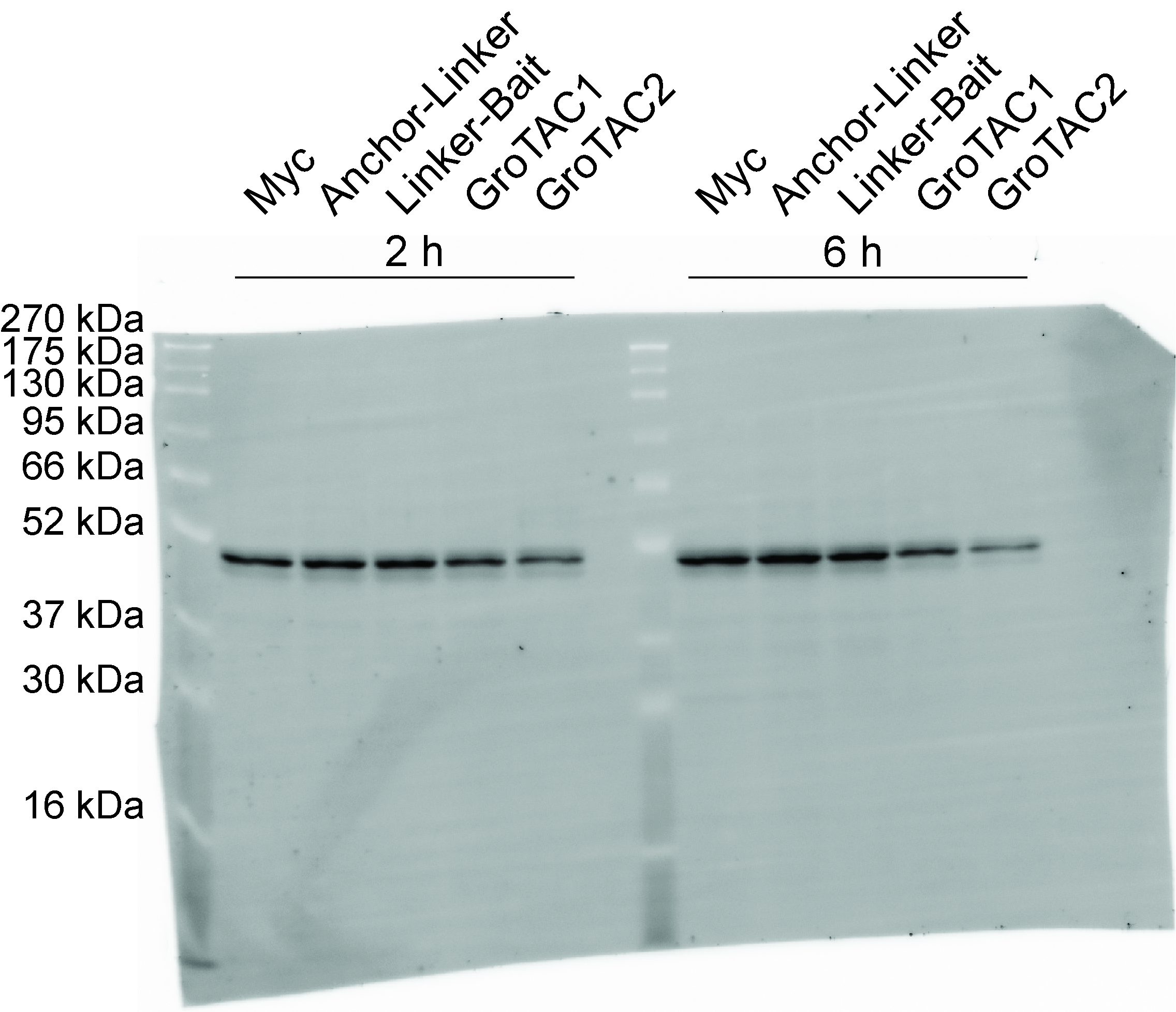

Supplement: Supplementary file 5 — Source data Fig. 2 [file 44319_2025_510_MOESM5_ESM.zip › Fig2/Fig2G/western blot/n2/western_enolase_2h_6h_n2.tif]

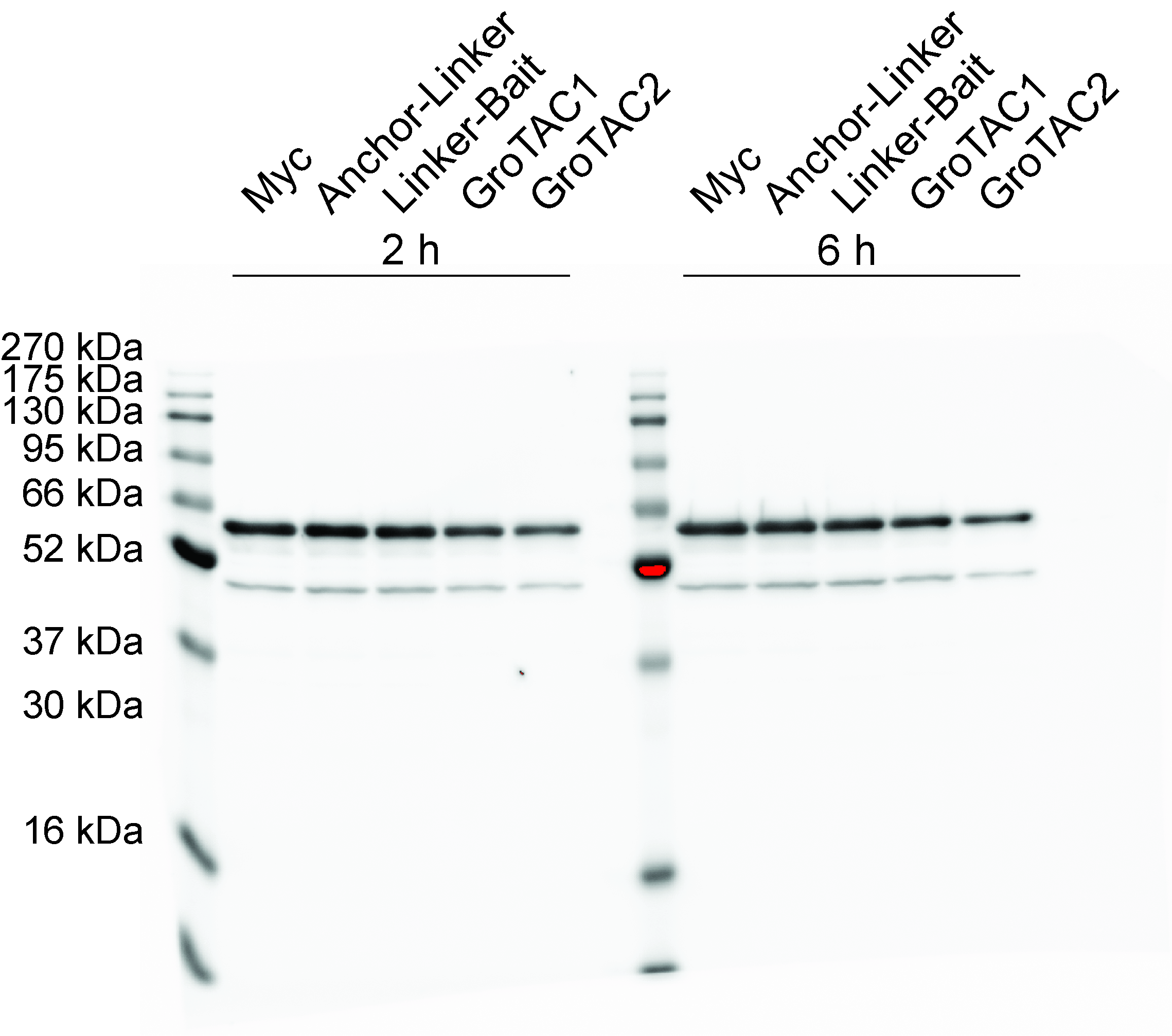

Supplement: Supplementary file 5 — Source data Fig. 2 [file 44319_2025_510_MOESM5_ESM.zip › Fig2/Fig2G/western blot/n2/western_GroEL_2h_6h_n2.tif]

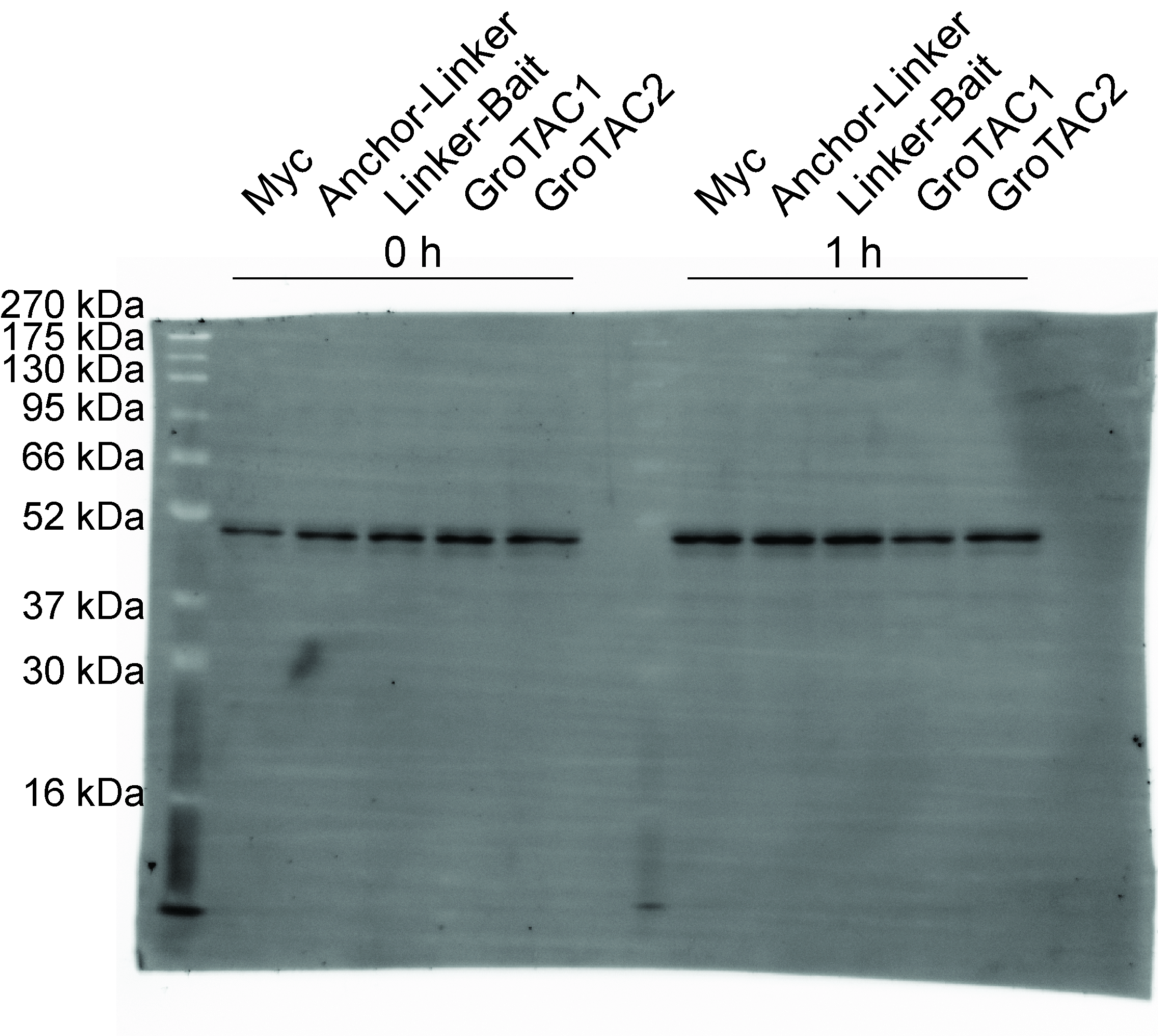

Supplement: Supplementary file 5 — Source data Fig. 2 [file 44319_2025_510_MOESM5_ESM.zip › Fig2/Fig2G/western blot/n3/western_blot_enolase_0h_1h_n3.tif]

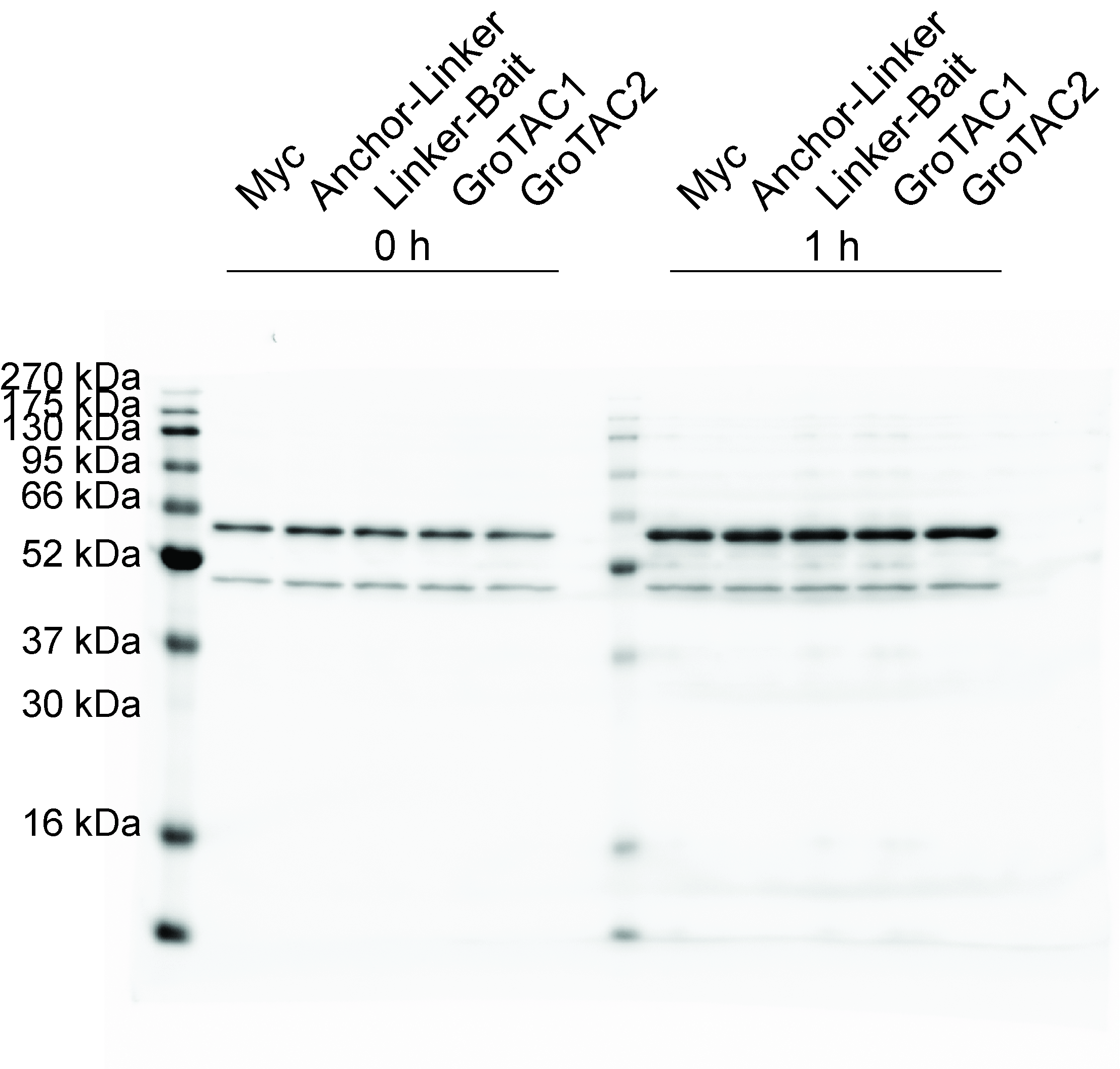

Supplement: Supplementary file 5 — Source data Fig. 2 [file 44319_2025_510_MOESM5_ESM.zip › Fig2/Fig2G/western blot/n3/western_blot_GroEL_0h_1h_n3.tif]

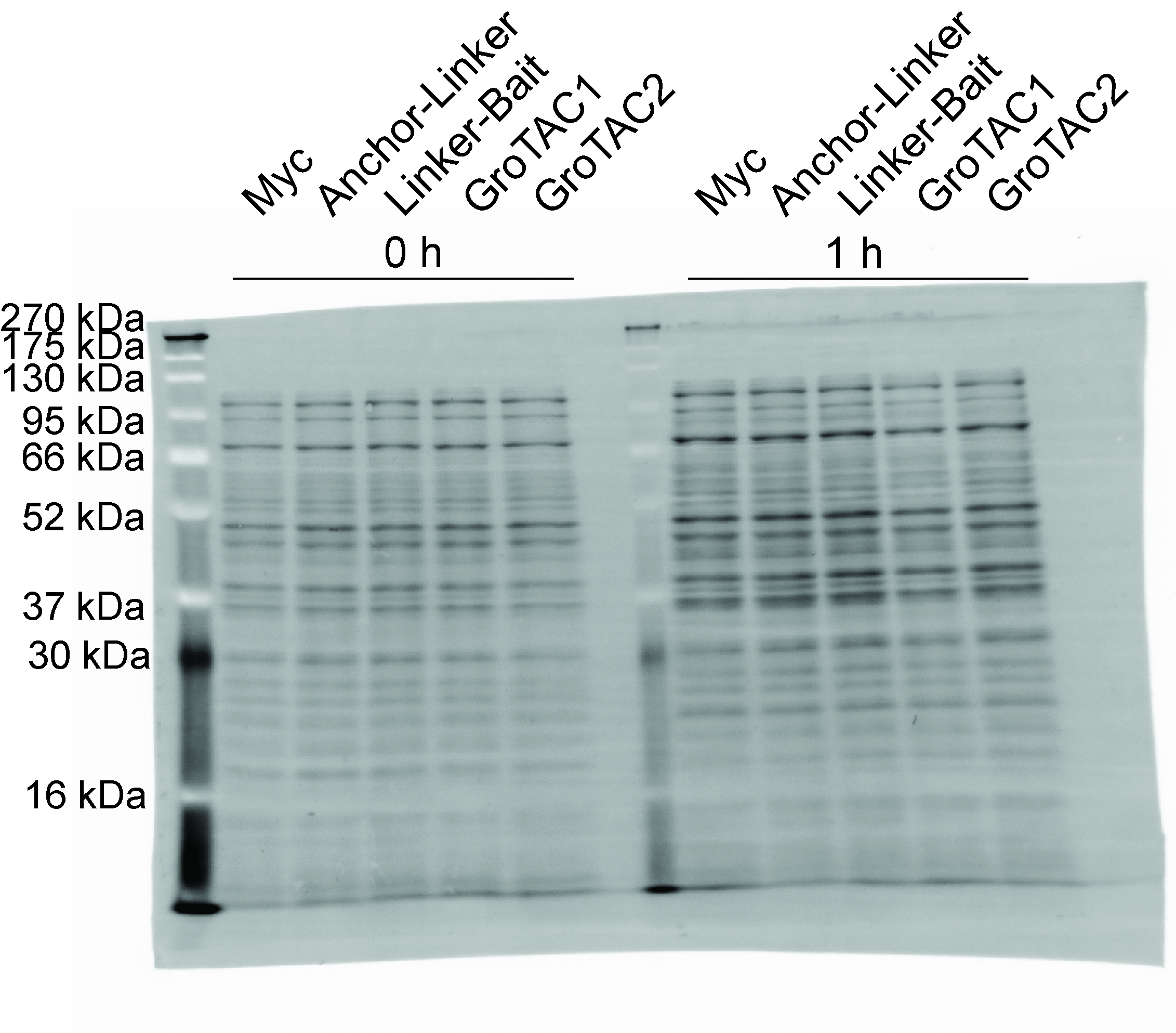

Supplement: Supplementary file 5 — Source data Fig. 2 [file 44319_2025_510_MOESM5_ESM.zip › Fig2/Fig2G/western blot/n3/western_blot_stainfree_0h_1h_n3.tif]

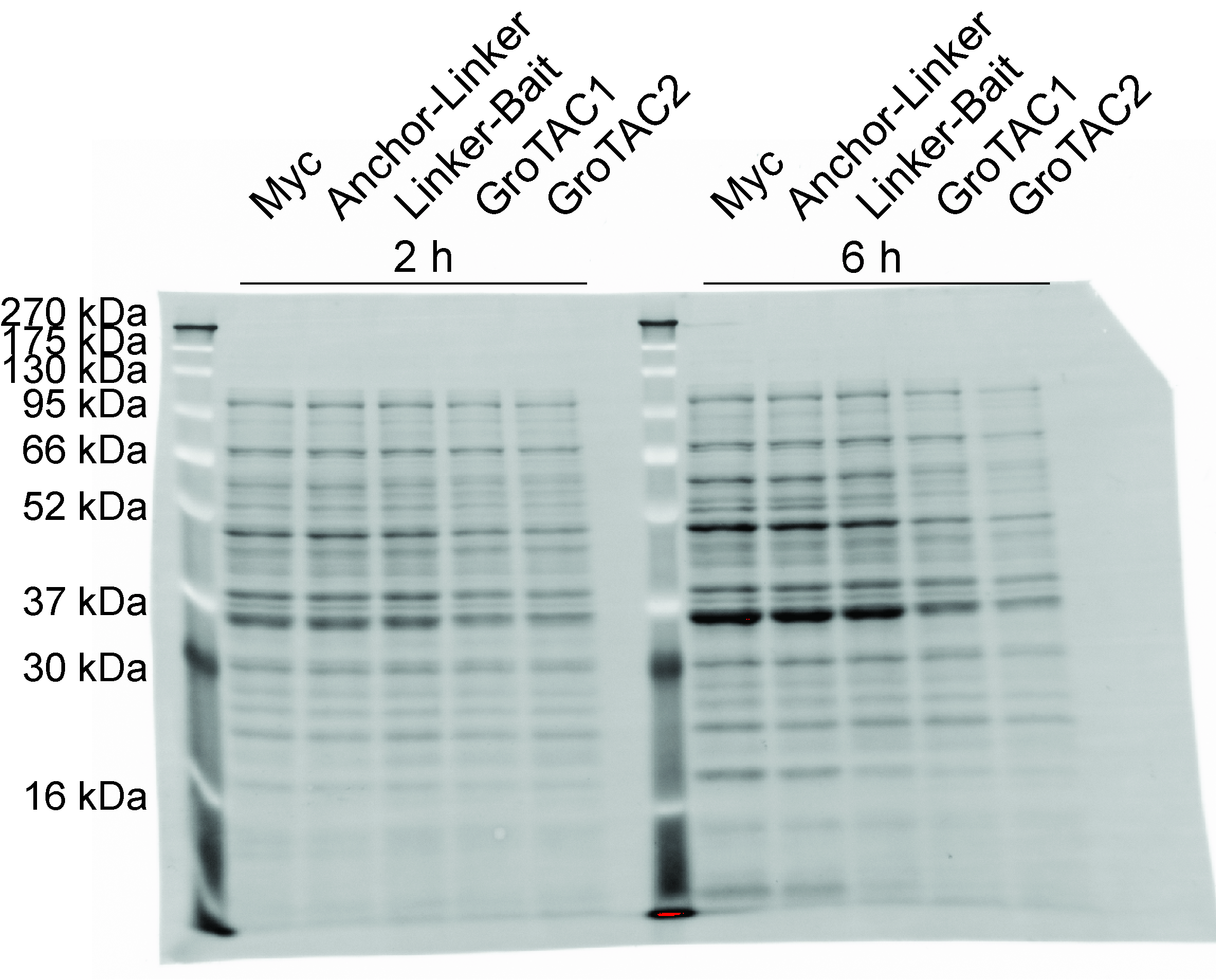

Supplement: Supplementary file 5 — Source data Fig. 2 [file 44319_2025_510_MOESM5_ESM.zip › Fig2/Fig2G/western blot/n3/western_blot_stainfree_2h_6h_n3.tif]

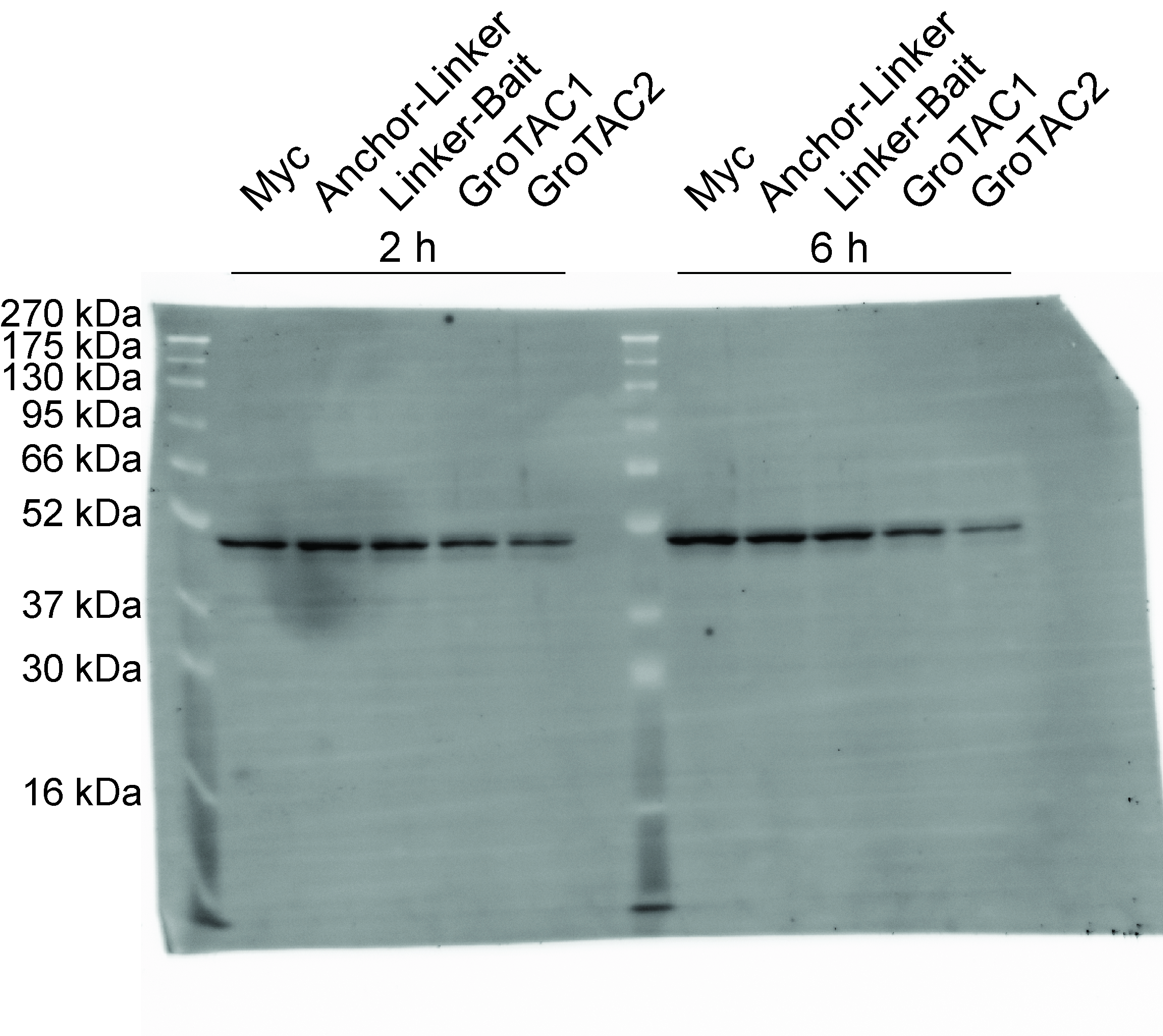

Supplement: Supplementary file 5 — Source data Fig. 2 [file 44319_2025_510_MOESM5_ESM.zip › Fig2/Fig2G/western blot/n3/western_enolase_2h_6h_n3.tif]

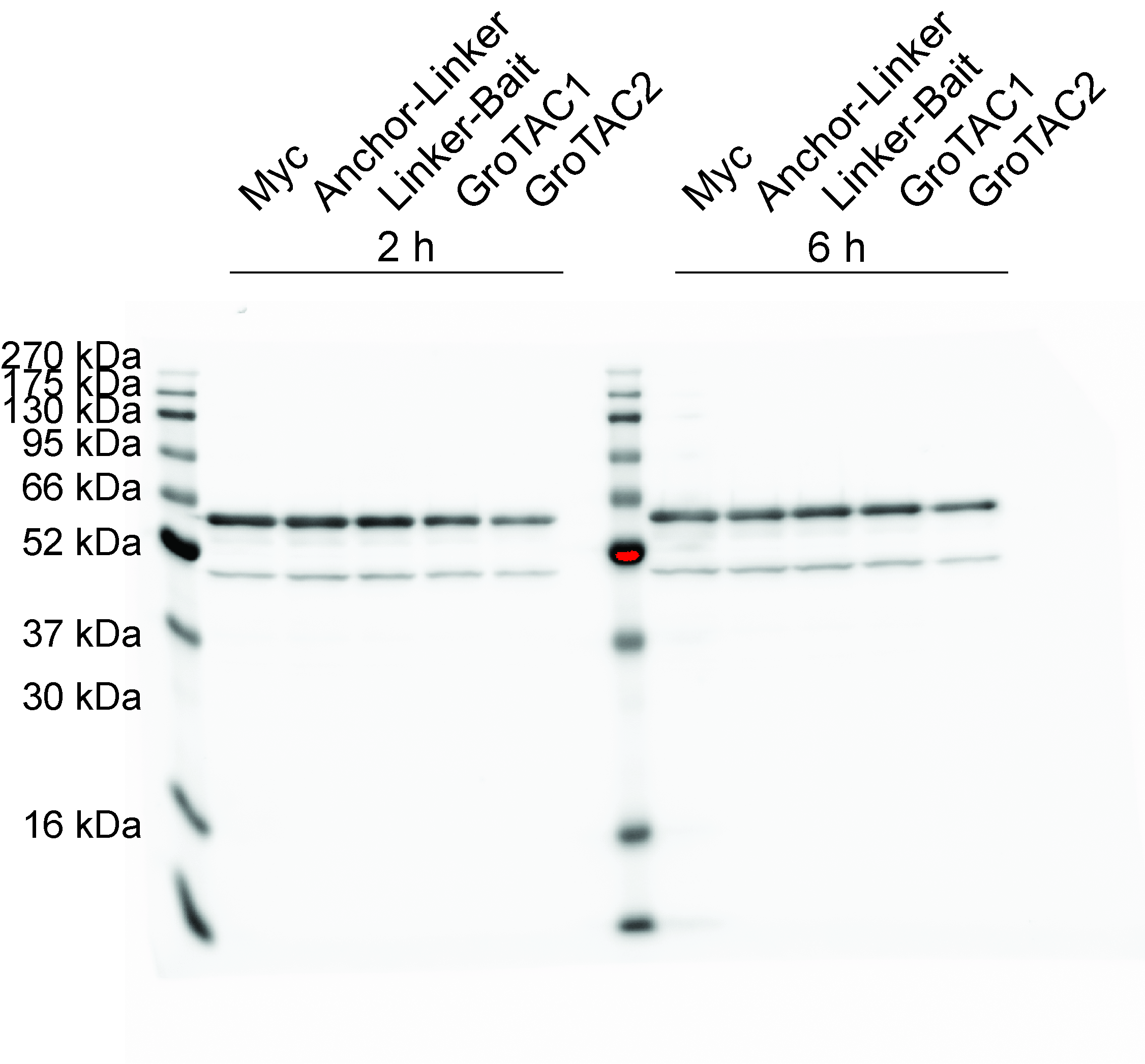

Supplement: Supplementary file 5 — Source data Fig. 2 [file 44319_2025_510_MOESM5_ESM.zip › Fig2/Fig2G/western blot/n3/western_GroEL_2h_6h_n3.tif]

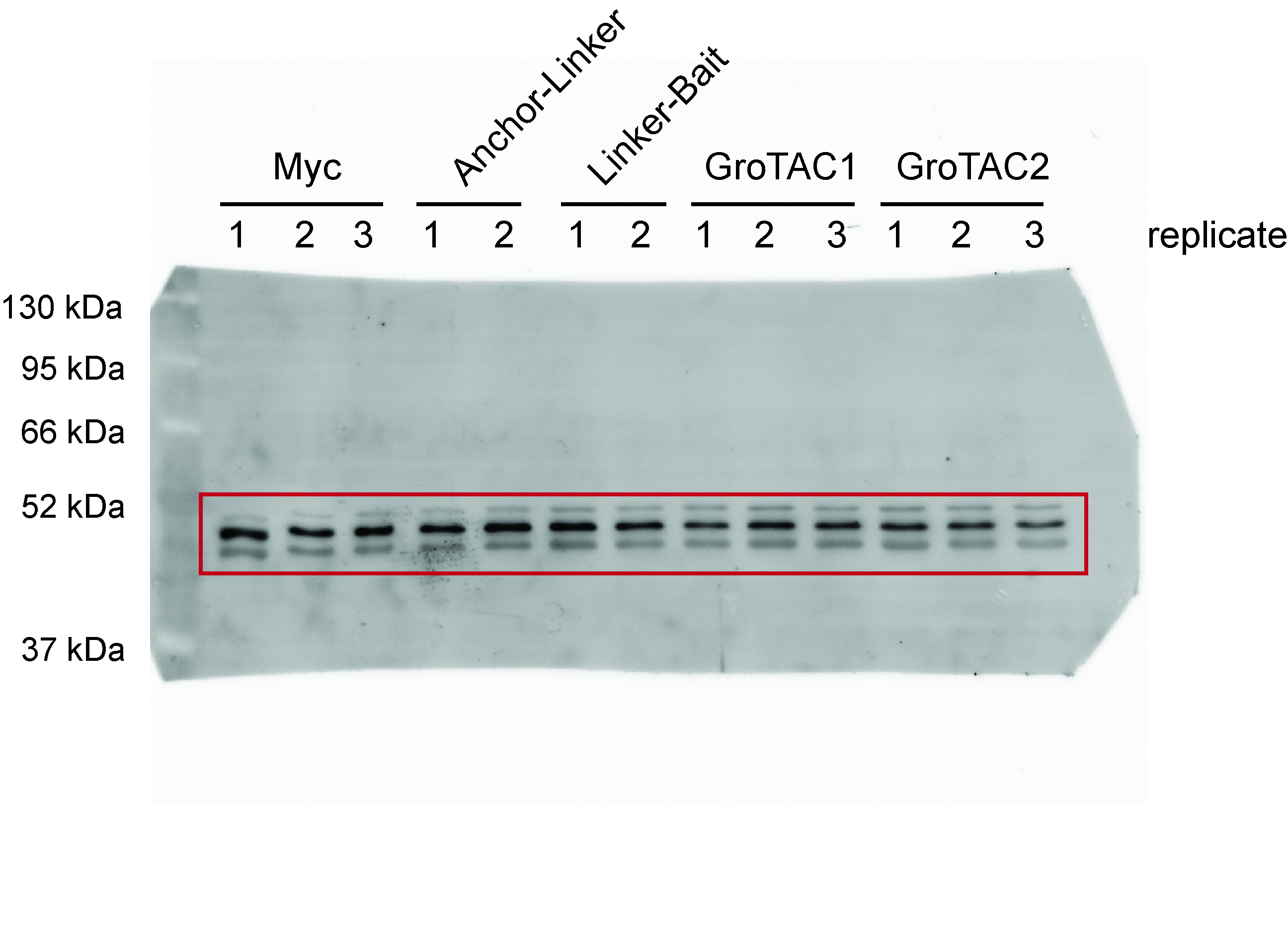

Supplement: Supplementary file 5 — Source data Fig. 2 [file 44319_2025_510_MOESM5_ESM.zip › Fig2/Fig2H/western_blot_enolase.tif]

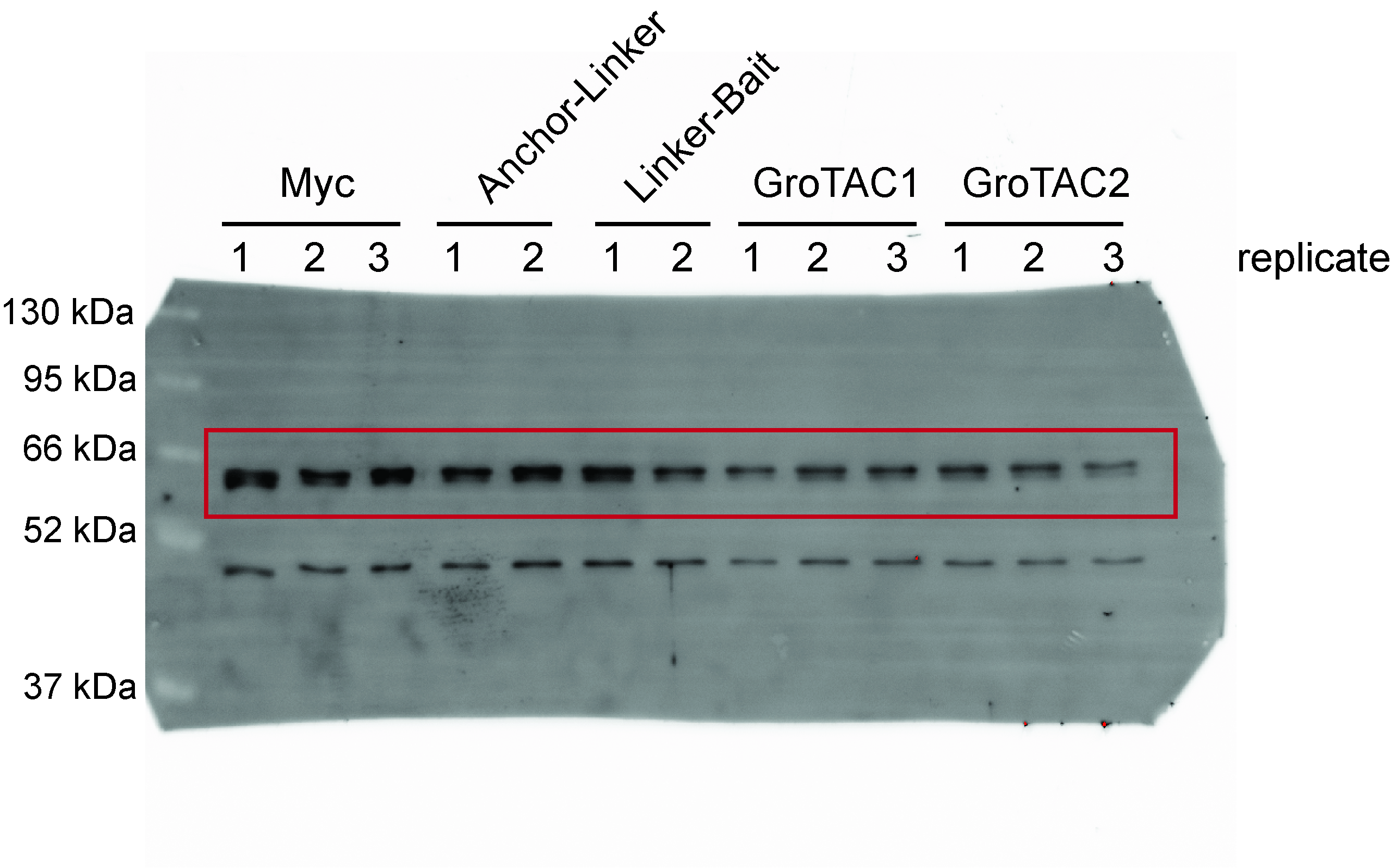

Supplement: Supplementary file 5 — Source data Fig. 2 [file 44319_2025_510_MOESM5_ESM.zip › Fig2/Fig2H/western_blot_GroEL.tif]

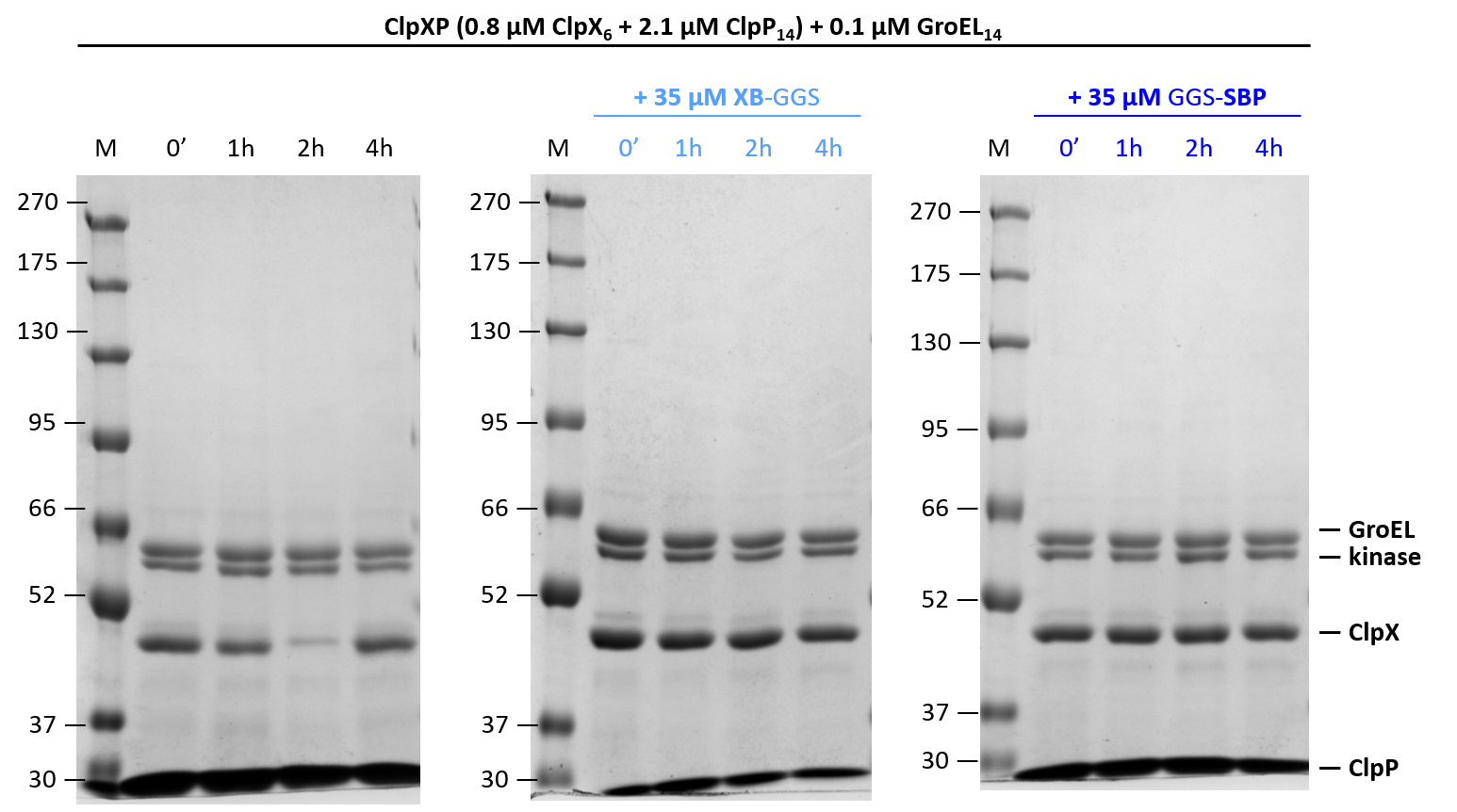

Supplement: Supplementary file 7 — Source data Fig. 4 [file 44319_2025_510_MOESM7_ESM.zip › Fig4/Fig4F/Controls/n1/In_vitro_degradation_control_gels_n1.png]

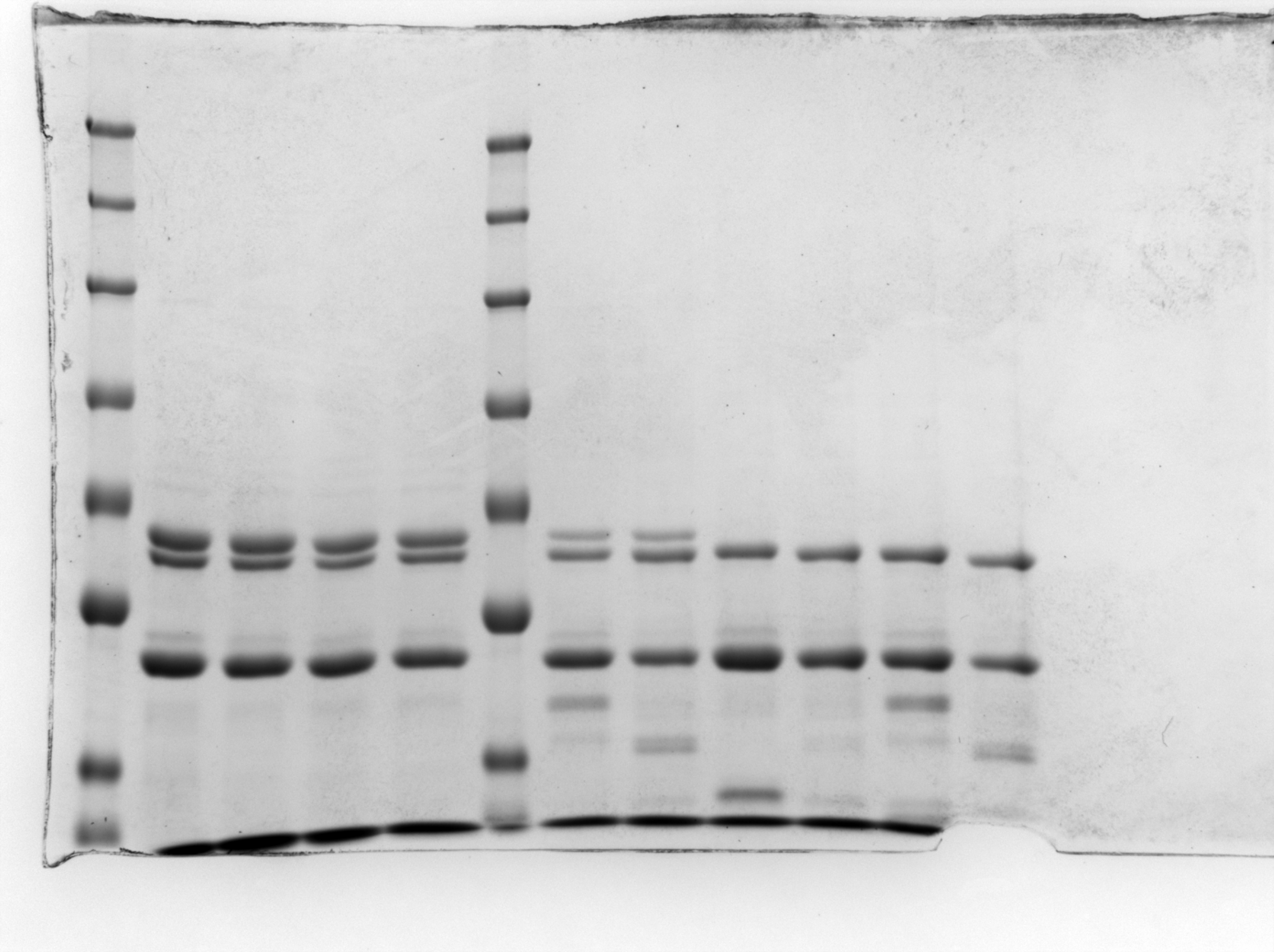

Supplement: Supplementary file 7 — Source data Fig. 4 [file 44319_2025_510_MOESM7_ESM.zip › Fig4/Fig4F/Controls/n1/In_vitro_degradation_control_gel_Anchor-Linker_n1.tif]

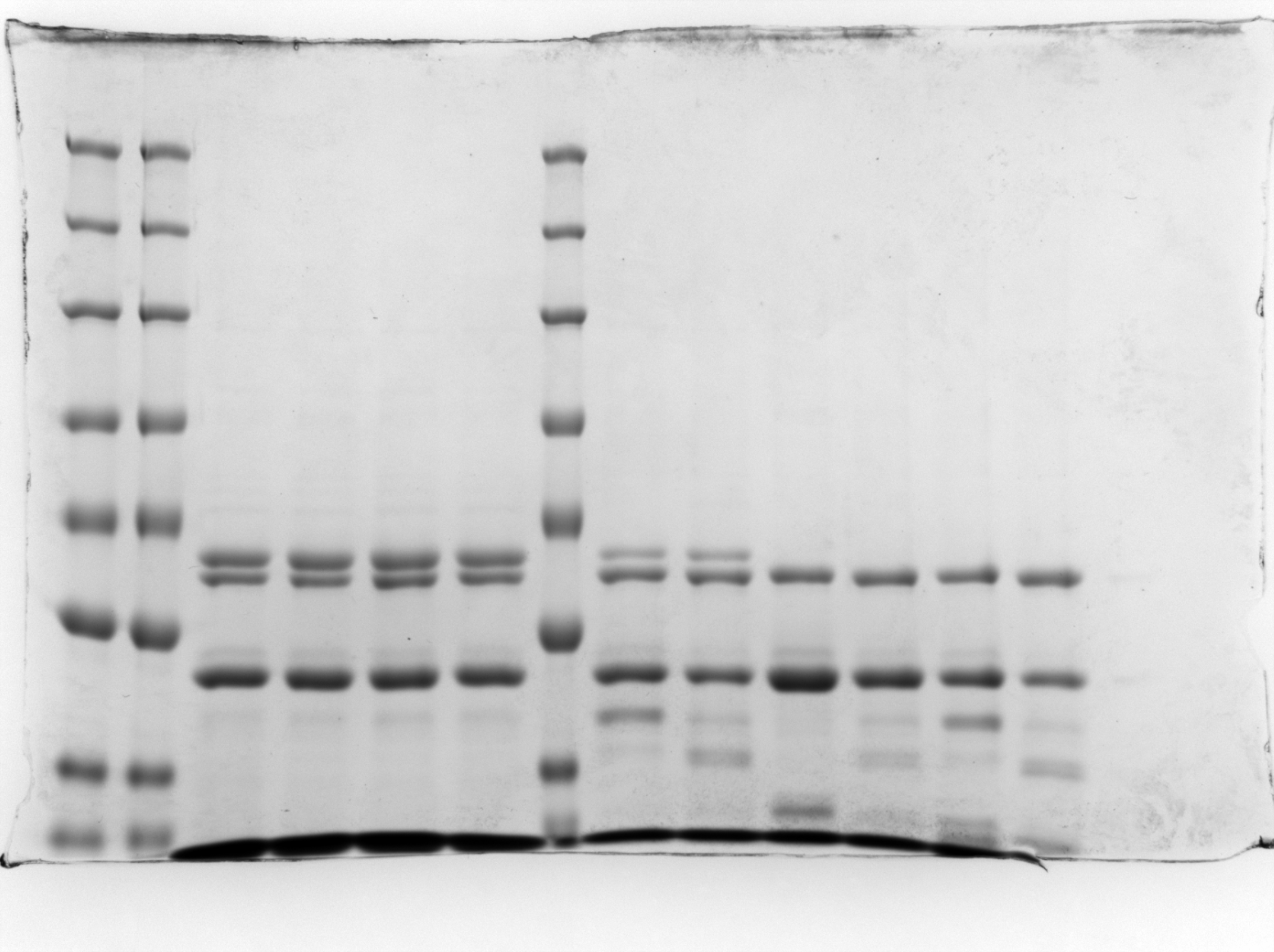

Supplement: Supplementary file 7 — Source data Fig. 4 [file 44319_2025_510_MOESM7_ESM.zip › Fig4/Fig4F/Controls/n1/In_vitro_degradation_control_gel_Linker-Bait_n1.tif]

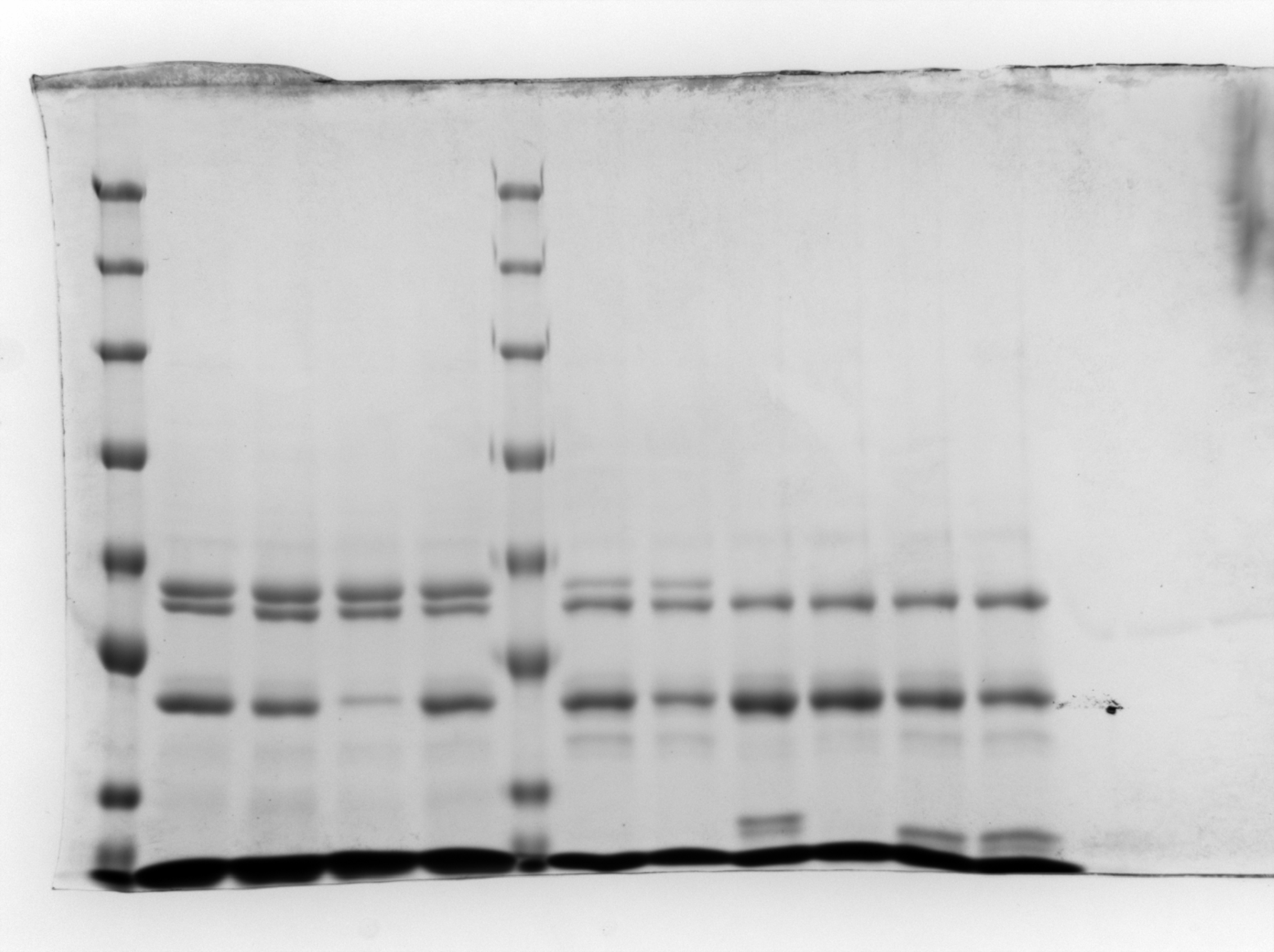

Supplement: Supplementary file 7 — Source data Fig. 4 [file 44319_2025_510_MOESM7_ESM.zip › Fig4/Fig4F/Controls/n1/In_vitro_degradation_control_gel_n1.tif]

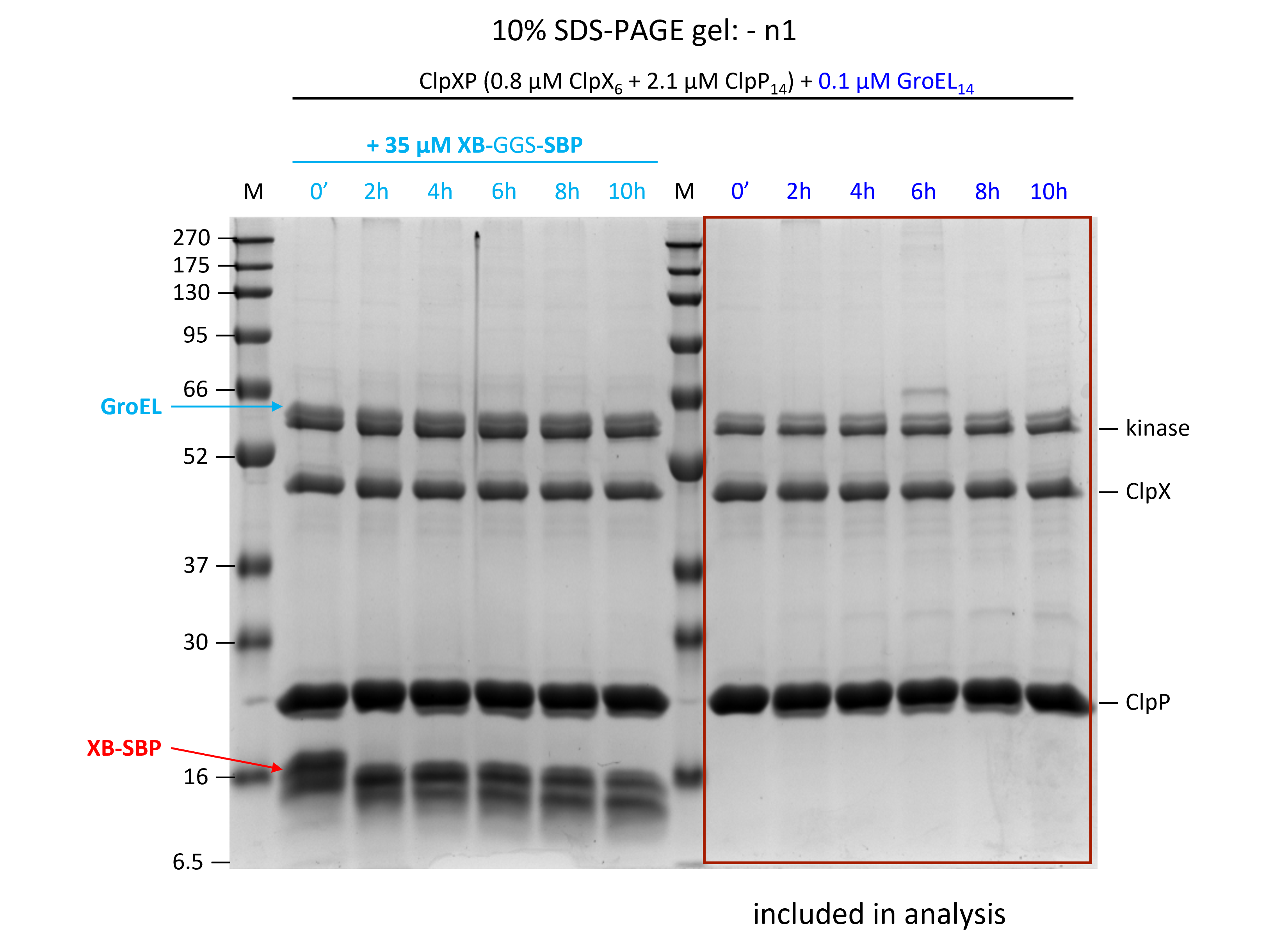

Supplement: Supplementary file 7 — Source data Fig. 4 [file 44319_2025_510_MOESM7_ESM.zip › Fig4/Fig4F/Controls/n2/In_vitro_degradation_control_gels_n2_label.tif]

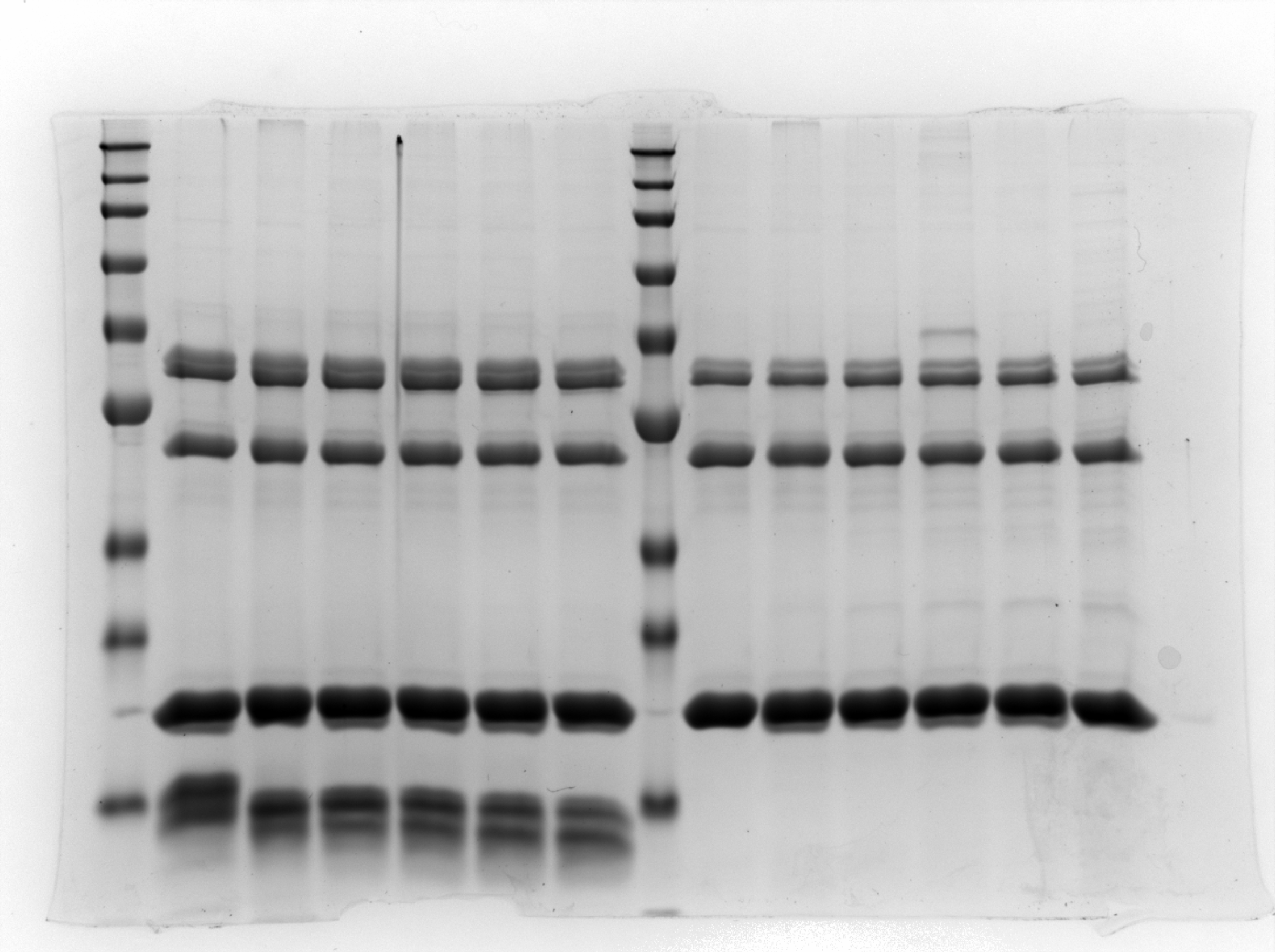

Supplement: Supplementary file 7 — Source data Fig. 4 [file 44319_2025_510_MOESM7_ESM.zip › Fig4/Fig4F/Controls/n2/In_vitro_degradation_control_gel_n2_1.tif]

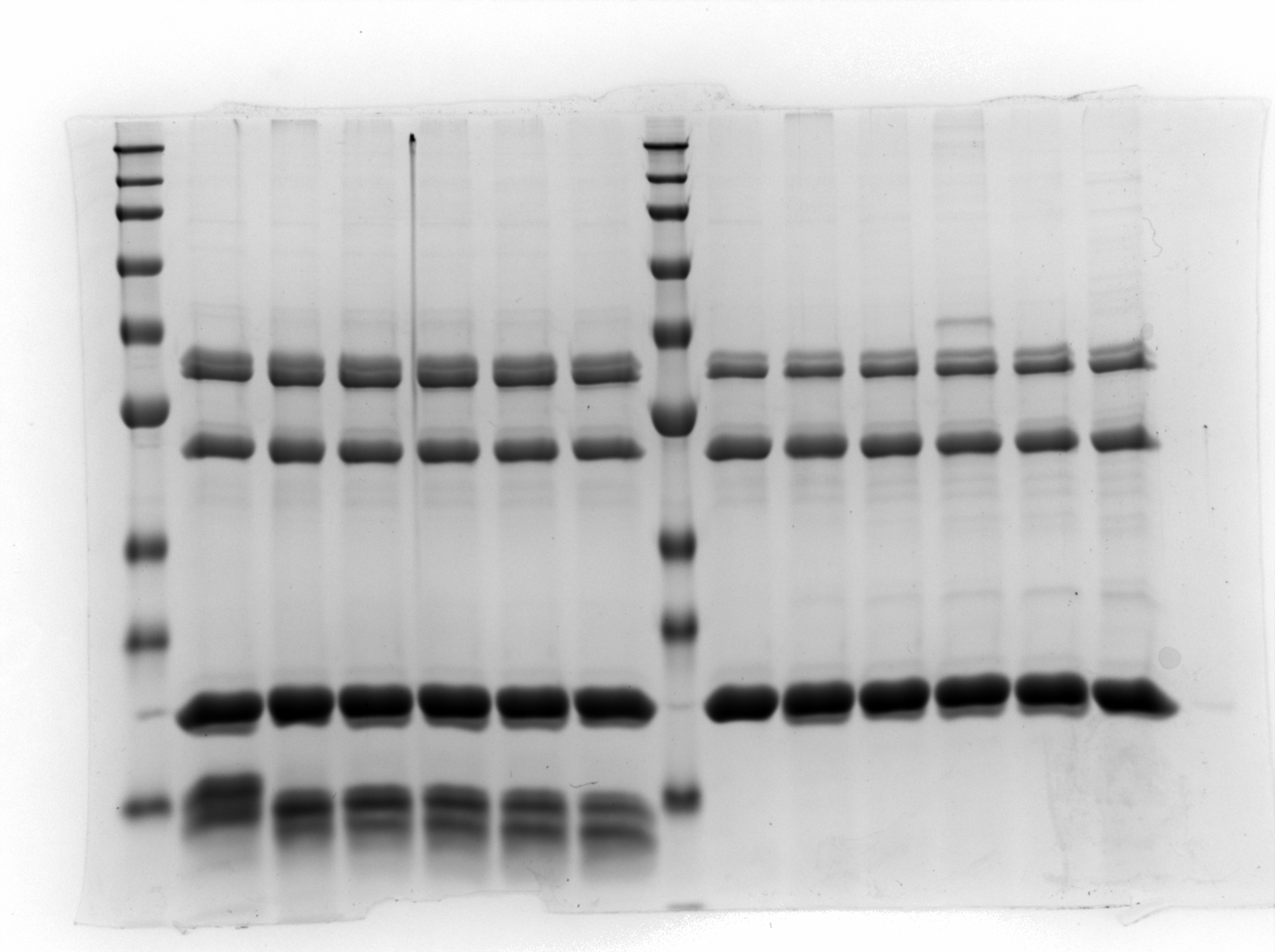

Supplement: Supplementary file 7 — Source data Fig. 4 [file 44319_2025_510_MOESM7_ESM.zip › Fig4/Fig4F/Controls/n2/In_vitro_degradation_control_gel_n2_2.tif]

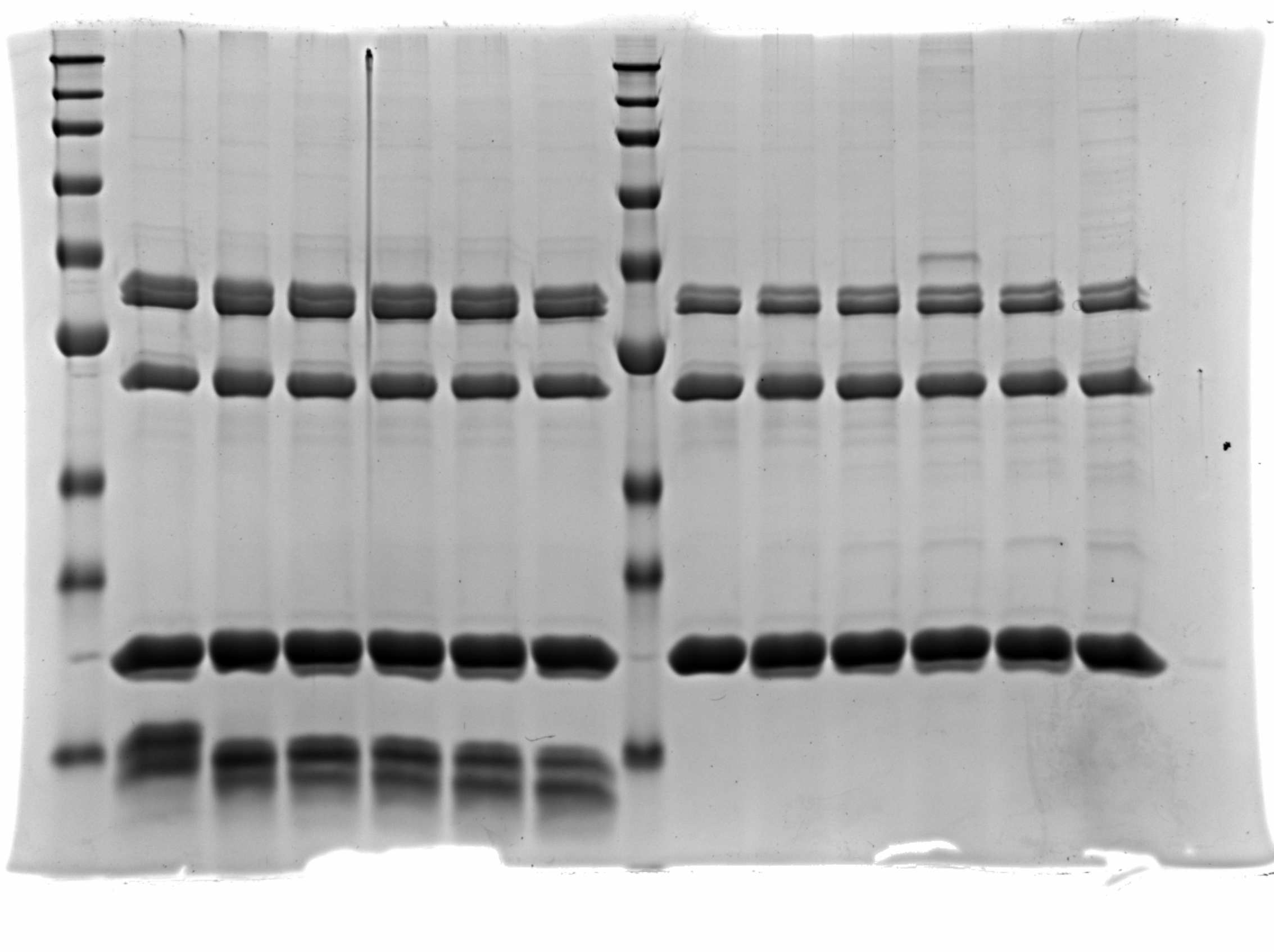

Supplement: Supplementary file 7 — Source data Fig. 4 [file 44319_2025_510_MOESM7_ESM.zip › Fig4/Fig4F/Controls/n2/In_vitro_degradation_control_gel_n2_3.tif]

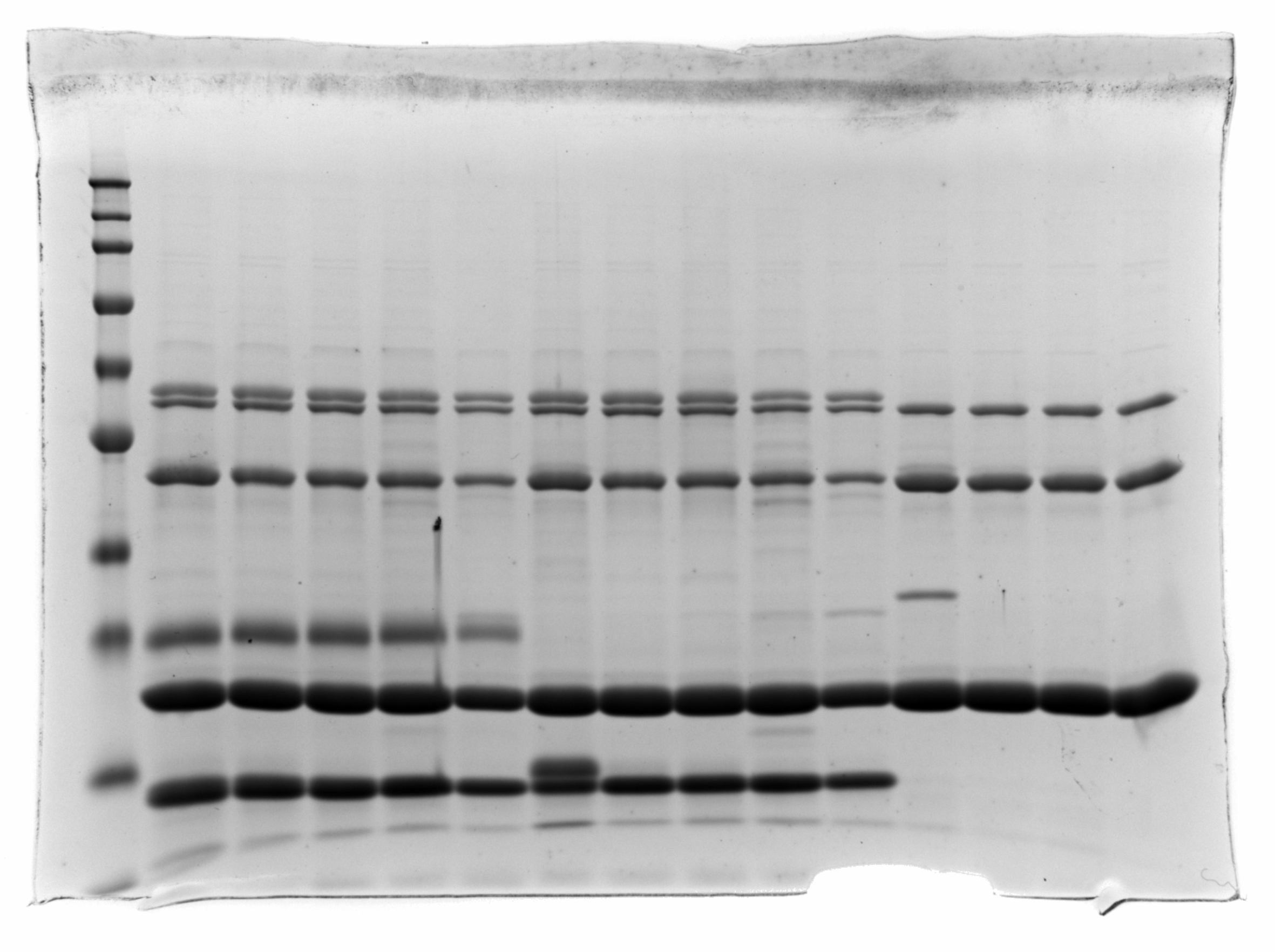

Supplement: Supplementary file 7 — Source data Fig. 4 [file 44319_2025_510_MOESM7_ESM.zip › Fig4/Fig4F/GroTAC/n1/In_vitro_degradation_GroTAC_gel_n1_1.tif]

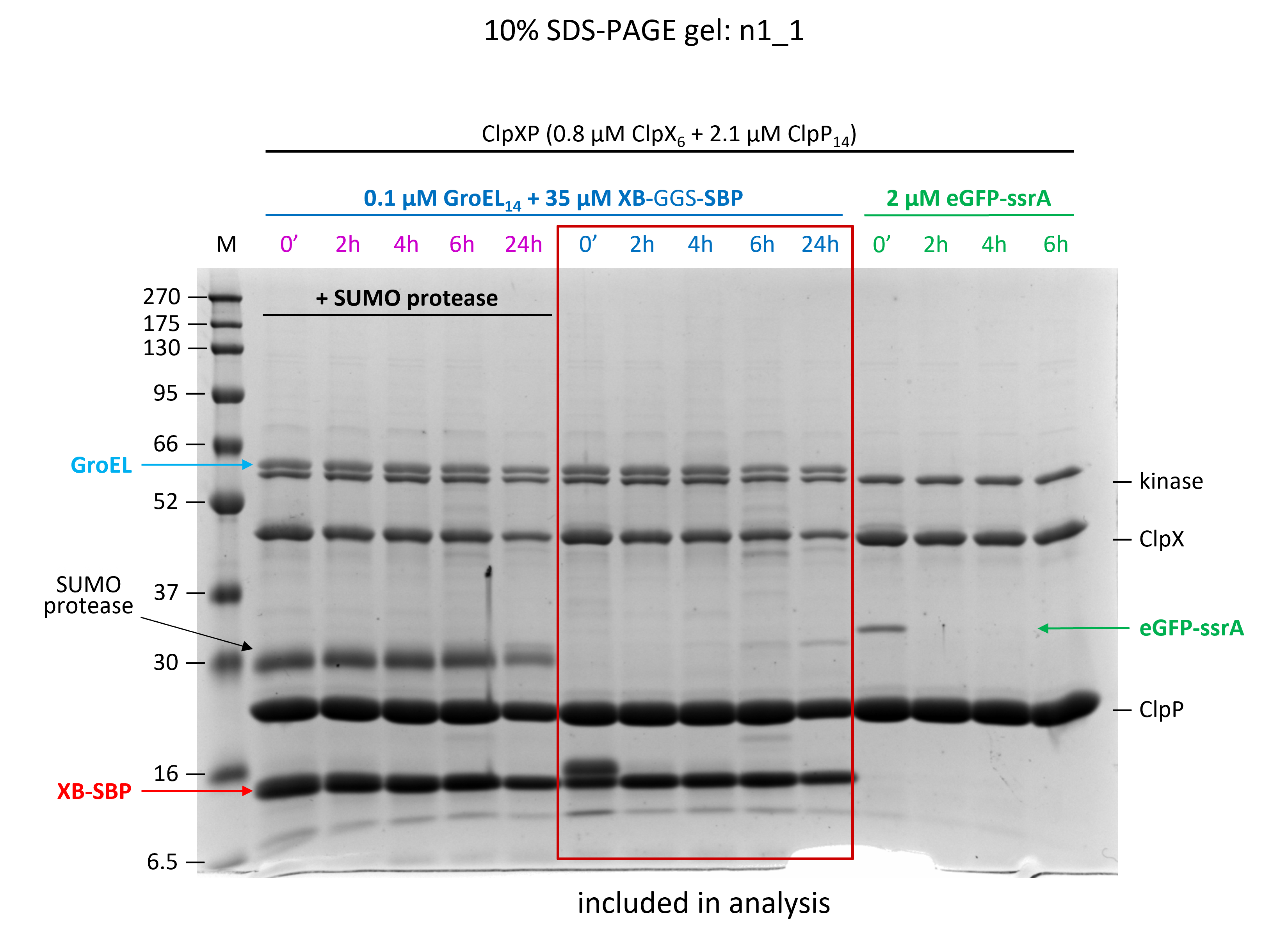

Supplement: Supplementary file 7 — Source data Fig. 4 [file 44319_2025_510_MOESM7_ESM.zip › Fig4/Fig4F/GroTAC/n1/In_vitro_degradation_GroTAC_gel_n1_1_label.tif]

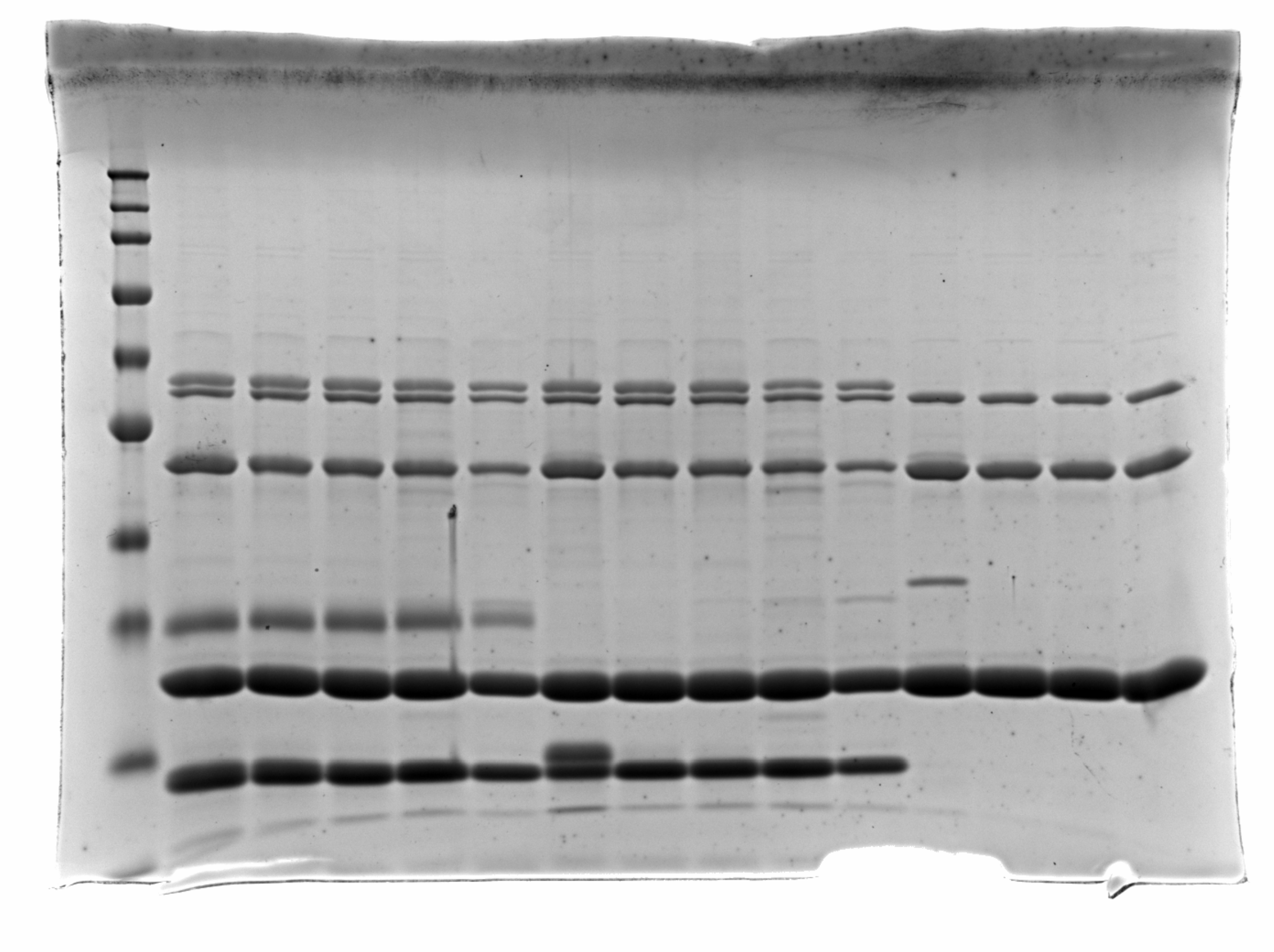

Supplement: Supplementary file 7 — Source data Fig. 4 [file 44319_2025_510_MOESM7_ESM.zip › Fig4/Fig4F/GroTAC/n1/In_vitro_degradation_GroTAC_gel_n1_2.tif]

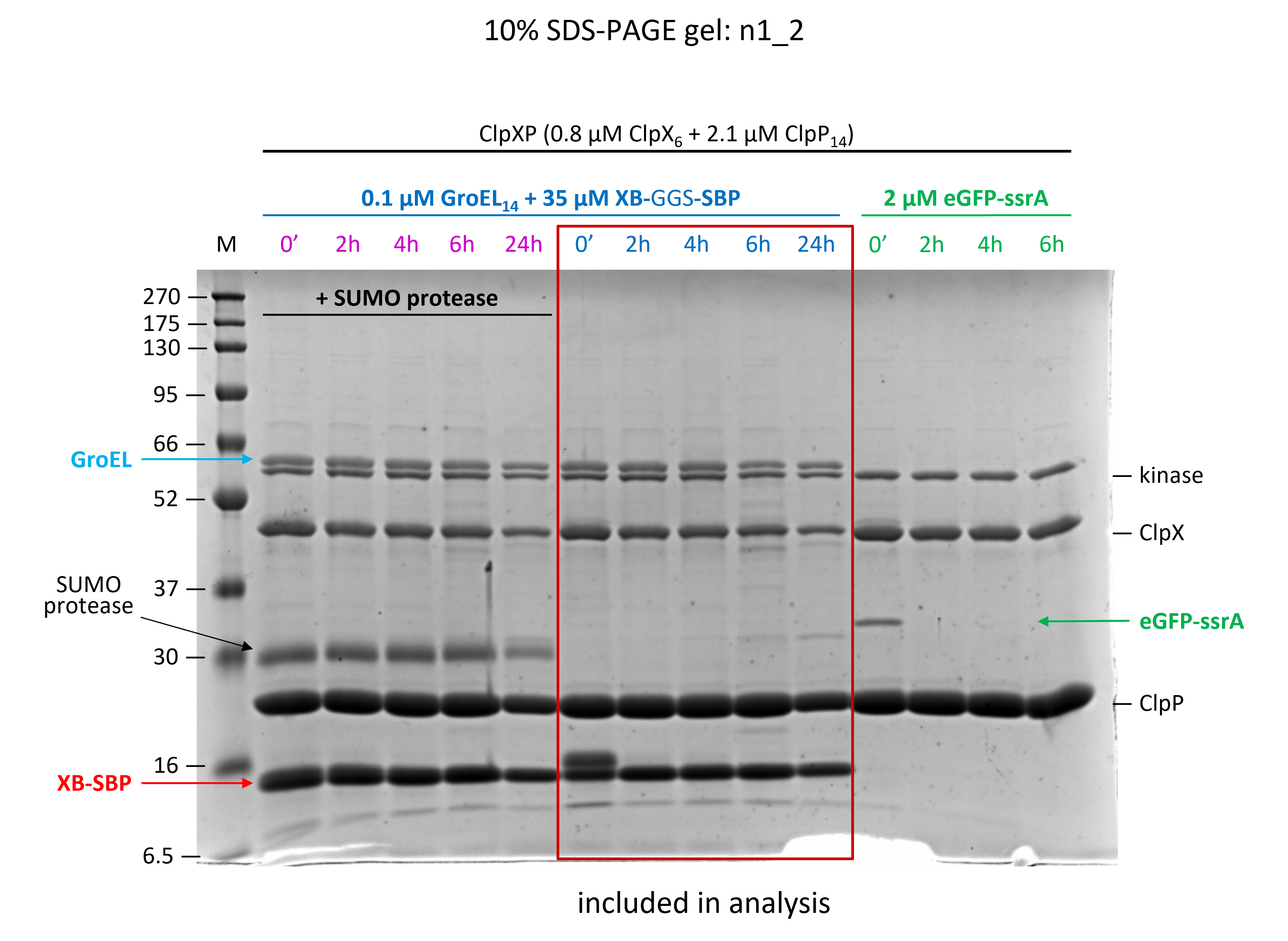

Supplement: Supplementary file 7 — Source data Fig. 4 [file 44319_2025_510_MOESM7_ESM.zip › Fig4/Fig4F/GroTAC/n1/In_vitro_degradation_GroTAC_gel_n1_2_label.tif]

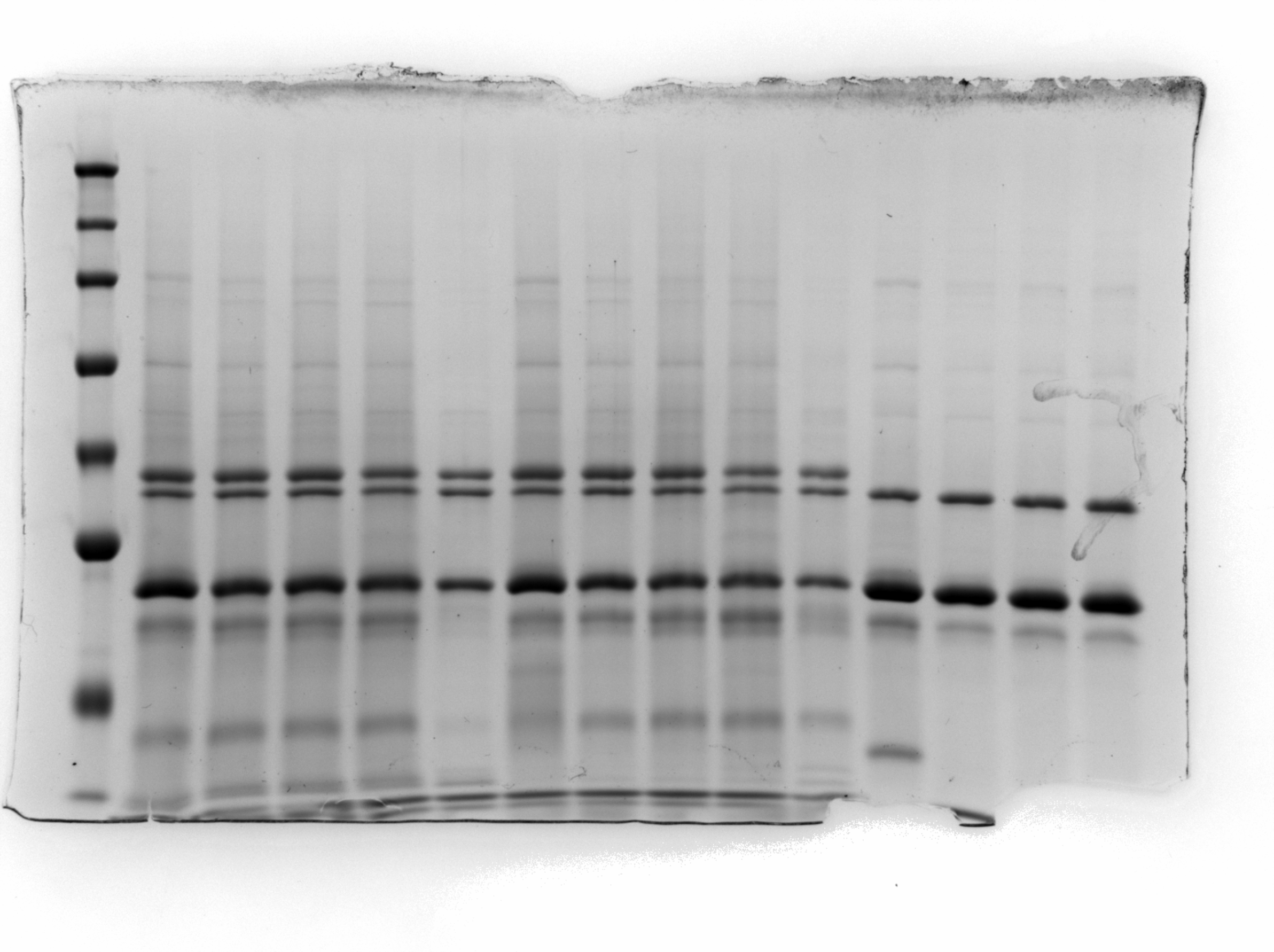

Supplement: Supplementary file 7 — Source data Fig. 4 [file 44319_2025_510_MOESM7_ESM.zip › Fig4/Fig4F/GroTAC/n2/In_vitro_degradation_GroTAC_gel_n2.tif]

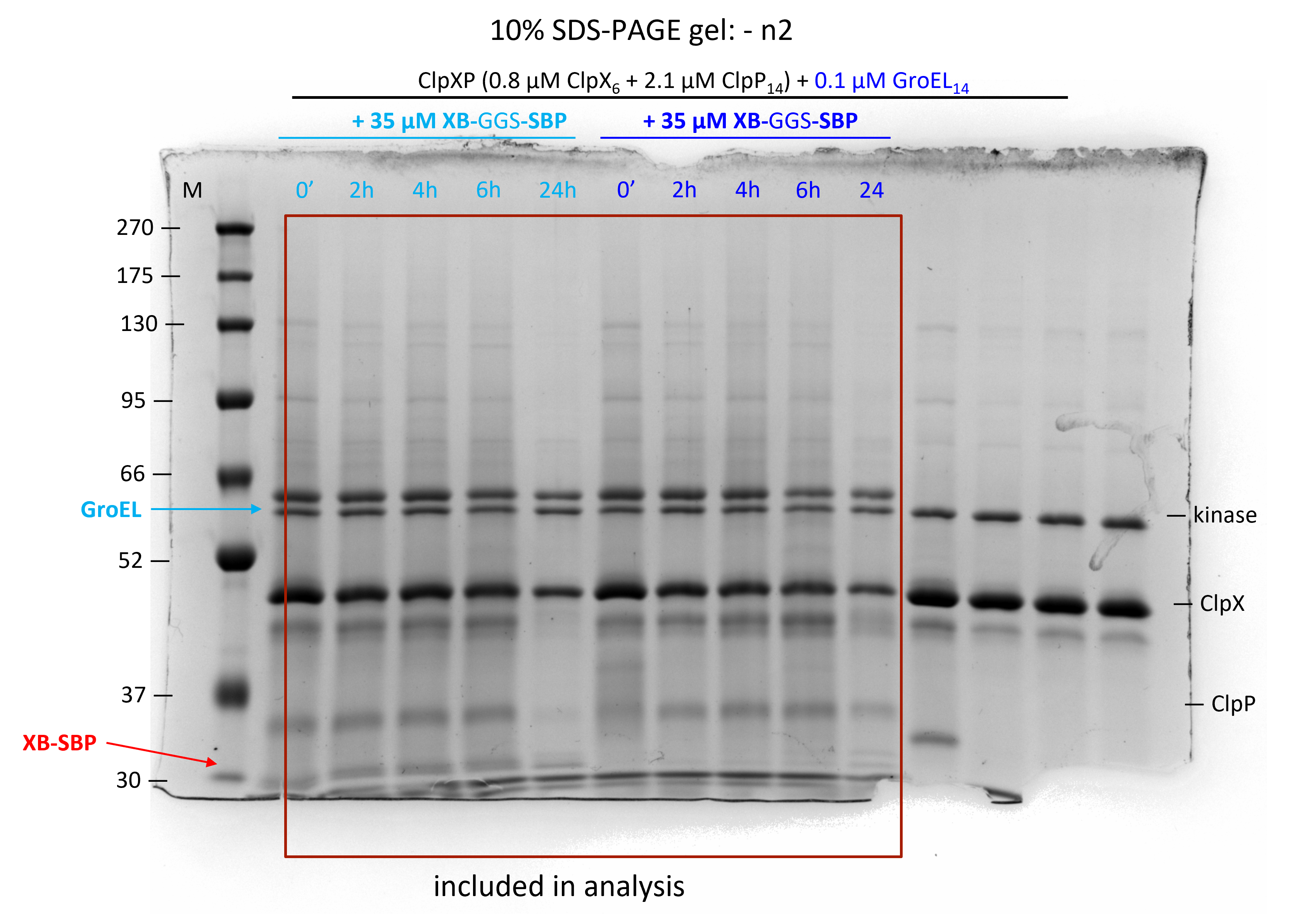

Supplement: Supplementary file 7 — Source data Fig. 4 [file 44319_2025_510_MOESM7_ESM.zip › Fig4/Fig4F/GroTAC/n2/In_vitro_degradation_GroTAC_gel_n2_label.tif]

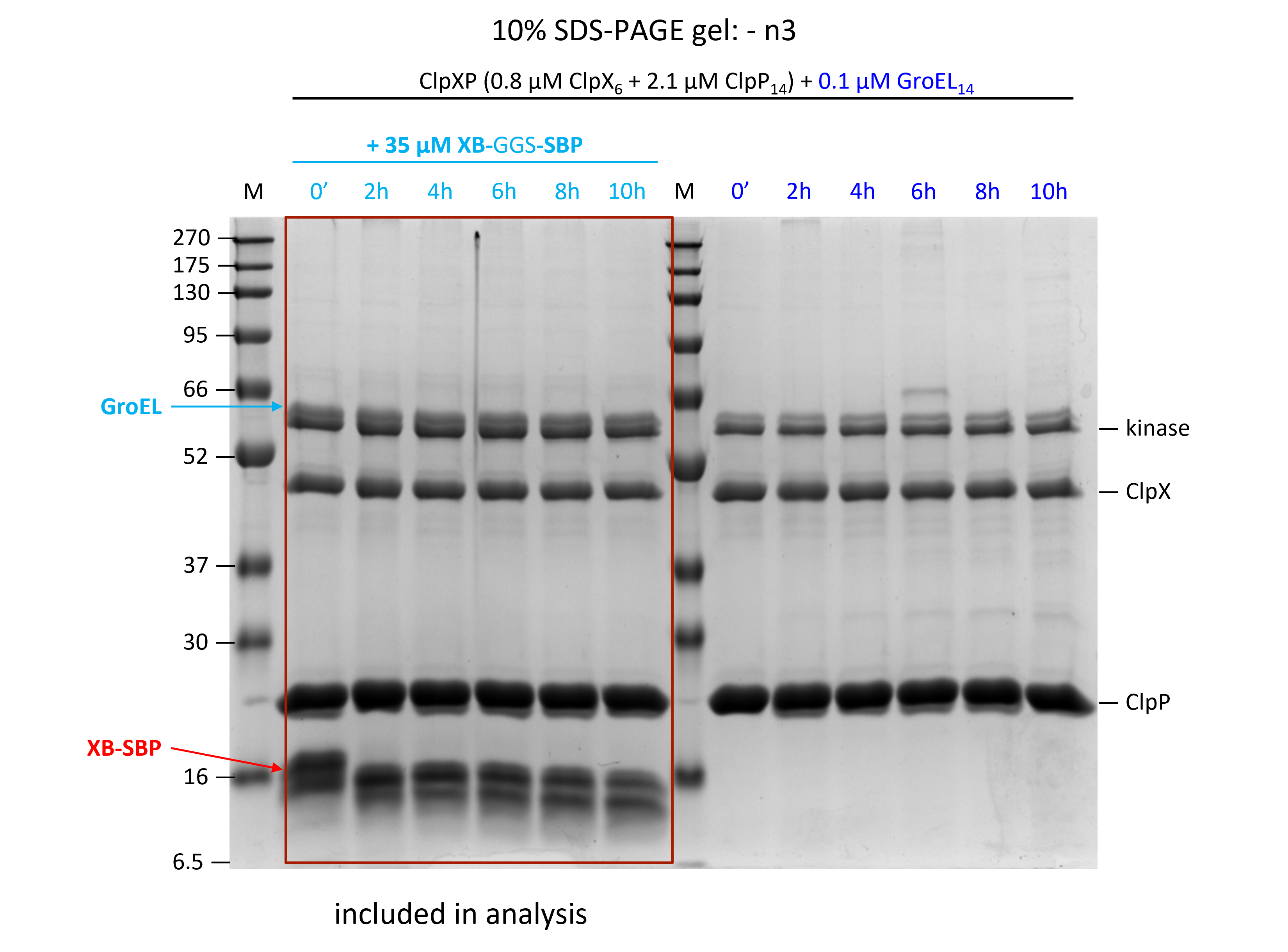

Supplement: Supplementary file 7 — Source data Fig. 4 [file 44319_2025_510_MOESM7_ESM.zip › Fig4/Fig4F/GroTAC/n3/In_vitro_degradation_GroTAC_gel_n3_label.tif]

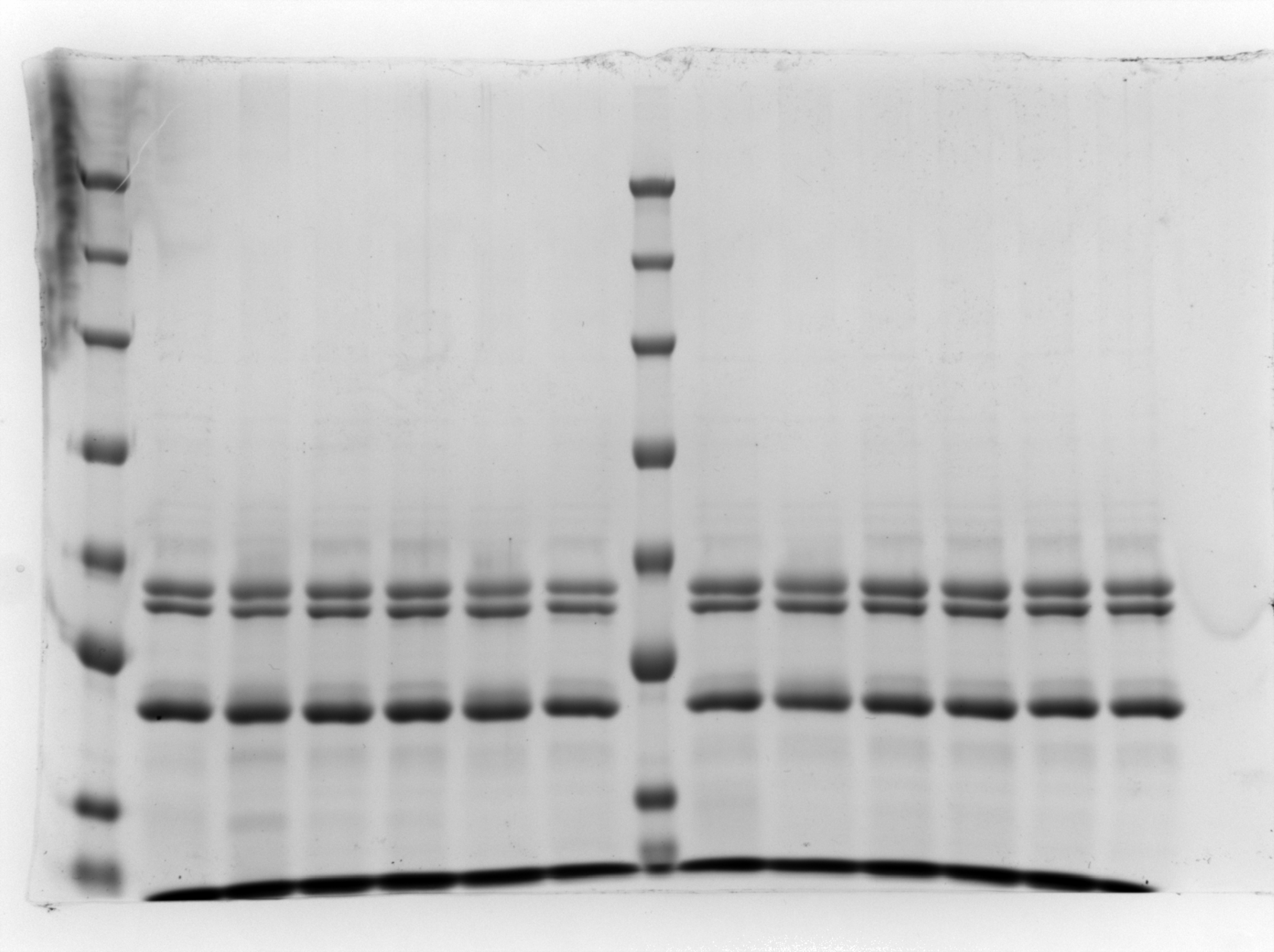

Supplement: Supplementary file 7 — Source data Fig. 4 [file 44319_2025_510_MOESM7_ESM.zip › Fig4/Fig4F/GroTAC/n4/In_vitro_degradation_GroTAC_gel_n4.tif]

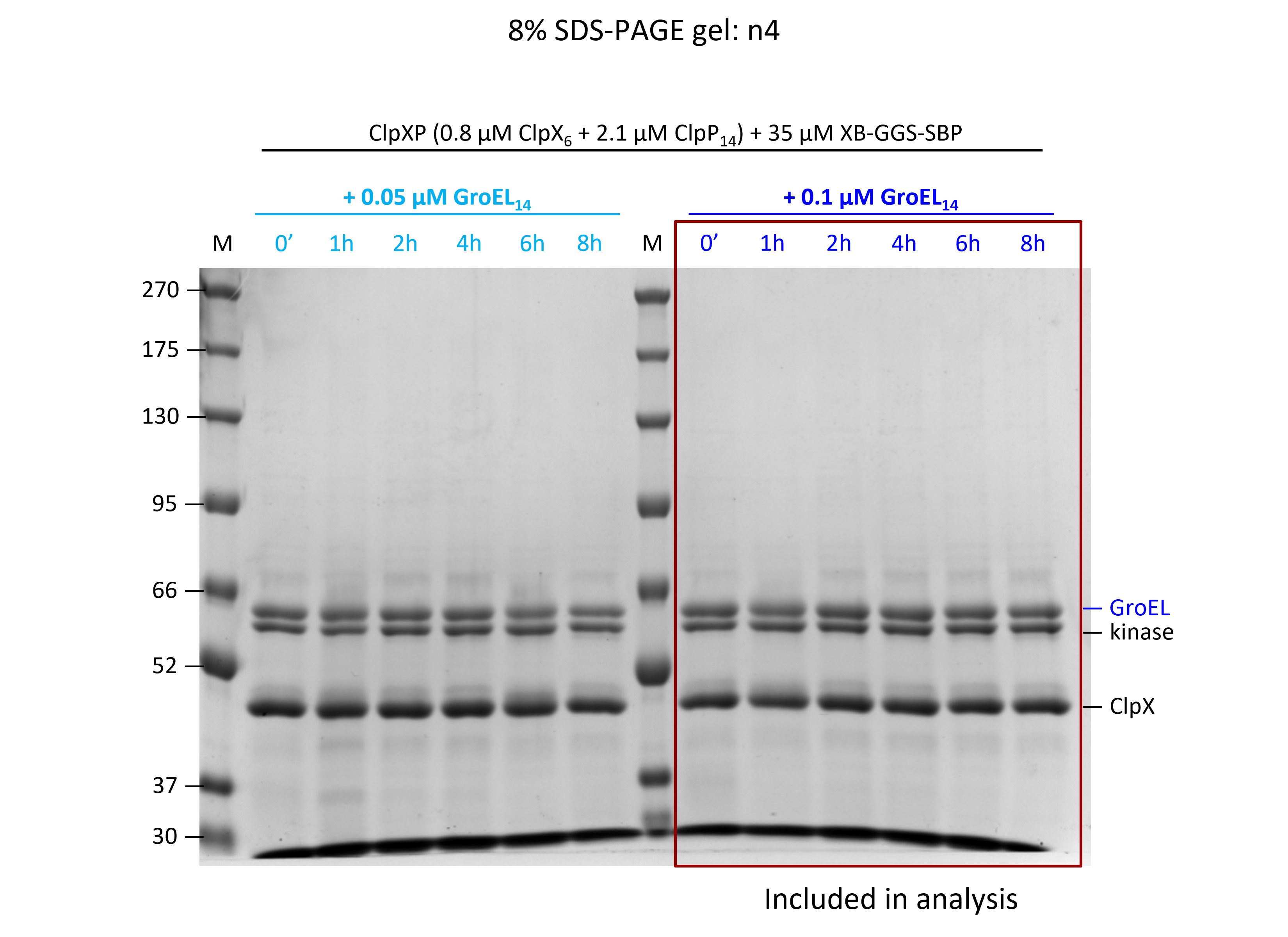

Supplement: Supplementary file 7 — Source data Fig. 4 [file 44319_2025_510_MOESM7_ESM.zip › Fig4/Fig4F/GroTAC/n4/In_vitro_degradation_GroTAC_n4_label.tif]

# 8% SDS-PAGE gel: n4

ClpXP (0.8  $\mu$ M ClpX<sub>6</sub> + 2.1  $\mu$ M ClpP<sub>14</sub>) + 35  $\mu$ M XB-GGS-SBP

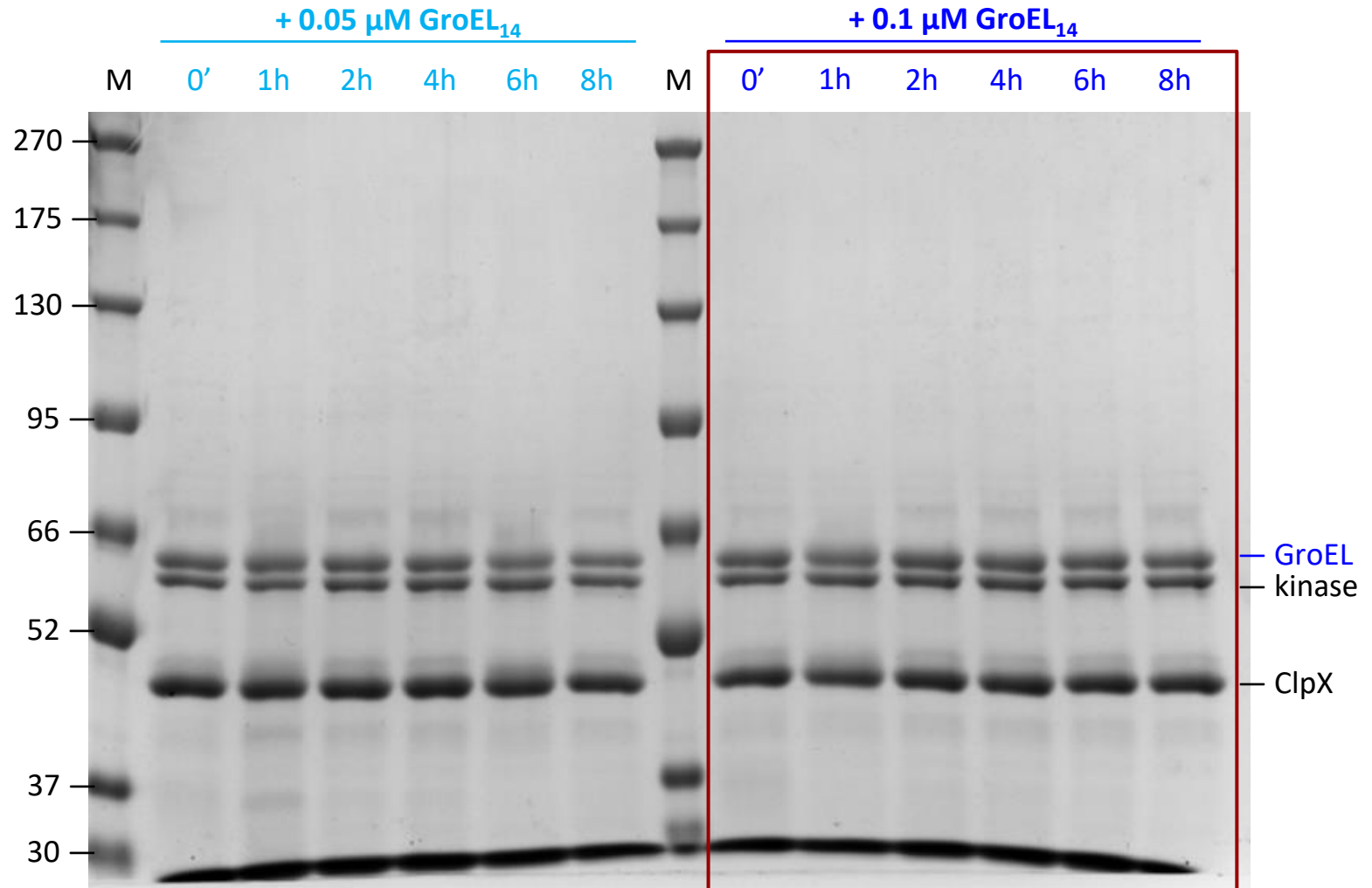

Included in analysis

Supplement: Supplementary file 7 — Source data Fig. 4 [file 44319_2025_510_MOESM7_ESM.zip › Fig4/Fig4F/GroTAC/n4/In_vitro_degradation_GroTAC_n4_labelled_gels.pdf]

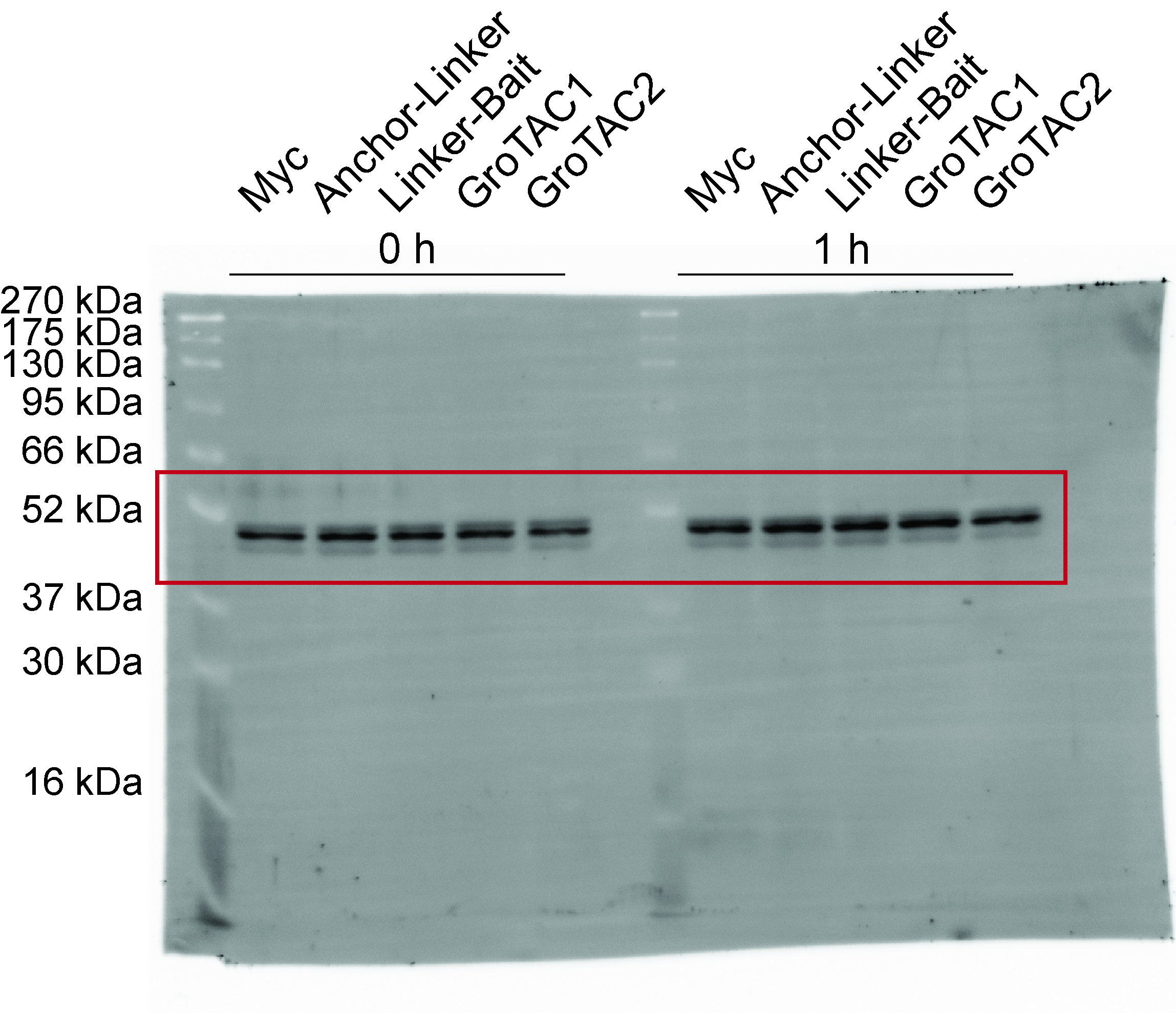

Supplement: Supplementary file 10 — Figure EV3 Source Data [file 44319_2025_510_MOESM10_ESM.zip › EV3/EV3A/western_blot_0h_1h_enolase_uncropped_labelled.tif]

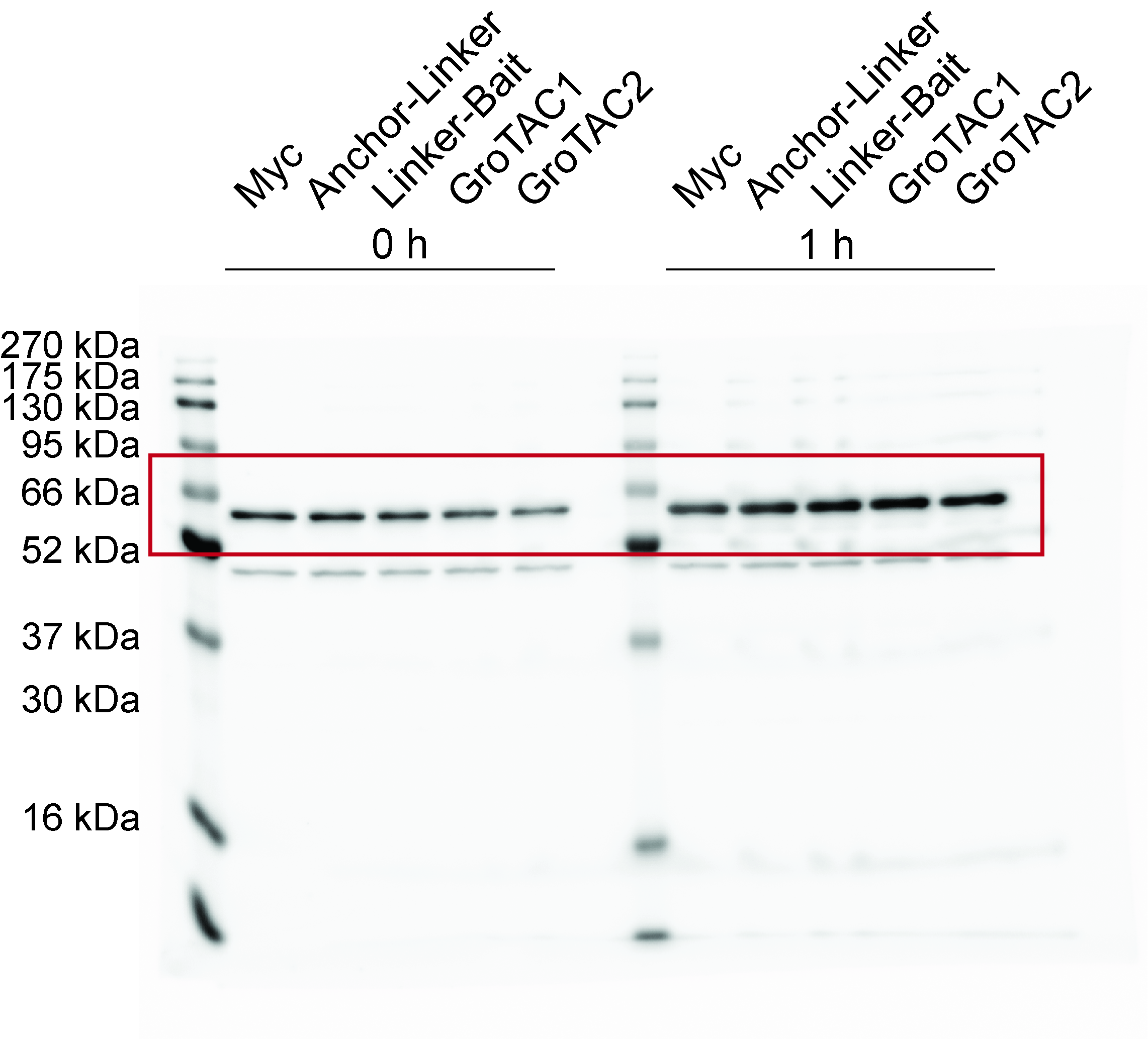

Supplement: Supplementary file 10 — Figure EV3 Source Data [file 44319_2025_510_MOESM10_ESM.zip › EV3/EV3A/western_blot_0h_1h_GroEL_uncropped_labelled.tif]

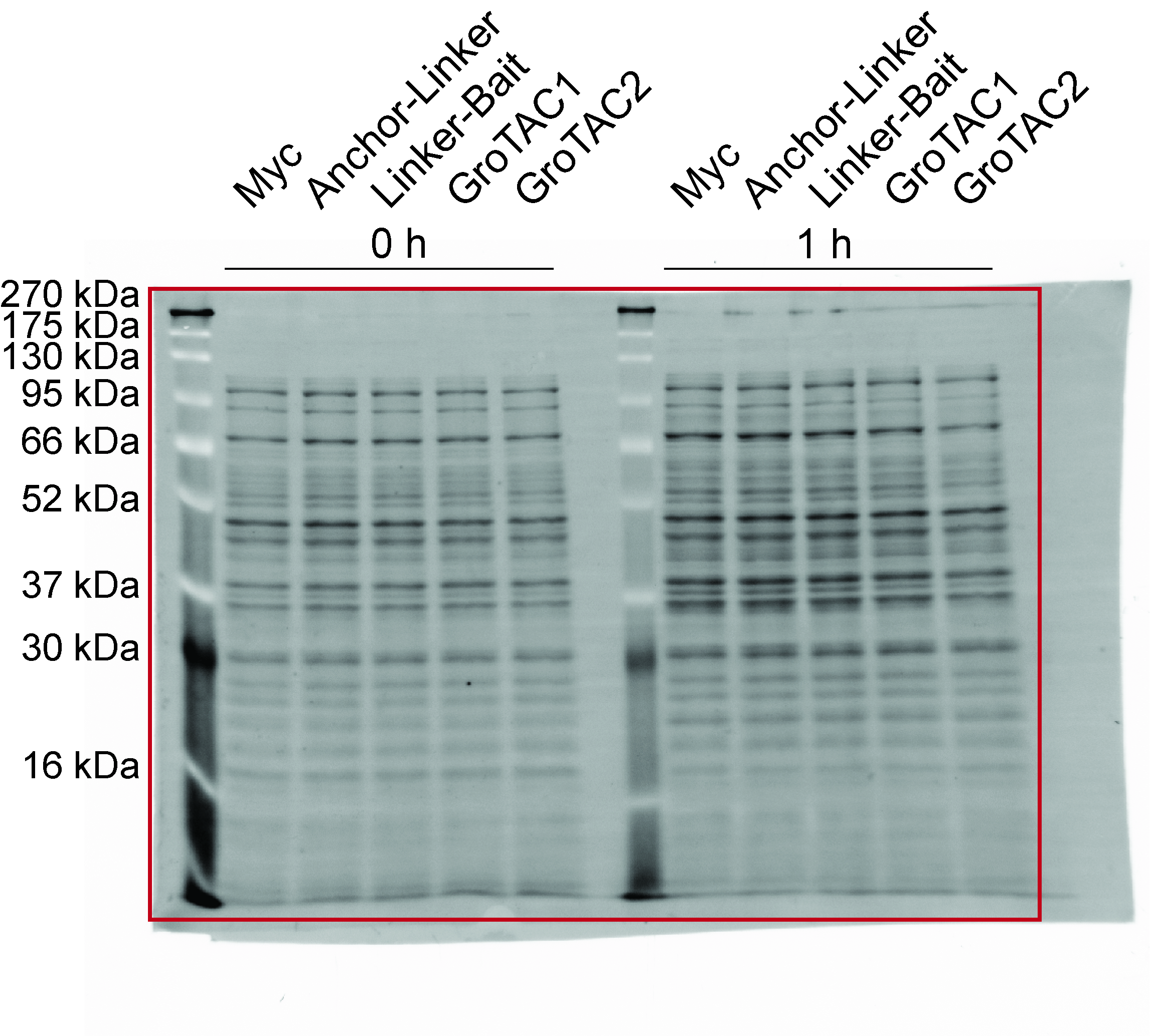

Supplement: Supplementary file 10 — Figure EV3 Source Data [file 44319_2025_510_MOESM10_ESM.zip › EV3/EV3A/western_blot_0h_1h_stainfree_uncropped_labelled.tif]

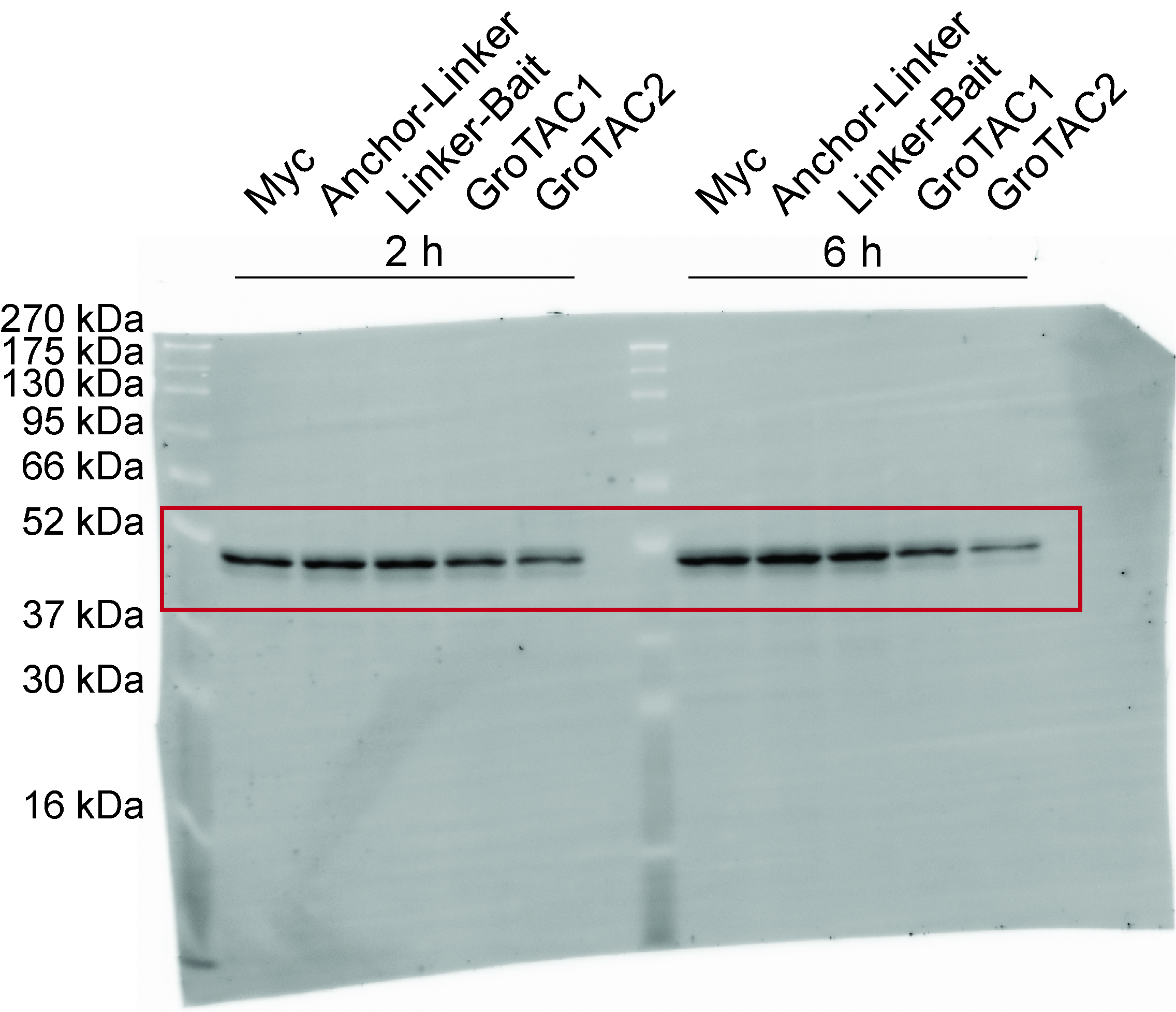

Supplement: Supplementary file 10 — Figure EV3 Source Data [file 44319_2025_510_MOESM10_ESM.zip › EV3/EV3B/western_blot_2h_6h_enolase_uncropped_labelled.tif]

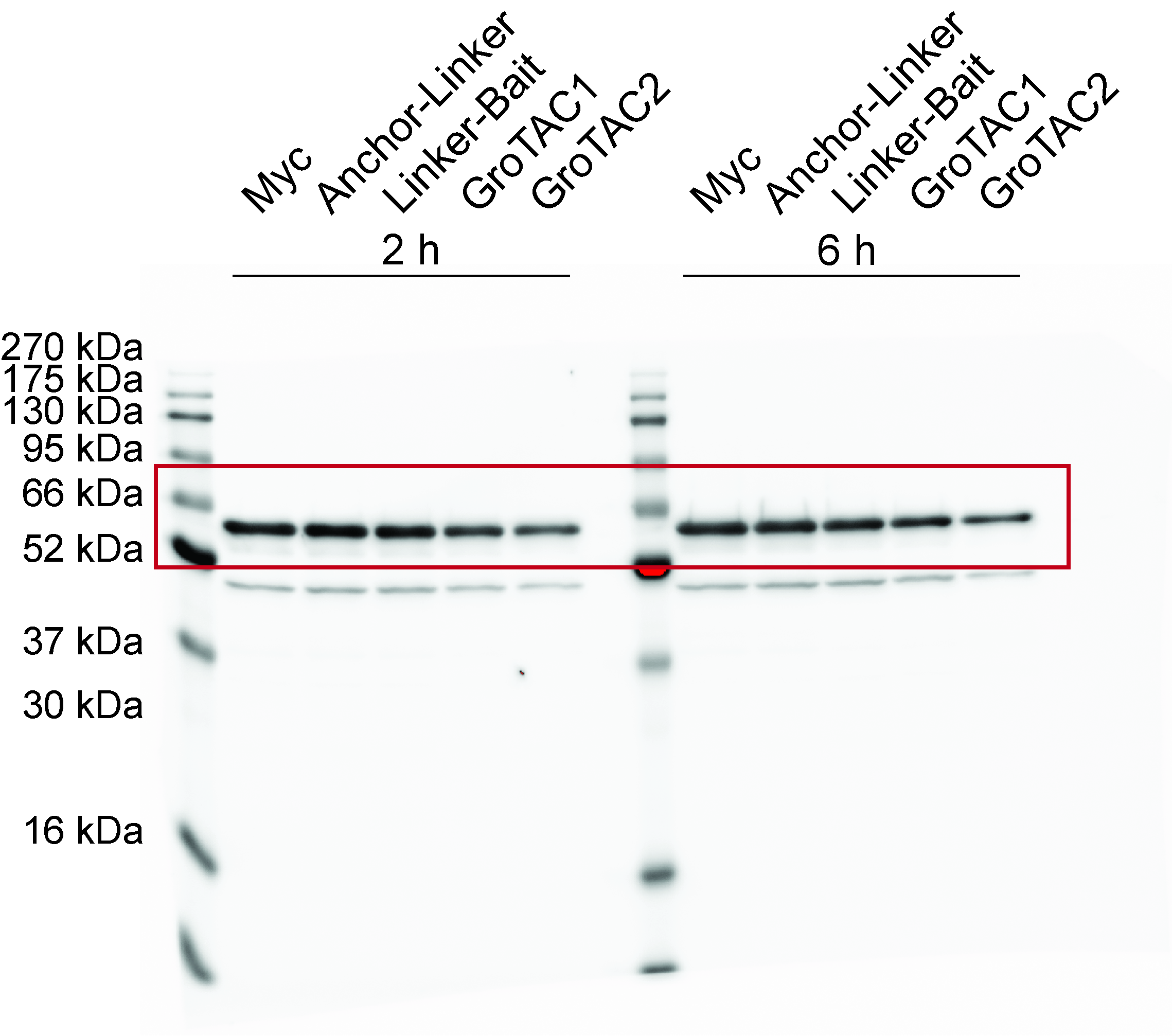

Supplement: Supplementary file 10 — Figure EV3 Source Data [file 44319_2025_510_MOESM10_ESM.zip › EV3/EV3B/western_blot_2h_6h_GroEL_uncropped_labelled.tif.tif]

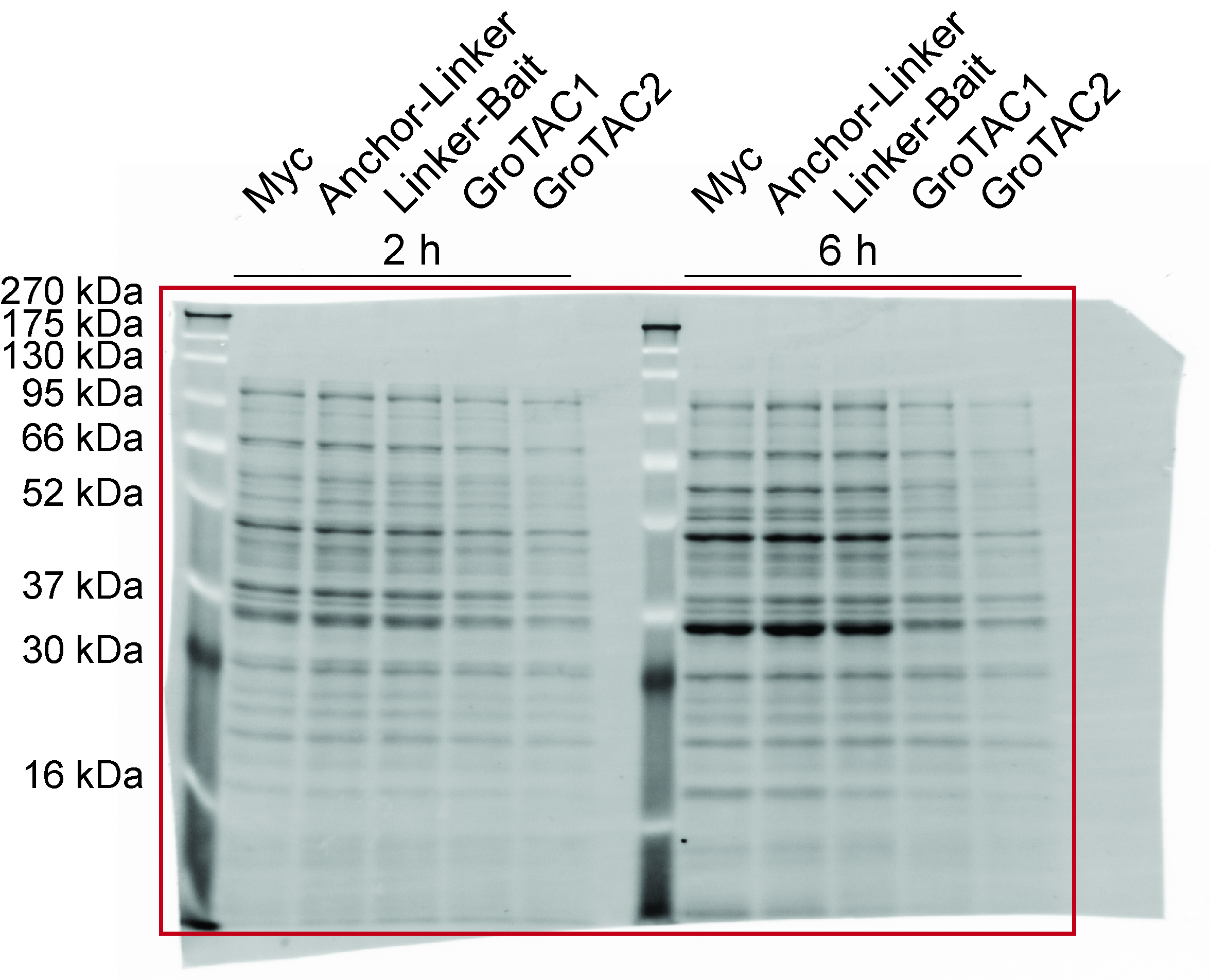

Supplement: Supplementary file 10 — Figure EV3 Source Data [file 44319_2025_510_MOESM10_ESM.zip › EV3/EV3B/western_blot_2h_6h_stainfree_uncropped_labelled.tif]
