# Supplementary material for: Targeted protein degradation in Escherichia coli using CLIPPERs
Source: EMBO Rep. 2025 Jun 25;26(16):3994–4016. doi: 10.1038/s44319-025-00510-9 (PMC12373786; doi:10.1038/s44319-025-00510-9)

10% SDS-PAGE gel: n1_1

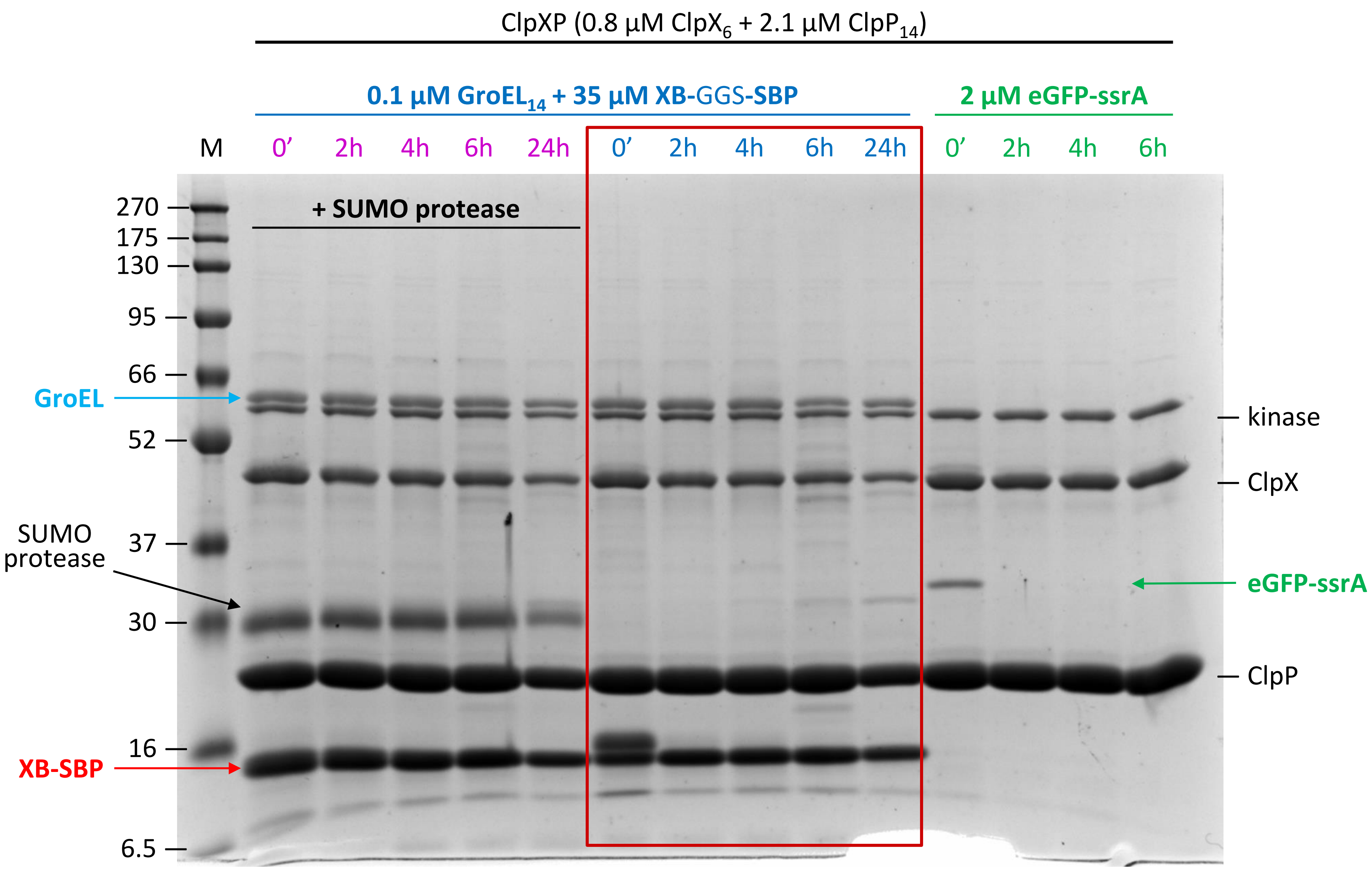

included in analysis

10% SDS-PAGE gel: n1_2

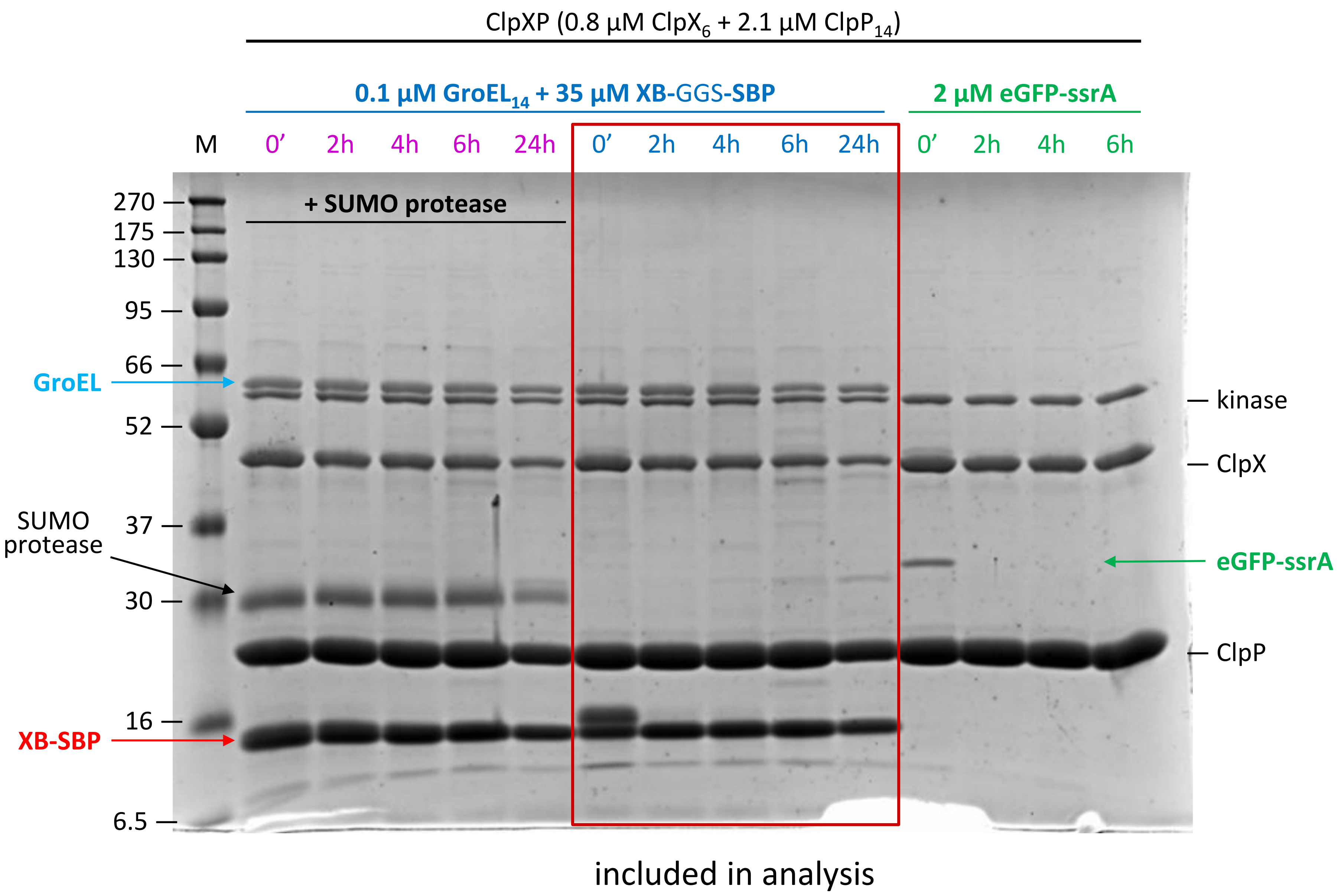

Supplement: Supplementary file 7 — Source data Fig. 4 [file 44319_2025_510_MOESM7_ESM.zip › Fig4/Fig4F/GroTAC/n1/In_vitro_degradation_GroTAC_n1_labelled_gels.pdf]
