# Supplementary material for: Targeted protein degradation in Escherichia coli using CLIPPERs
Source: EMBO Rep. 2025 Jun 25;26(16):3994–4016. doi: 10.1038/s44319-025-00510-9 (PMC12373786; doi:10.1038/s44319-025-00510-9)
Supplement: Supplementary file 15 — Expanded View Figures [file 44319_2025_510_MOESM15_ESM.pdf]

Expanded View Figures

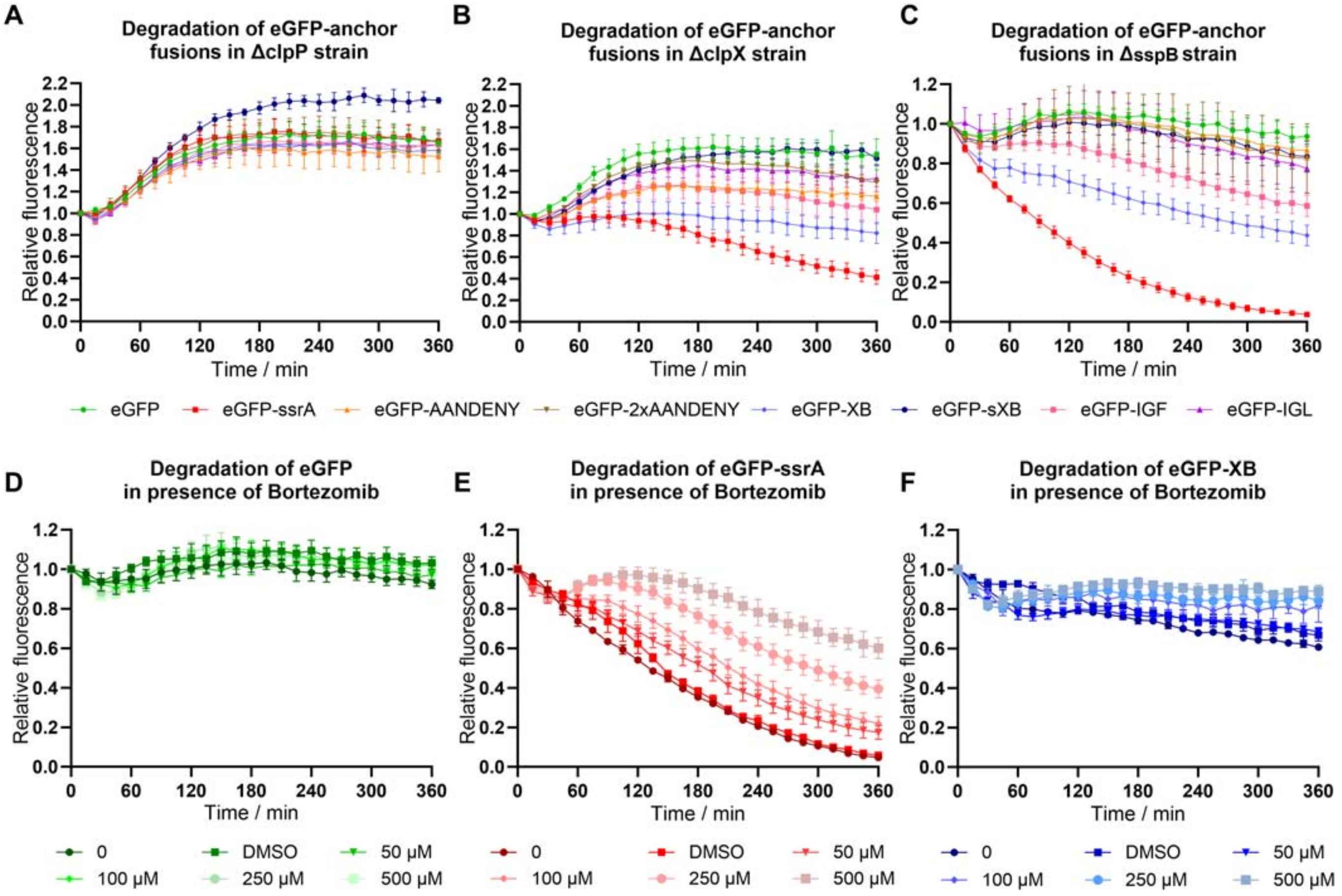

Figure EV1. Degradation of the eGFP-anchor fusion proteins in bacteria.

Degradation was performed in *E. coli* deletion mutant strains (**A**) ΔclpP, (**B**) ΔclpX, and (**C**) ΔsspB. Degradation was performed in *E. coli* BW25113 strain for (**D**) untagged eGFP (negative control), (**E**) eGFP-ssrA (positive control), and (**F**) eGFP-XB. The curves represent mean values from 3 biological repeats (averaged for clarity) with error bars representing SEM. Source data are available online for this figure.

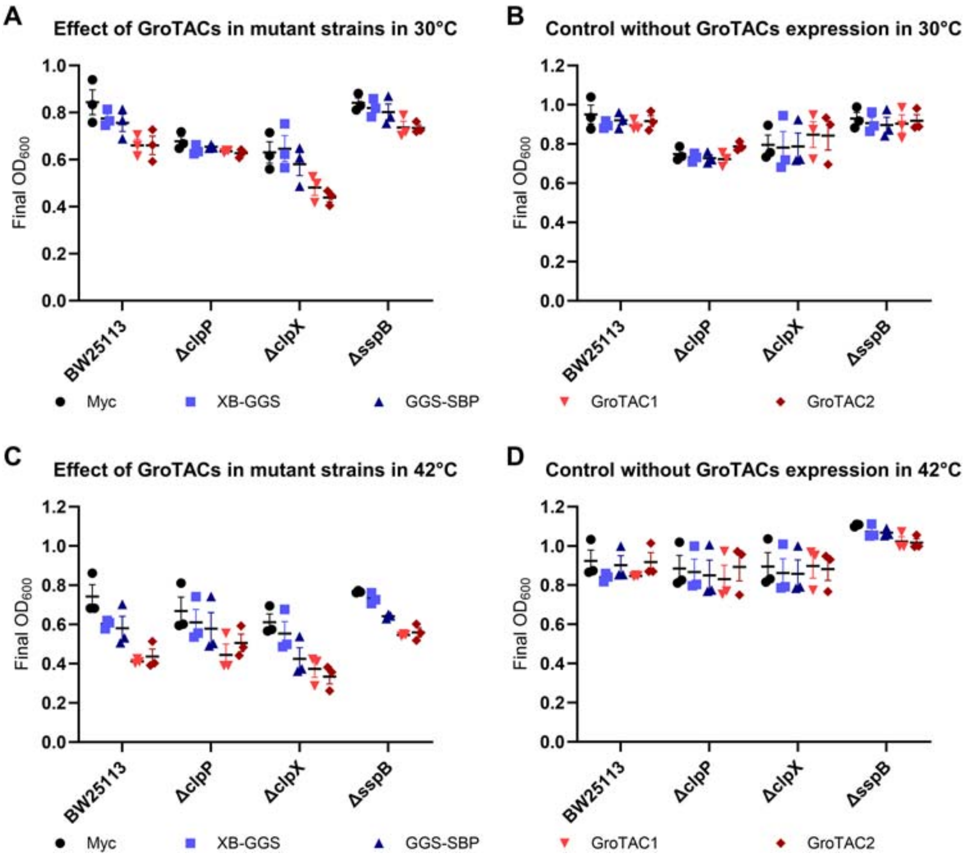

Figure EV2. The effect of protease component mutations on the GroTACs-mediated growth inhibition.

The expression of GroTACs was tested in *E. coli* deletion mutant strains for their effect on final culture OD_{600} after 16 h of culturing (**A**, **C**) in presence, or (**B**, **D**) in absence of expression-inducing arabinose at 30 °C (**A**, **B**) or 42 °C (**C**, **D**). The horizontal lines represent the mean from three biological replicates and the error bars represent SEM. Source data are available online for this figure.

 © The Author(s)

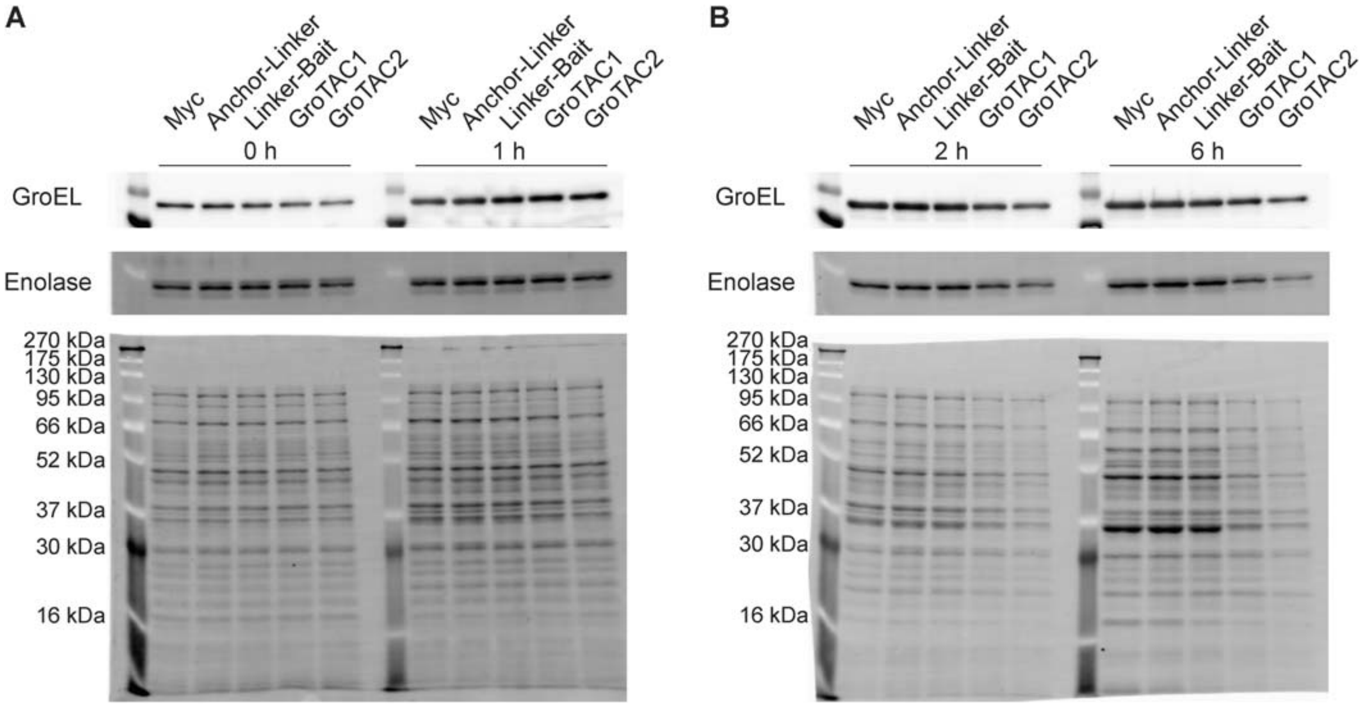

Figure EV3. The effect or GroTACs expression on proteins in *E. coli.*

The figure presents representative western blots of GroEL and enolase levels and total protein on PVDF membranes visualised by the stain-free method (Ladner et al, 2004). Protein levels were measured (**A**) 0 h and 1 h after induction and (**B**) 2 h and 6 h after induction. Source data are available online for this figure.

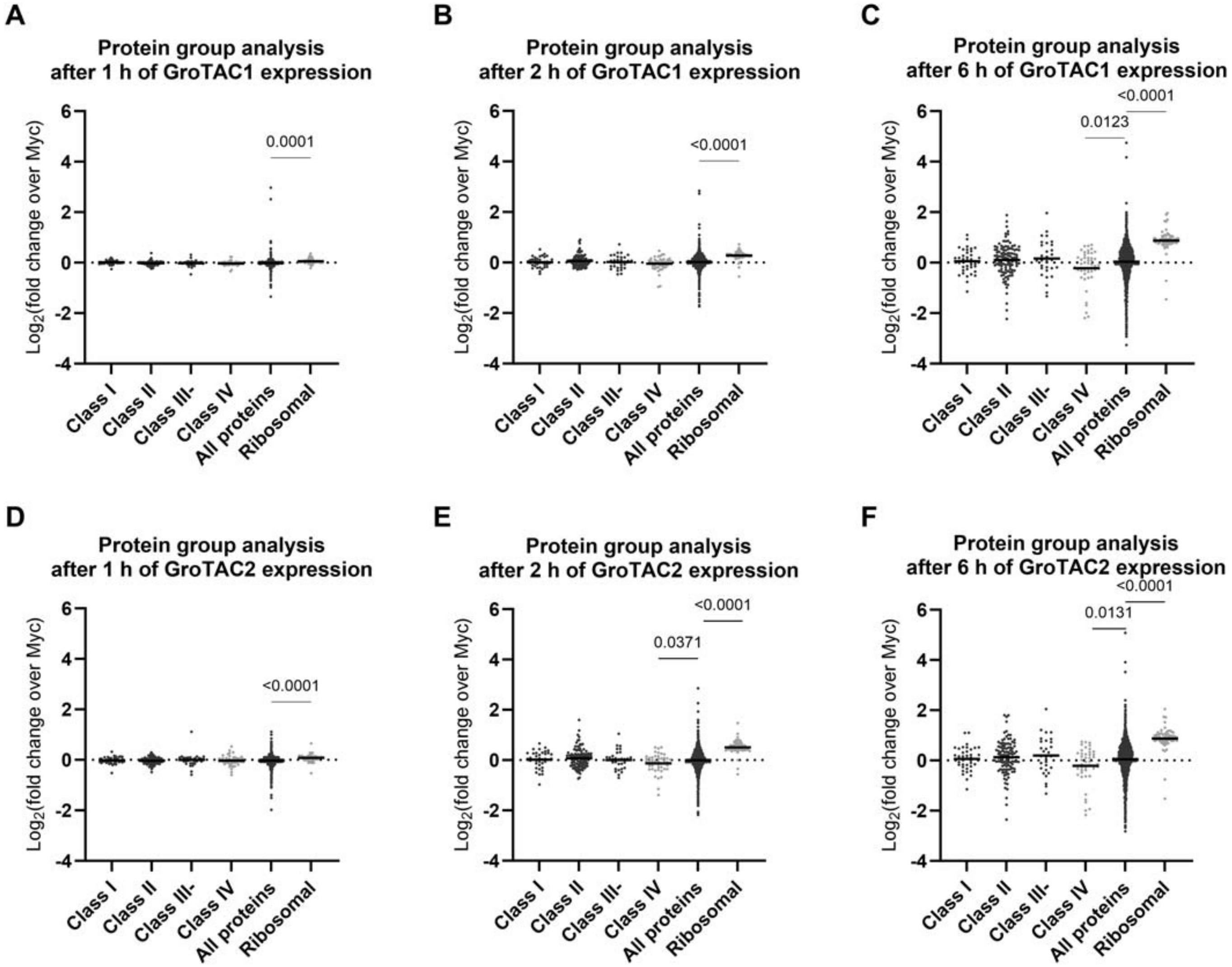

Figure EV4. GroTAC-induced changes in specific protein groups.

(Group analysis of relative changes in levels of GroEL substrates and ribosomal proteins induced by GroTAC1 (**A–C**) and GroTAC2 (**D–F**) after 1 h (**A**, **D**) 2 h (**B**, **E**) and 6 h (**C**, **F**) of peptide expression. Statistically significant changes (two-sided, unpaired Student's *t* test) are denoted by *P* values, and the mean value in each group is indicated by a horizontal line. Data for TMT-MS were collected for three biological replicates. Source data are available online for this figure.

 © The Author(s)

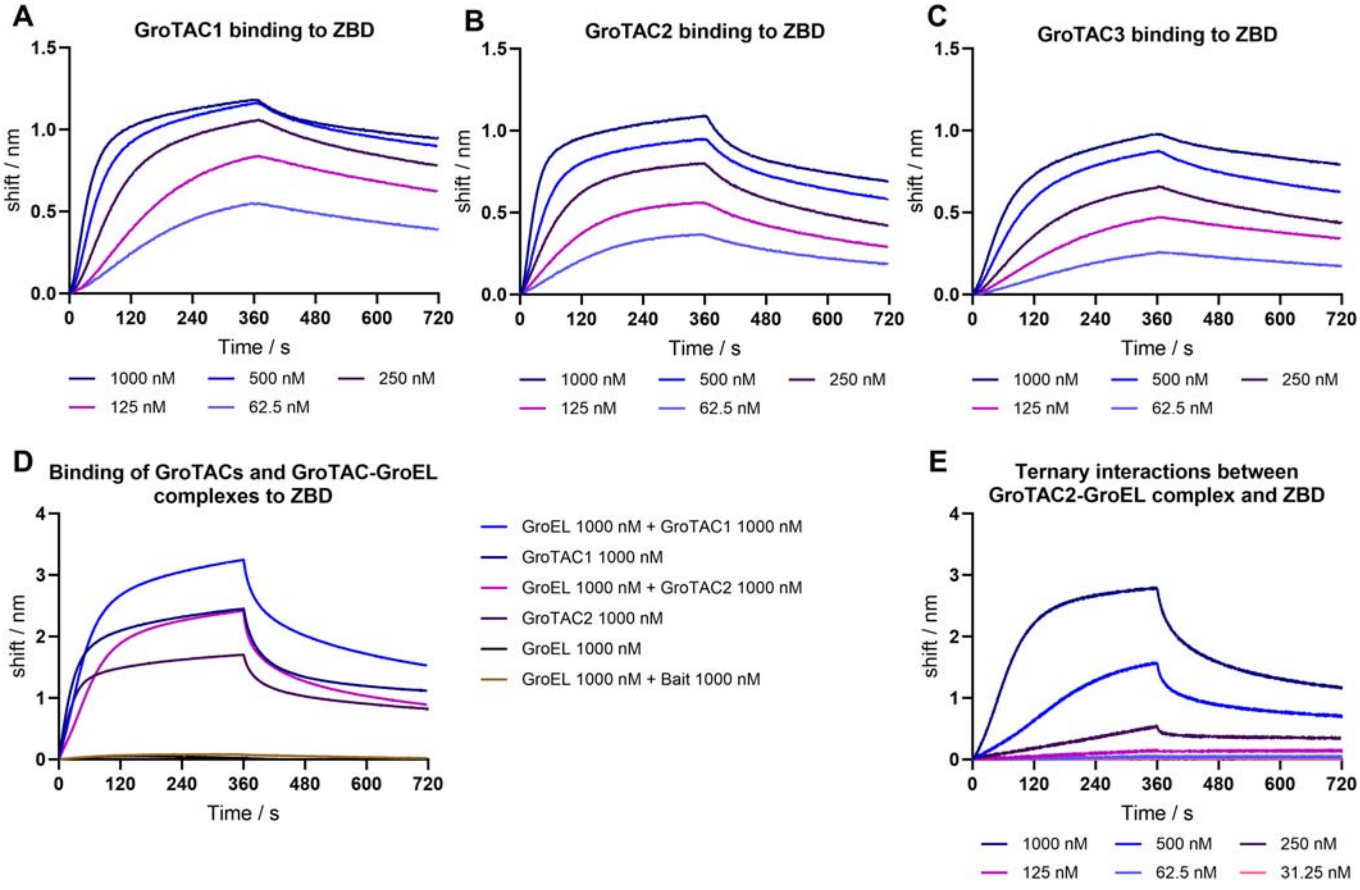

Figure EV5. Binding of untagged GroTACs to ZBD.

Sensograms representing BLI measurements. (**A**) Binding of the synthetic GroTAC1 peptide to immobilised His-ZBD. (**B**) Binding of the synthetic GroTAC2 peptide to immobilised His-ZBD. (**C**) Binding of GroTAC3 to immobilised His-ZBD. (**D**) Comparison of binding of GroEL, synthetic GroTAC peptides or their complexes to immobilised His-ZBD. "Bait" is a synthetic control peptide consisting of a "Linker-Bait" fusion. (**E**) Binding of GroEL-GroTAC2 complex to immobilised His-ZBD using a fixed GroEL concentration (1000 nM) and increasing concentrations of the synthetic GroTAC2 peptide in the preincubated GroTAC2-GroEL mixture. Source data are available online for this figure.
